# Population pharmacodynamic modeling of intramuscular and oral dexamethasone and betamethasone effects on six biomarkers with circadian complexities in Indian women

**DOI:** 10.1007/s10928-021-09755-y

**Published:** 2021-05-05

**Authors:** Wojciech Krzyzanski, Mark A. Milad, Alan H. Jobe, Thomas Peppard, Robert R. Bies, William J. Jusko

**Affiliations:** 1 School of Pharmacy and Pharmaceutical Sciences, State University of New York, University at Buffalo, Buffalo, NY USA; 2 Milad Pharmaceutical Consulting LLC, Plymouth, MI USA; 3 Division of Pulmonary Biology, Cincinnati Children’s Hospital Medical Center, University of Cincinnati, Cincinnati, OH USA; 4 Certara, Inc, Princeton, NJ USA; 5 Institute for Computational Data Science, University a Buffalo, Buffalo, NY USA

**Keywords:** Pharmacokinetics, Pharmacodynamics, Population modeling, Dexamethasone, Betamethasone, Adrenal suppression, Cell trafficking

## Abstract

Population pharmacokinetic/pharmacodynamic (PK/PD) analysis was performed for extensive data for differing dosage forms and routes for dexamethasone (DEX) and betamethasone (BET) in 48 healthy nonpregnant Indian women in a partial and complex cross-over design. Single doses of 6 mg dexamethasone phosphate (DEX-P), betamethasone phosphate (BET-P), or 1:1 mixture of betamethasone phosphate and acetate (BET-PA) were administered orally (PO) or intramuscularly (IM) where each woman enrolled in a two-period cross-over study. Plasma concentrations collected over 96 h were described with a two-compartment model with differing PO and IM first-order absorption inputs. Overall, BET exhibited slower clearance, similar volume of distribution, faster absorption, and longer persistence than DEX with BET acetate producing extremely slow absorption but full bioavailability of BET. Six biomarkers were assessed over a 24-h baseline period with four showing circadian rhythms with complex baselines. These baselines and the strong responses seen after drug dosing were fitted with various indirect response models using the Laplace estimation methods in NONMEM 7.4. Both the PK and six biomarker responses were well-described with modest variability likely due to the homogeneous ages, weights, and ethnicities of the women. The drugs either inhibited or stimulated the influx processes with some models requiring joint inclusion of drug effects on circadian cortisol suppression. The biomarkers and order of sensitivity (lowest *IC*_*50*_*/SC*_*50*_ to highest) were: cortisol, T-helper cells, basophils, glucose, neutrophils, and T-cytotoxic cells. DEX sensitivities were generally greater than BET with corresponding mean ratios for these biomarkers of 2.86, 1.27, 1.72, 1.27, 2.69, and 1.06. Overall, the longer PK (e.g. half-life) of BET, but lesser PD activity (e.g. higher *IC*_*50*_), produces single-dose response profiles that appear quite similar, except for the extended effects from BET-PA. This comprehensive population modeling effort provides the first detailed comparison of the PK profiles and six biomarker responses of five commonly used dosage forms of DEX and BET in healthy women.

**Supplementary Information:**

The online version of this article (10.1007/s10928-021-09755-y) contains supplementary material, which is available to authorized users.

## Introduction

The glucocorticoids (GC) dexamethasone (DEX) and betamethasone (BET) are enantiomers with similar therapeutic indications, including use in treating women at risk of preterm delivery and resultant respiratory distress syndrome in their neonates [1, 2]. Current recommended treatment by the World Health Organization (WHO) comprises dexamethasone phosphate (DEX-P) given intramuscularly (IM) as four doses of 6 mg given at 12-h intervals, betamethasone phosphate (BET-P) given IM as two doses of 12 mg given at a 24-h interval, and the one-to-one mixture of betamethasone phosphate and acetate (BET-PA) as two doses of 12 mg given IM at a 24-h interval [3].

The pharmacokinetics and pharmacodynamics (PK/PD) of oral (PO), IM, and IV DEX have been well studied in both animals and humans, while much less information is available regarding the properties of BET. Our recent study of single doses of IM and PO DEX-P and BET-P plus the IM BET-PA mixture employed blood stabilization of esters, 96-h blood sampling, and LC–MS/MS methodology to carefully assess the PK of these steroid formulations in 48 healthy Indian women [4]. However, the preliminary report was limited to a noncompartmental analysis (NCA) of the PK/PD data. Our subsequent report [5] detailed a population analysis of the PK data for the study that comprised a partial and complex cross-over design in these women. Plasma concentrations were described with a two-compartment model with differing PO and IM first-order absorption inputs. Overall, BET exhibited slower clearance, similar volume of distribution, faster absorption, and longer persistence than DEX. The BET-PA mixture exhibited rapid bioavailability of the phosphate form and extremely slow bioavailability of the acetate form with flip-flop kinetics.

This study also compared the time-course of several biomarkers including plasma cortisol, glucose, and cell trafficking of basophils, neutrophils, T-helper cells, and T-cytotoxic cells [4] in order to compare the PD of DEX and BET. While head-to-head comparison and modeling of the PK/PD of similar biomarker effects have been made for hydrocortisone, prednisolone, methylprednisolone, and dexamethasone in healthy subjects [6, 7], there is surprisingly little information regarding the in vivo PD of BET. In assessing ex vivo lymphocyte suppression, one study reported 15-fold greater activity for BET than DEX [8]. However, another showed very similar inhibition constant, *IC*_*50*_, values of about 10 nM for the two drugs using similar methodology [9]. The relative receptor affinity of BET was cited as 60% of that of DEX for the GC receptor in human lung [10]. One study showed greater activity of DEX in inducing apoptosis in murine lymphoid cells, but similar affinity (equilibrium dissociation constant, *K*_*d*_, of about 8 nM) for the cytosolic GC receptors [11]. Thus, there remains considerable uncertainty regarding comparative in vivo efficacy of BET versus DEX. Their comparative efficacy in treating women at risk of preterm delivery has not been assessed and the WHO-recommended regimens [3] are considered equivalent.

The pharmacodynamics of GC have many complexities necessitating insightful approaches to PK/PD modeling. The major complexity is the circadian rhythm in the baseline patterns. In case of endogenous GC, the circadian rhythm is regulated by the light-activated hypothalamic–pituitary–adrenal axis that controls secretion of cortisol by the adrenal cortex [12]. The GC have been shown to inhibit movement of lymphocytes from the extravascular pool to the blood pool [13]. Therefore, the circadian variations of cortisol propagate on the baselines of T-lymphocytes. The circadian rhythm in baseline neutrophils is attributed to the cytokine CXCL12 that controls the release of neutrophils from the bone marrow. CXCL12 undergoes circadian changes in expression stimulated by the sympathetic nervous system [14]. The light–dark cycle regulates glucose metabolism via the central clock. Substantial evidence exists for circadian rhythms in glucose tolerance [15]. However, food intake obscures direct detection of the circadian rhythm in the baseline glucose plasma concentrations. The modeling of GC effects on biomarkers in blood can usually be handled with various indirect response models, while their tissue effects require multi-step PK/receptor/gene/protein systems models [10, 16, 17]. As the exogenously-dosed GC interfere with endogenous cortisol (corticosterone in rodents) circadian rhythms, appropriate PK/PD modeling often requires joint assessment of the adrenal suppression as well as the more direct action of the dosed steroid [13].

This report provides a population analysis of PK/PD data for dexamethasone phosphate (DEX-P) and betamethasone phosphate (BET-P) given PO and IM and as a betamethasone phosphate/acetate IM mixture (BET-PA) in a partial and complex cross-over design in 48 healthy nonpregnant Indian women. This analysis provides further insights into the comparative PK/PD properties of these important therapeutic agents. Our key objective is to demonstrate the application of various indirect response models that jointly assess adrenal suppression as well as either inhibitory or stimulatory effects of these steroids on cortisol, glucose, and cell trafficking responses in the studied women. The array of relative activities of BET versus DEX as assessed by several mechanism-based PKPD models is described.

## Methods

### Study design

The study design was described previously [4]. It was an open-label, randomized, two-period study in healthy female subjects under fasting conditions. The subjects (N = 48) were randomized into eight sequences of 6 subjects who received two treatments during two periods separated by a washout time. The first period started with overnight fasting, followed by 24-h blood sampling for baseline biomarker measurements, the drug administration at 7 AM, and subsequent blood draws up to 96 h. The subsequent washout interval was 10 days. The study protocol was approved by the ACE Independent Ethics Committee, Bangalore India, and by the Institutional Review Board at Cincinnati Children’s Hospital Medical Center.

### Subjects

The female subjects in the study were of ages 22–39 years with normal body mass index 20.6–25.0 kg/m^2^. The ranges for height were 144–167 cm and weight 47.0–68.7 kg. All subjects were of Indian ethnicity and were studied in India. The study inclusion criteria ensured that the women were healthy, non-smokers or moderate smokers, non-drinkers or occasional drinkers, not pregnant and using contraceptives. Three subjects dropped out of the study after completion of the first period. Their data for the first period were included in the analysis. All subjects consented to participate in the study. Further details are described in ClinicalTrials.gov NCT03668860.

### Food intake

For Period I subjects fasted 10 h before first baseline blood sampling at −24 h (7 AM) and for 4 h after. During the baseline (pre-dose) blood sampling lunch, snack, and dinner were served at −20 h (11 AM), −16 h (3 PM), and −12 h (7 PM). During the first day post-dose lunch (11 AM), snack (3 PM), and dinner (7 PM) were provided at 4, 8, and 12 h. On the following days breakfast (7 AM), lunch, snack, and dinner were served at 24, 28, 32, 36, 48, 52, 56, 60, 72, 76, 80, and 84 h. For Period II subjects fasted 10 h before a dose at time 336 h (7 AM) and 4 h after. The food intake clock times were identical with ones for Period I. The mealtimes after the first dose were shifted by 336 h when modeling the second dose data.

### Drug administration

All subjects were given single doses of 6 mg of either DEX or BET in each period. The IM DEX-P (Treatment A) was dexamethasone phosphate solution (Fresenius Kabi USA LLC). The IM BET-P (B) was betamethasone phosphate solution (BETENESOL®, Glaxo SmithKline Pharmaceuticals Ltd, India). The second IM BET-PA injection (C) was betamethasone phosphate (3 mg) and acetate (3 mg) suspension (Celestone®, Merck and Co, Inc, USA). The PO DEX-P (D) was dexamethasone phosphate tablets (Cadila Healthcare Ltd, India). The PO BET-P (E) was betamethasone phosphate tablets (BETNESOL®, Glaxo SmithKline Pharmaceuticals Ltd, India). The cross-over sequences were AB, BA, CD, DC, ED, DE, CE, and EC. Drug contents in the dosage forms were pre-checked using LC–MS/MS. The doses listed for PK/PD were the free alcohol equivalents in the formulations.

### Blood sampling

For Period I and following an overnight fast, the 24-h baseline blood samples were drawn beginning at 7:00 AM at 0, 1, 2, 3, 4, 6, 9, 11, 15, and 24 h. For this and the cross-over phase, blood samples for PK and PD measurements were drawn at 0 (pre-dose), 0.5, 1, 1.5, 2, 3, 4, 6, 12, 18, 24, 30, 36, 48, 60, 72, and 96 h after drug dosing. Anticoagulant K2EDTA was added to blood samples. Plasma was separated from whole blood by centrifugation at 4 °C within 30 min after withdrawal. Plasma samples were stored at −70 °C before further cortisol and drug analysis.

### Bioanalytical methods

Cortisol concentrations in plasma were quantified using validated LC–MS/MS methodology with a deuterated internal standard at Syngene Bioanalytical Research Laboratory (Syngene International Ltd, Bangalore, India). The lower limit of quantitation (LOQ) was 1.0 ng/mL [5]. Plasma glucose was measured by the glucose oxidase method. Blood neutrophils and basophils were counted with a SY5MEX XN 1000 hematology analyzer. The T-helper and T-cytotoxic cells were measured by automatic flow cytometry with a Beckman Coulter Navioz flow cytometer using CYTO-STAT tetra CHROME CD45-FITC/CD4-RD1/CD8-ECD/CD3-PC5 and CYTO-STAT tetra CHROME monoclonal antibody reagents.

### Parameter estimation

The individual subject plasma concentrations were utilized based on our population PK model published recently [5] using estimates of individual PK parameters that were calculated from the data set. Only PD biomarkers were fitted using the present models. Model parameters were estimated by maximizing the likelihood of observations using the Laplace with Interaction method implemented in NONMEM 7.4 (ICON Clinical Research LLC, North Wales, PA), Evaluation of model performance were done by assessing changes in objective function values, standard errors of parameter estimates, goodness-of-fit plots, and visual predictive checks. The plots were generated by R 4.0.0 packages (ggplot2, lattice, vpc) [18] using RStudio 1.1.383 [19].

## Results

### Pharmacokinetics

**Fig. 1:**
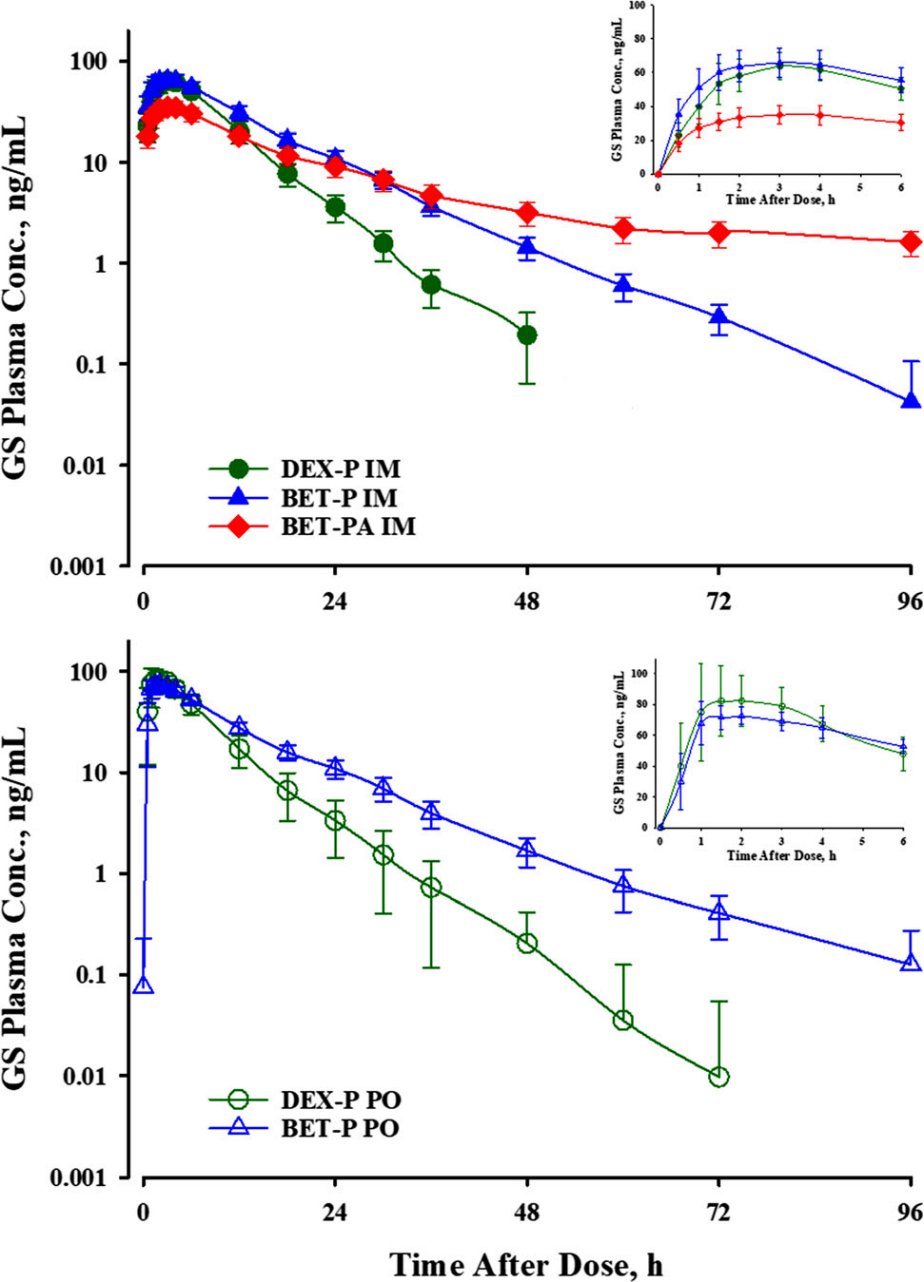
Mean plasma concentrations of DEX and BET in study subjects following administration of a single dose 6 mg intramuscularly (upper panel) and orally (lower panel). Bars indicate standard deviations. The inset shows plasma concentrations during the first 6 h

Figure 1 compares the mean plasma concentrations over time of DEX and BET after the PO and IM dosing of the five studied formulations [4]. The early absorption phases showed fairly rapid and smooth up-curves with rounded peaks. Interestingly, both DEX and BET peaked earlier after PO rather than IM dosing, but both usually between 2 and 3 h. All curves showed at least two exponential decline phases with the DEX terminal half-life, *t*_***1/2***_, of about 7.5 h and BET *t*_***1/2***_ of about 17 h. The IM BET-PA profiles exhibited a prolonged terminal phase with a 78 h half-life owing to the slow hydrolysis/absorption from the acetate form. These properties supported the selection of a two-compartment model with differing first-order absorption rates depending on the drug and route of administration. The details of the population modeling of these data are available [5]. The PK parameters for each drug, formulation, and subject were used in population modeling of the biomarkers.

### Cortisol pharmacodynamics

Endogenous cortisol is used as a biomarker for the hypothalamic–pituitary–adrenal (HPA) suppression. Secretion of cortisol by the adrenal cortex is controlled by adrenocorticotropin hormone (ACTH) produced by the anterior pituitary gland. Release of ACTH in turn is controlled by corticotropin-releasing factor (CRF). Corticosteroids negatively feedback on the hypothalamus to decrease the formation of CRF and the anterior pituitary to decrease the formation of ACTH resulting in suppression of endogenous cortisol secretion. The light-activated central clock controls the activity of the HPA axis through synapses between the suprachiasmatic nuclei of the hypothalamus and the neurons located in the paraventricular nuclei which secrete CRF [12]. This mechanism serves as the basis for the circadian release of GC, which in humans usually reach their peak concentrations early in the morning and their nadir concentrations around midnight.

**Fig. 2:**
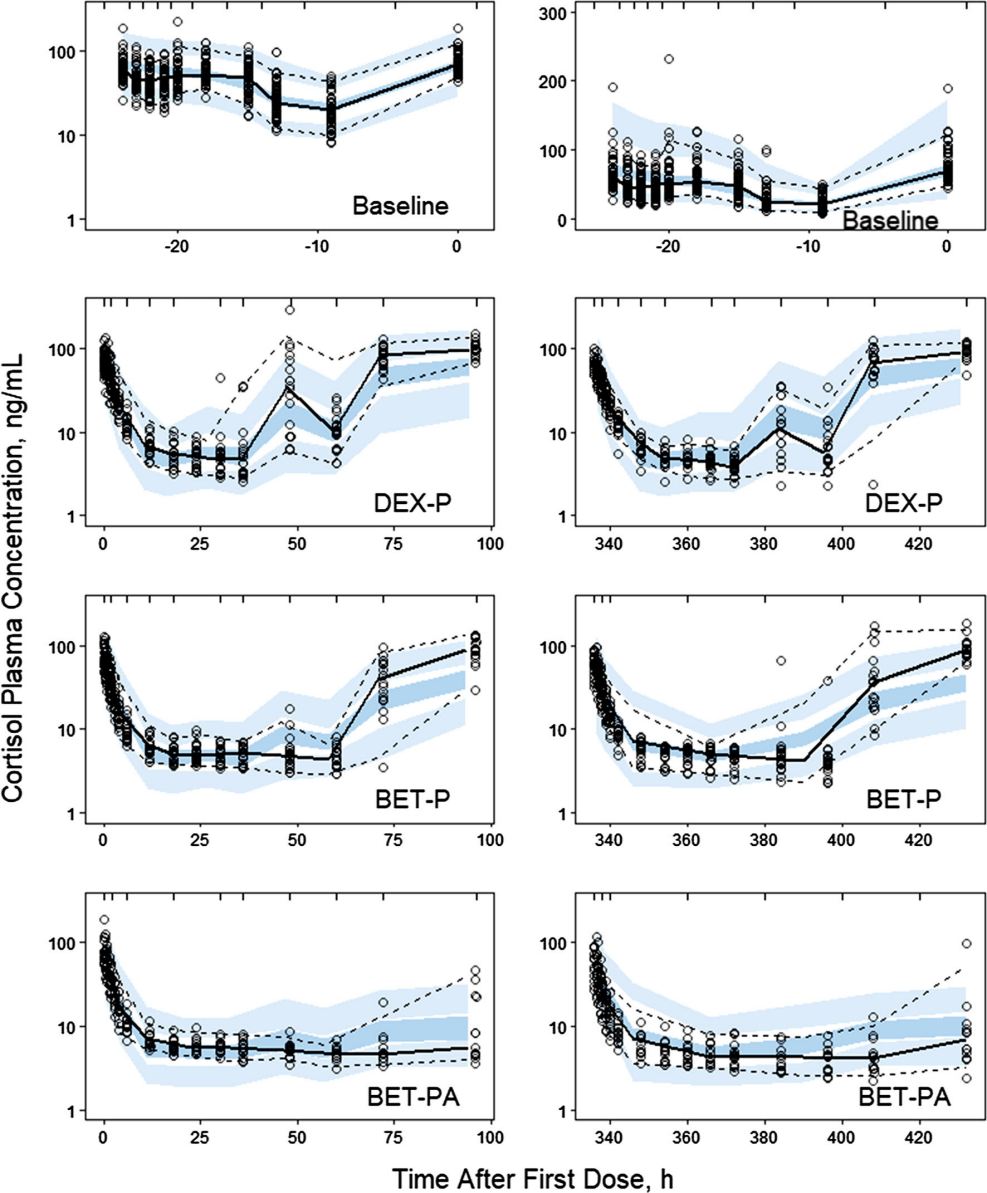
Visual predictive check plots for cortisol plasma concentrations following administration of a single IM and PO dose of dexamethasone phosphate (DEX-P), betamethasone phosphate (BET-P), and phosphate/acetate mixture (BET-PA). Symbols represent observed plasma concentrations, continuous line is the median, and dashed lines are 5^th^ and 95th percentiles of observed values. The shaded regions are model-predicted confidence intervals for these percentiles. The baselines are shown in both semi-log (left) and linear (right) scales. Other panels show results from Period I on the left and Period II on the right

**Fig. 3:**
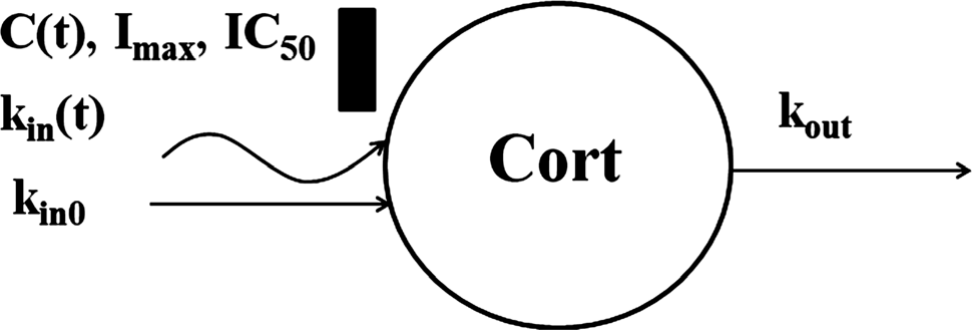
Model for plasma cortisol (*Cort*) produced by the adrenal gland that is subject to the circadian rhythm (*k*_*in*_*(t)*) and by another source at a constant rate *k*_*in0*_. Cortisol is eliminated from plasma at a first- order rate. Adrenal gland production is inhibited by corticosteroid plasma concentrations (*C(t)*). The inhibition follows the *I*_*max*_ model. Both *I*_*max*_ and *IC*_*50*_ are drug specific (DEX, BET). Symbols are further defined in Table 1

The pre-dose cortisol data shown in Fig. 2 exhibit a strong asymmetric circadian pattern with median peak time -24 h (7 AM), *Cort*_*max*_ = 88.9 ± 30.8 ng/mL and median nadir time -9 h (10 PM), *Cort*_*min*_ = 18.0 ± 7.1 ng/mL. We tested one, two and three harmonic models to describe the cortisol baseline [20] and selected the two-harmonic model based on performance, precision of parameter estimates, and parsimony. Administration of DEX and BET resulted in a prolonged suppression of cortisol with similar nadirs for all GC in the range of 2.5–2.9 ng/mL. The inhibitory effect of corticosteroids on cortisol secretion was described by an indirect response model [21]. The model diagram is shown in Fig. 3. Despite the large doses, we did not observe complete suppression of cortisol, although the oscillations were abolished during the nadir. Therefore, we included a constant production rate of cortisol that was not inhibited by GC (*k*_*in0*_):

1 $$ \frac{dCort}{{dt}} = k_{in} (t)\left( {1 - \frac{{I_{\max } C(t)}}{{IC_{50} + C(t)}}} \right) + k_{in0} - k_{out} Cort $$

where *C(t)* denotes GC plasma concentration, *k*_*in*_*(t)* is the circadian cortisol production, and *k*_*out*_ is the first-order elimination rate constant. The *I*_*max*_ and *IC*_*50*_ are GC-specific maximal inhibition and plasma concentration eliciting 50% of the maximal inhibition. We set *I*_*max*_ = 1.0 both for DEX and BET. To describe the baseline cortisol, we selected two harmonics of periods *T* = 24 h and *T/2* = 12 h:

2 $$ Cort_{baseline} (t) = Cort_{0} + a_{0} + a_{1} \cos \left( {\frac{2\pi }{T}t} \right) + b_{1} \sin \left( {\frac{2\pi }{T}t} \right) + a_{2} \cos \left( {\frac{4\pi }{T}t} \right) + b_{2} \sin \left( {\frac{4\pi }{T}t} \right) $$

The *Cort*_*0*_ represents the baseline attributed to *k*_*in0*_:

3 $$ k_{in0} = k_{out} Cort_{0} $$

The circadian cortisol production was calculated from Eq. (1) *C(t)* = 0:

4 $$ k_{in} \left( t \right) = \frac{{dCort_{baseline} }}{dt} + k_{out} Cort_{baseline} - k_{in0} $$

Since the time *t* = 0 was set for the first dose administration, the initial condition for pre-dose baseline was *t* = *-T* =−24 h:

5 $$ Cort\left( { - 24} \right) = Cort_{baseline} \left( { - T} \right) = Cort_{0} + a_{0} + a_{1} + a_{2} $$

Equation (4) does not imply the positive sign of *k*_*in*_*(t)* if *Cort*_*baseline*_(*t*) > 0. Therefore, we imposed constraints on the baseline parameters to ensure *k*_*in*_*(t)* ≥ 0 (see Appendix 1). All parameters were assumed to be log-normally distributed among subjects:

6 $$ P = \theta_{P} \exp \left( {\eta_{P} } \right)\;{\text{and}}\;\eta_{P} \sim {\mathcal{N}}\left( {0,\omega_{P}^{2} } \right) $$

where ${\theta }_{P}$ is a typical value of parameter $P$, ${\eta }_{P}$ is the deviation from ${\theta }_{P}$, and ${\omega }_{P}^{2}$ is the variance of ${\eta }_{P}$. The observed cortisol plasma concentrations *Cort*_*ij*_ were log-transformed and the constant residual error model was applied:

7 $$ \log Cort_{ij} = \log Cort\left( {t_{ij} } \right) + \varepsilon_{ij} \,{\text{and}} \, \varepsilon_{ij} \sim {\mathcal{N}}\left( {0,\sigma^{2} } \right) $$

Here the index *ij* denotes *j*^*th*^ observation for *i*^*th*^ subject, and *t*_*ij*_ is the sampling time. All residual errors *ɛ*_*ij*_ are assumed to be independent.

The Pre-dose, DEX-P, BET-P, and BET-PA inhibited cortisol data were fitted simultaneously. Parameter estimates are shown in Table 1. The estimate of the baseline parameter *A*_*0*_ was close to zero and finally set at 0. We were unable to estimate variances of IIV parameters for *a*_*2*_ and *k*_*out*_ with reasonable precision and ultimately set them to 0. Significant between-occasion variability (BOV) for *Cort* baseline parameters was not detected. The relative standard errors (RSE) of estimates of fixed effect parameters were less or equal to 22% with the exception of ${\theta }_{a2}$. The RSE for estimates of random effect parameters were higher but less than 53% with the exception of ${\omega }_{b1}^{2}$. This implies that we did not overparameterize the model and the observed data allowed us to resolve model parameters with acceptable precision.

**Table 1:** Estimates and relative standard errors (%RSE) of parameters for the population PD model of cortisol, Eqs. (1)-(7)

Parameter, units	Definition	Typical value (%RSE)	Variance of IIV (%RSE)
*A*_*0*_, ng/L	Constraint on the mean cortisol baseline *a*_*0*_ ** that enforces the non-negative sign of the cortisol production rate	0*	NA
*a*_1_, ng/L	Cosine coefficient of 24 h harmonic for cortisol baseline	23.6 (4.2)	0.0686 (28.3) (26.2)***
*b*_1_, ng/L	Sine coefficient of 24 h harmonic for cortisol baseline	1.70 (54.0)	1.03 (73.2) (101)***
*a*_2_, ng/L	Cosine coefficient of 12 h harmonic for cortisol baseline	-2.51 (21.7)	0*
*b*_2_, ng/L	Sine coefficient of 12 h harmonic for cortisol baseline	-7.30 (11.5)	0.329 (26.7) (57.4)***
*k*_*out*_, 1/h	First-order rate constant for elimination from plasma	0.378 (3.1)	0*
*IC*_*50DEX*_, ng/L	*IC*_*50*_ for DEX inhibitory effect	0.0549 (22.0)	0.892 (52.6) (94.4)***
*IC*_*50BET*_, ng/L	*IC*_*50*_ for BET inhibitory effect	0.153 (10.2)	0.297 (34.7) (54.5)***
*Cort*_0_, ng/L	Cortisol baseline not suppressed by DEX or BET	3.93 (3.9)	0.0616 (25.6) (24.8)***
σ^2^	Variance of residual error	NA	0.142 (6.6)

*Parameter was fixed

**${a}_{0}={A}_{0}+\frac{{R}_{1}+{R}_{2}}{{k}_{out}}$ and ${R}_{1}$ and ${R}_{2}$ are the amplitudes of 24 and 12 h harmonics

***Variance of log-normally distributed parameter expressed as %CV

The circadian oscillations of the baseline cortisol concentration were well captured by the two harmonics model with the exception of some early times as seen in Fig. 2 that may relate to stress. The short lasting 3–4 h nadir could have been better captured by a third harmonic, but at the expense of two more model parameters that were poorly estimated. The drug-altered cortisol data were well captured during the onset and nadir of drug effects as shown in Fig. 2. For DEX-P and BET-P the model slightly under-predicted the return phase of cortisol to the baseline allowing for a slower recovery than actually observed. For BET-PA the return phase was over-predicted. This could be partially explained by much higher variability in the data in the recovery phase compared to the phases when drug effects were present. The observed vs. predicted diagnostic plots did not show signs of systematic bias (Fig. S1). We did not observe differences in model predictions between the two periods. The overall good agreement between model predictions and observations was confirmed by individual subject cortisol vs. time plots shown in supplementary Figs. S2–S4.

**Fig. 4:**
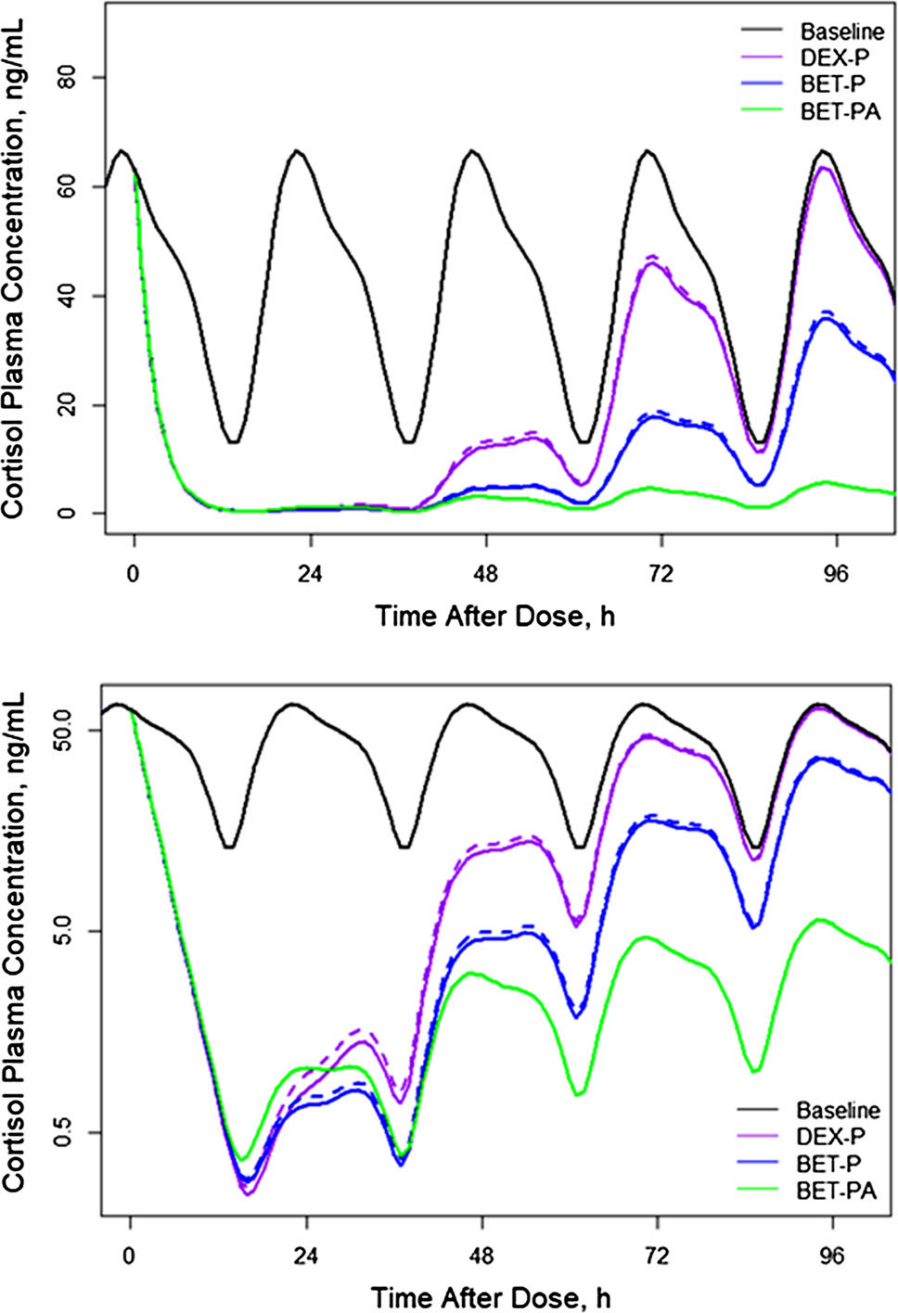
Simulated expected cortisol plasma concentrations in linear (upper panel) and logarithmic (lower panel) scales versus time following administration of 6 mg of indicated corticosteroid. Continuous lines depict IM and dashed lines PO routes

Figure 4 shows simulations of expected cortisol plasma concentrations following IM and PO doses of 6 mg for DEX-P, BET-P, and BET-PA. All drugs strongly suppress cortisol that reaches its nadir at about 15–16 h. The mean nadir concentrations are 0.25 for DEX-P, 0.28 for BET-P, and 0.36 ng/mL for BET-PA. The nadir phase lasts up to 37 h for all drugs after which time the cortisol response starts returning to the baseline. Only the response to DEX-P reaches the baseline before 96 h. The recovery phase for BET-P is longer than for DEX-P, but much faster than for BET-PA. The differences in cortisol responses between IM and PO dosing are minimal.

### Basophil pharmacodynamics

Basophils are the least abundant granulocytes as they account for approximately 0.5% of circulating leukocytes and approximately 0.3% of nucleated marrow cells [22]. They differentiate and mature in the bone marrow and then circulate in the blood. Basophil lifespans under homeostatic conditions are about 1–2 days [23]. Basophils are effector cells responsible for inflammatory reactions during immune responses. In response to inflammatory signals, they rapidly expand in the bone marrow, are mobilized to the blood, and are recruited into peripheral tissues at sites requiring immunogenic responses. Basophils are used as markers of GC suppression of inflammatory responses.

**Fig. 5:**
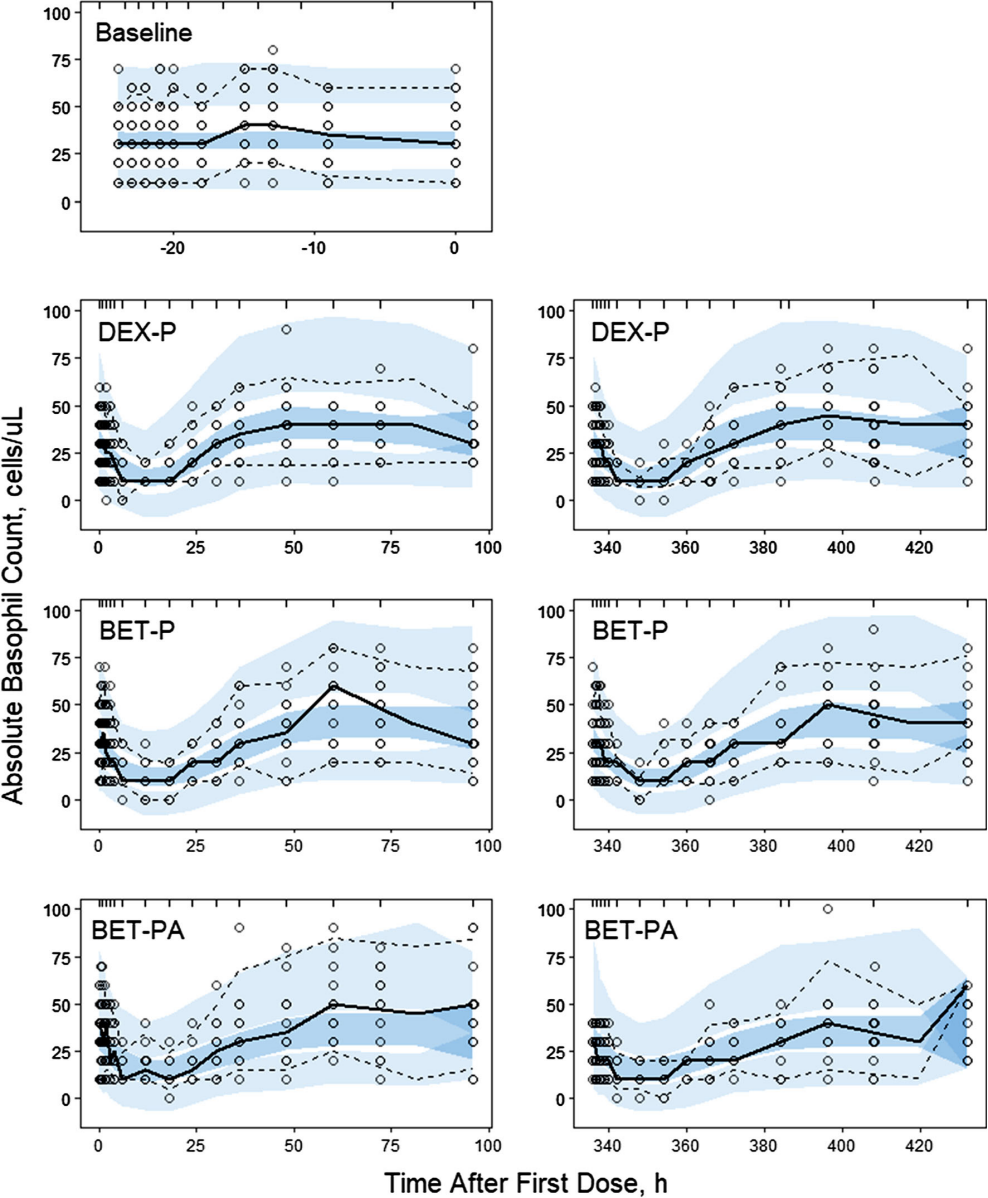
Visual predictive check plots for basophils following administration of a single IM and PO dose of DEX-P, BET-P, and BET-PA. Graphical features are the same as in Fig. 2

The blood basophil profiles are shown in Fig. 5. We did not detect circadian variations in the baseline data as observed individual average values ranged 13–61 cells/μL. Therefore, the basophil production rate was assumed to be constant. The GC suppress basophil counts that reach a nadir of 7–11 cells/μL at about 5–12 h after dosing. Following the nadir, basophils start returning towards the baseline and continue to increase resulting in a rebound with a peak of 45–55 cells/μL around 33–60 h after dosing. This behavior is characteristic of the temporal GC blockage and later release of basophils from the bone marrow. Consequently, we selected an indirect response model with a precursor compartment to describe basophil counts in blood [24]:

8 $$ \frac{dP}{{dt}} = k_{inP} - k_{p} \left( {1 - \frac{{I_{\max BASO} C\left( t \right)}}{{IC_{50BASO} + C\left( t \right)}}} \right)P $$

9 $$ \frac{dBASO}{{dt}} = k_{p} \left( {1 - \frac{{I_{\max BASO} C\left( t \right)}}{{IC_{50BASO} + C\left( t \right)}}} \right)P - k_{outBASO} BASO $$

with the baseline initial conditions:

10 $$ P\left( 0 \right) = \frac{{k_{outBASO} }}{{k_{p} }}BASO_{0} \;{\text{and }}BASO\left( 0 \right) = BASO_{0} $$

**Fig. 6:**
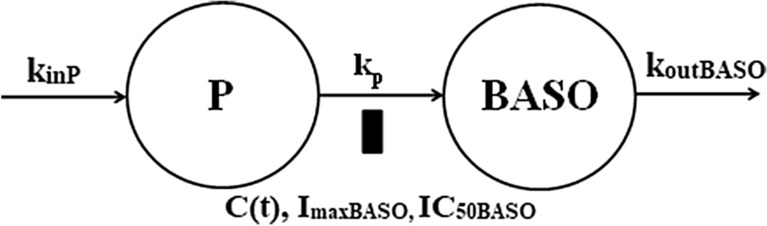
Basophil model where cells are released to the circulation from a precursor pool at the first-order rate *k*_*p*_ and exit the circulation at the first-order rate *k*_*outBASO*_. The precursor pool for basophils is replenished at the zero-order rate *k*_*inP*_. Corticosteroids inhibit the transfer of basophils from the precursor to the circulation. The inhibition (*I*_*maxBASO*_, *IC*_*50BASO*_) will result in a temporal decrease of BASO followed by a rebound. Symbols are further defined in Table 2

The model diagram is shown in Fig. 6. Basophils (*BASO*) are released to the blood from a precursor pool (*P*) with the first-order rate constant *k*_*p*_ and exit the circulation with the first-order rate constant *k*_*outBASO*_. The precursor pool for basophils is replenished at the zero-order rate *k*_*inP*_:

11 $$ k_{inP} = k_{outBASO} BASO_{0} $$

where *BASO*_*0*_ denotes the basophil baseline. The *I*_*maxBASO*_ and *IC*_*50BASO*_ are GC-specific maximal inhibition and plasma concentration eliciting 50% of the maximal inhibition. We set *I*_*maxBASO*_ = 1.0 in view of the almost complete inhibition for most subjects. All parameters were assumed to be log-normally distributed among subjects according to Eq. (6). Due to low abundance, the absolute basophil counts were measured in increments of 10 cells/μL with 0 cells considered as an observation. This would constitute basophils as categorical data. However, given the range of 0–130 cells/μL, basophils were considered as continuous data [25]. The constant residual error model was applied to describe the observed basophil counts:

12 $$ BASO_{ij} = BASO\left( {t_{ij} } \right) + \varepsilon_{ij} \;{\text{and}} \, \varepsilon_{ij} \sim {\mathcal{N}}\left( {0,\sigma_{BASO}^{2} } \right) $$

Here the index *ij* denotes *j*^*th*^ observation for *i*^*th*^ subject, and *t*_*ij*_ is the sampling time. All residual errors *ɛ*_*ij*_ are assumed to be independent.

The Pre-dose, DEX-P, BET-P, and BET-PA altered basophil data were fitted simultaneously. Parameter estimates are listed in Table 2. We did not detect a significant BOV for *BASO* baseline parameters. The relative standard errors of estimates of variances of IIV for *k*_*p*_ and *k*_*out*_ were large (> 100%) and consequently we set those variances to 0. The RSE of estimates of the remaining fixed and random effect parameters were less than 39%. This implies that the model parameters were estimated with acceptable precision.

**Table 2:** Estimates and relative standard errors (%RSE) of parameters for the population PD model of basophils, Eqs. (8)-(12)

Parameter, units	Definition	Typical value (%RSE)	Variance of IIV (%RSE)
*BASO*_0_, cells/μL	Baseline basophil count	31.4 (6.3)	0.148 (18.9) (38.5)**
*k*_p_, 1/h	First-order release rate constant	0.0463 (20.7)	0*
*k*_*outBASO*_, 1/h	First-order removal rate constant	0.137 (7.2)	0*
*IC*_*50BASODEX*_, ng/L	*IC*_*50*_ for DEX inhibitory effect	4.34 (25.3)	0.820 (38.7) (90.6)**
*IC*_*50BASOBET*_, ng/L	*IC*_*50*_ for BET inhibitory effect	6.66 (22.1)	0.373 (35.4) (61.1)**
*I*_*maxBASODEX*_	*I*_*max*_ for DEX inhibitory effect	1.0*	NA
*IC*_*maxBASOBET*_	*I*_*max*_ for BET inhibitory effect	1.0*	NA
σ_BASO_^2^, (cells/μL)^2^	Variance of residual error	NA	71.9 (7.6)

*Parameter was fixed

**Variance of log-normally distributed parameter expressed as %CV

The median observed basophil counts were well-captured by the model as seen in Fig. 5. The nadirs for all drug responses were within the 95% confidence regions for predictions as were most of the rebound peaks. The BET-P and BET-PA median basophil rebounds were slightly underpredicted by the model. The negative basophil count values for the 95% confidence region for the 5th percentiles of observations are the consequence of the constant residual error added to near 0 model predictions. The observed vs. predicted diagnostic plots did not show signs of systematic bias (Fig. 5S). We did not observe a difference in model predictions between the two periods. The overall good agreement between model predictions and observations was confirmed by individual subject basophil vs. time plots shown in supplementary Figs. S6–S8.

**Fig. 7:**
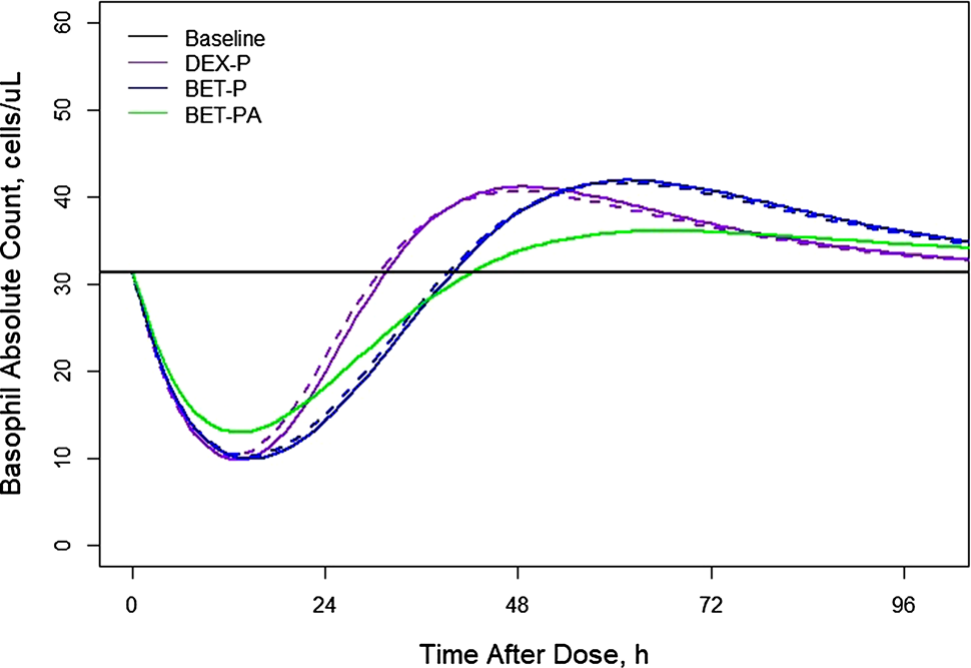
Simulated expected basophil absolute counts following administration of 6 mg of indicated corticosteroid. Continuous lines depict IM and dashed lines PO routes

Figure 7 shows simulations of typical basophil counts following the 6 mg IM and PO doses of DEX-P, BET-P, and BET-PA. The IM responses start at the baseline of 31.4 cells/μL and reach a nadir of 10 cells/μL for DEX and BET at about the same time at 13–15 h after which they rebound to reach a peak of 41 cells/μL (DEX), 42 cells/μL (BET), and 36 cells/μL (BET-PA) at times 49, 62, and 66 h. The PO dose responses are almost identical to IM ones. At 96 h, all responses almost returned to the baseline.

### Neutrophil pharmacodynamics

Neutrophils are the most abundant granulocytes in the peripheral blood ranging 54% to 62% of the circulating leukocytes. Neutrophils are produced from stem cells in the bone marrow where they spent 1 to 2 weeks maturing to the segmented granulocytes. Upon release to the peripheral blood, half of the neutrophils circulate and half marginalize. They spend about 10 h in the blood before margination and then extravasate to the extravascular tissues where they have a lifespan of a few days [26]. Neutrophils undergo a significant diurnal variation with a peak in the absolute count in the afternoon and a nadir in the morning. The circadian production of neutrophils by the bone marrow is controlled by the cytokine CXCL12 that undergoes circadian changes in expression stimulated by the sympathetic nervous system [14]. Neutrophils function to migrate to areas of tissue damage or infection where they act as phagocytes. In response to an infection, the bone marrow releases an increased number of neutrophils. Absolute neutrophil counts (ANC) serve as a clinical marker of infection.

**Fig. 8:**
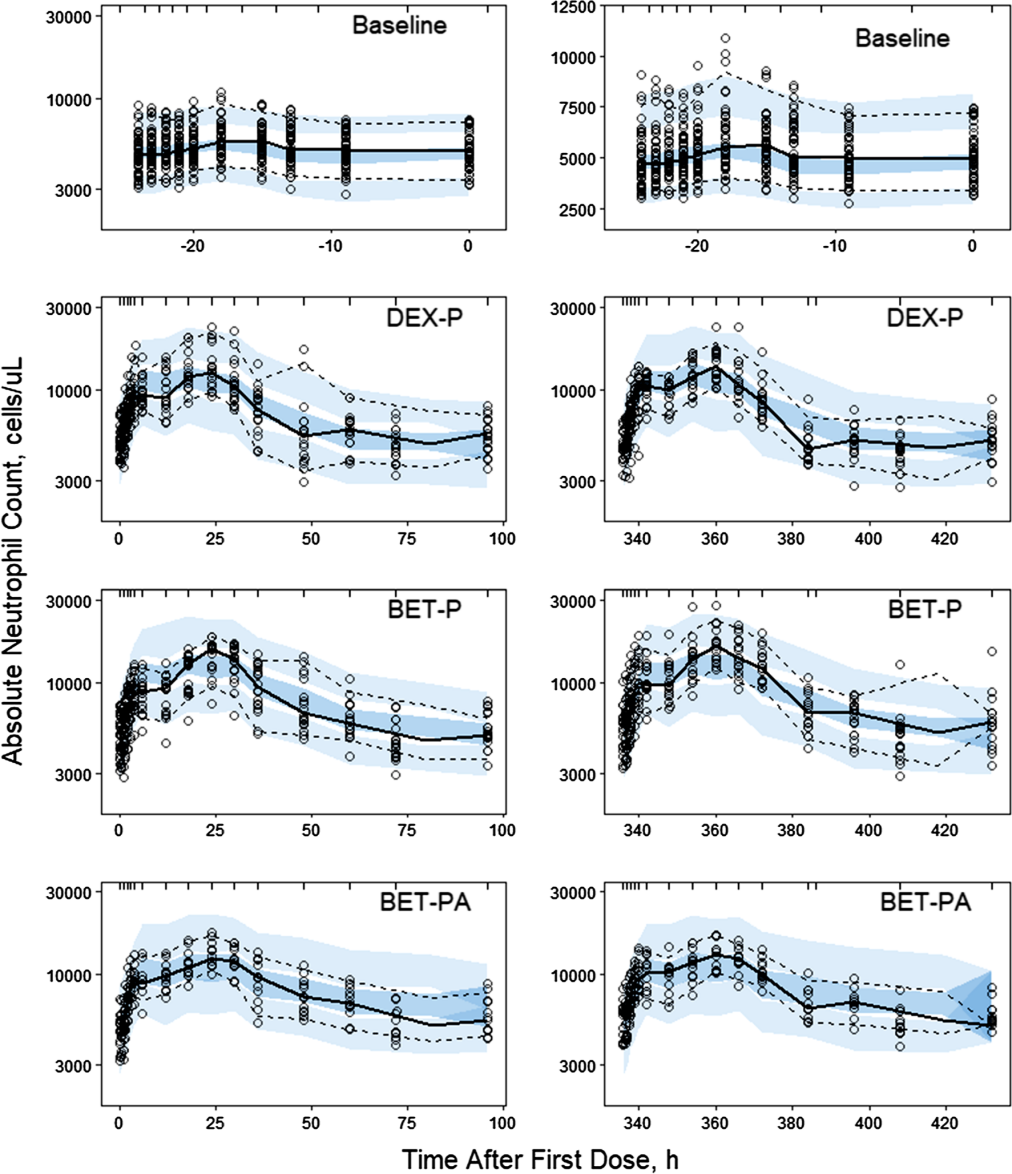
Visual predictive check plots for neutrophils following administration of single IM and PO doses of DEX-P, BET-P, and BET-PA. Graphical features are the same as in Fig. 2

**Fig. 9:**
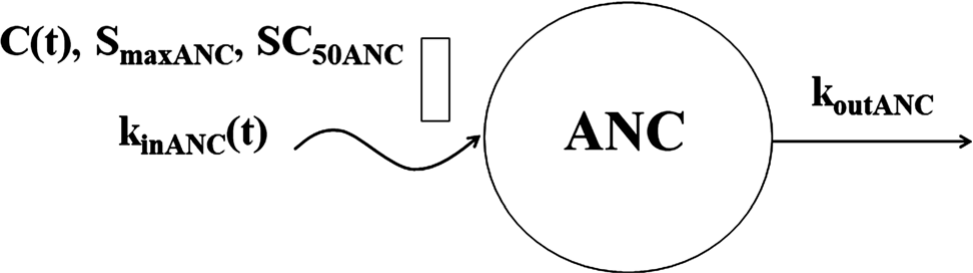
Neutrophil model where cells (ANC) are released from the bone marrow to the circulation at a rate that is subject to the circadian rhythm (*k*_*inANC*_*(t)*). The neutrophil production is stimulated by corticosteroids (*C(t)*). The stimulation obeys the *S*_*max*_ model. Both *S*_*maxANC*_ and *SC*_*50ANC*_ are drug-specific (DEX, BET). Neutrophils are removed from the blood at a first-order rate *k*_*outC*_. Symbols are further defined in Table 3

The pre-dose ANC data shown in Fig. 8 exhibit a moderate asymmetric circadian rhythm with median peak time -18 h (1 PM) *ANC*_*max*_ = 6341 ± 1490 cells/μL and median nadir time -22 h (9 AM), *ANC*_*min*_ = 4386 ± 1010 cells/μL. We selected the two-harmonic model to describe the ANC baseline [20]. Administration of DEX and BET resulted in a peak in the ANC response in the range of 11,511–15,169 cells/μL followed by a return to the baseline. The stimulatory effect of GC on ANC production by the bone marrow was described by a basic indirect response model [21]. The model diagram is shown in Fig. 9. The model equation is:

13 $$ \frac{dANC}{{dt}} = k_{inANC} \left( t \right)\left( {1 + \frac{{S_{\max ANC} C\left( t \right)}}{{SC_{50ANC} + C\left( t \right)}}} \right) - k_{outANC} ANC $$

where *C(t)* denotes the GC plasma concentration, *k*_*inANC*_*(t)* is the circadian *ANC* production, and *k*_*outANC*_ is the first-order removal rate constant. The *S*_*maxANC*_ and *SC*_*50ANC*_ are GC-specific maximal stimulation and plasma concentration eliciting 50% of maximal stimulation. The baseline *ANC* was described with two harmonics of periods *T* = 24 h and *T/2* = 12 h:

14 $$ ANC_{baseline} \left( t \right) = a_{0ANC} + a_{1ANC} \cos \left( {\frac{2\pi }{T}t} \right) + b_{1ANC} \sin \left( {\frac{2\pi }{T}t} \right) + a_{2ANC} \cos \left( {\frac{4\pi }{T}t} \right) + b_{2ANC} \sin \left( {\frac{4\pi }{T}t} \right) $$

The circadian *ANC* production was calculated from Eq. (1) *C(t)* = 0:

15 $$ k_{inANC} \left( t \right) = \frac{{dANC_{baseline} }}{dt} + k_{outANC} ANC_{baseline} $$

Since the time *t* = 0 was set as the first dose administration, the initial condition for pre-dose baseline was *t* = *-T* = -24 h:

16 $$ ANC\left( { - 24} \right) = ANC_{baseline} \left( { - T} \right) = a_{0ANC} + a_{1ANC} + a_{2ANC} $$

We did not implement constraints on the *ANC* baseline parameters to control the positive sign of *k*_*inANC*_*(t)*. All parameters were assumed to be log-normally distributed among subjects according to Eq. (6). As the observed *ANC* at *t* = -24, 0, and 336 h differed (4965 ± 1342, 5034 ± 1204, and 5300 ± 1419 cells/μL), between-occasion variability (BOV) of the baseline model parameters was included. These occasions included pre-treatment (*PER* = 0), Period I 0 ≤ *t* ≤ 96 h (*PER* = 1), and Period II 336 h ≤ *t* (*PER* = 2):

17 $$ P = \theta_{P} \exp \left( {\eta_{P} + \eta_{P,0} \left( {1 - PER} \right)\left( {2 - PER} \right)/2 + \eta_{P,1} PER\left( {2 - PER} \right) + \eta_{P,2} \left( {PER - 1} \right)PER/2 } \right) $$

where $$P\in \{{a}_{0ANC}, {a}_{1ANC}, {b}_{1ANC}, {a}_{2ANC}, {b}_{2ANC}\}$$ and

18 $$ \eta_{P,i} \sim {\mathcal{N}}\left( {0,\omega_{BOVP}^{2} } \right)\quad {\text{for}}\;i = 0,1,2 $$

The observed *ANC*_*ij*_ were log-transformed and the constant residual error model was applied Eq. (7).

The Pre-dose, DEX-P, BET-P, and BET-PA affected ANC data were fitted simultaneously. Parameter estimates are listed in Table 3. We were unable to estimate variance of IIV for *b*_*1ANC*_ with reasonable precision and ultimately set it to 0. Similarly, variances of BOV for *a*_*1ANC*_, *a*_*2ANC*_, and *b*_*2ANC*_ were set to 0. The RSE of fixed effects parameters did not exceed 29% with the exception of *b*_*2ANC*_ (%RSE 53.3%). The RSE for random effect parameters were in the range 9–68%. The 95% confidence region for median predictions captured almost all median observations as shown in Fig. 11. This also applies to the 5^th^ percentile of observations. However, the 95th percentile was overpredicted by the model for DEX-P and BET-P during the first 10 h after dosing. A post hoc check of individual *k*_*inANC*_*(t)* revealed no negative values. The observed vs. predicted diagnostic plots did not show signs of systematic bias (Fig. 9S). The overall good agreement between model predictions and observations was confirmed by individual *ANC* vs. time plots shown in supplementary Figs. S10–S12.

**Table 3:** Estimates and relative standard errors (%RSEs) of parameters for the population PD model of neutrophils, Eqs. (13)-(18)

Parameter, units	Definition	Typical value (%RSE)	Variance of IIV (%RSE)	Variance of BOV (%RSE)
${a}_{0ANC}$, cells/μL	Mean ANC baseline	4900 (3.1)	0.0277 (27.0) (16.6)**	0.134 (8.7) (36.6)**
${a}_{1ANC}$, cells/μL	Baseline harmonic coefficient	112 (28.3)	0.581 (50.8) (76.2)**	0*
${b}_{1ANC}$, cells/μL	Baseline harmonic coefficient	178 (21.1)	0*	0.539 (14.0) (73.4)**
${a}_{2ANC}$, cells/μL	Baseline harmonic coefficient	-237 (11.1)	0.135 (58.0) (37.7)**	0*
${b}_{2ANC}$, cells/μL	Baseline harmonic coefficient	-59.7 (53.3)	1.12 (51.0) (103)**	0*
${k}_{outANC}$, 1/h	Removal rate constant	0.0924 (3.7)	0.0316 (41.8) (17.8)**	NA
${SC}_{50ANCDEX}$, ng/L	*SC*_*50*_ for DEX stimulatory effect	4.92 (10)	0.156 (55.6) (39.5)**	NA
${SC}_{50ANCBET}$, ng/L	*SC*_*50*_ for BET stimulatory effect	13.5 (8.9)	0.114 (67.9) (37.8)**	NA
${S}_{maxANCDEX}$	*S*_*max*_ for DEX stimulatory effect	2.44 (6.6)	0.0454 (39.9) (21.3)**	NA
${S}_{maxANCBET}$	*S*_*max*_ for BET stimulatory effect	2.91 (6.3)	0.0439 (58.3) (21.0)**	NA
σ^2^	Variance of residual error	NA	0.0271 (6.6)	NA

*Parameter was fixed

**Variance of log-normally distributed parameter expressed as %CV

**Fig. 10:**
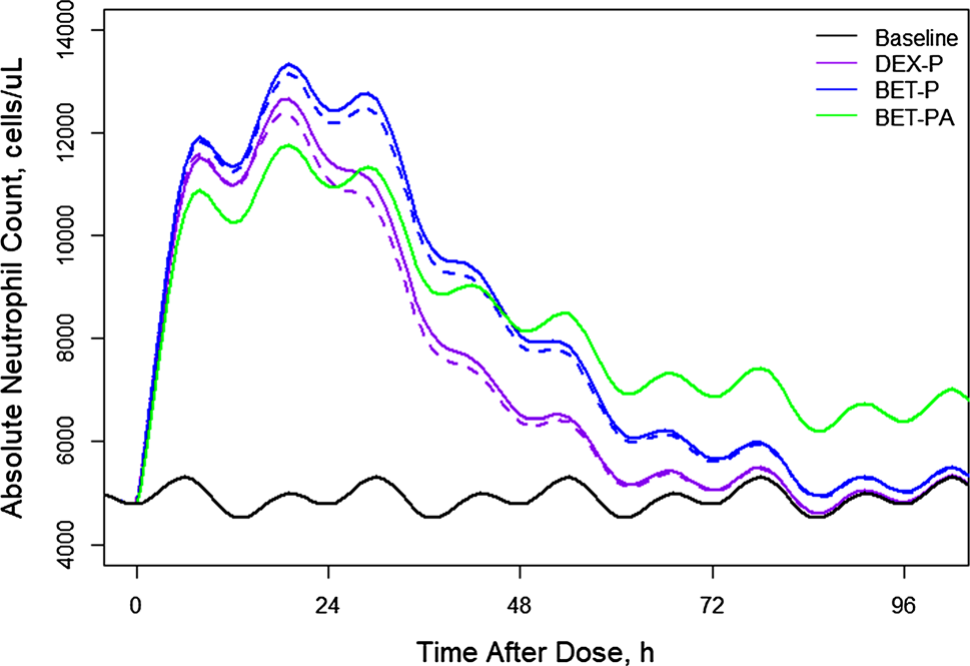
Simulated expected neutrophil absolute counts following administration of 6 mg of indicated corticosteroids. Continuous lines depict IM and dashed lines PO routes

Figure 10 shows simulations of typical *ANC* profiles following the 6 mg IM and PO doses of DEX-P, BET-P, and BET-PA. The mean of the *ANC* circadian baseline is 4900 cells/μL with the peak 5315 cells/μL at -18 h (1 PM) and nadir 4511 cells/μL at -11 h (8 PM). All *ANC* responses following DEX-P, BET-P, and BET-PA IM peak at 19 h after dosing and reach 12,677, 13,343, and 11,771 cells/μL. Subsequently, the *ANC* returns to the baseline at approximately 96 h (DEX-P), and 133 h (BET-P). The *ANC* response to BET-PA is remains elevated after 168 h. The maximum difference in *ANC* between DEX-P IM and PO is 416 cells/μL, and for BET-P IM and PO this difference is 283 cells/μL.

### T-helper cell pharmacodynamics

T-lymphocytes are a subpopulation of lymphocytes that originate from the bone marrow lymphoid precursor cells and migrate to the thymus where they proliferate and differentiate into mature cells. Mature T-lymphocytes in the thymus consist of T-helper and T-cytotoxic cells. Mature T-lymphocytes enter the circulation and subsequently migrate to extravascular tissues of the lymph nodes and spleen. The T-lymphocytes can re-enter the blood through lymphatic drainage. The circulation process between the intravascular and extravascular compartments is called cell trafficking. About 95% of the total body lymphocytes are located in the extravascular tissues and only 5% comprise the peripheral blood lymphocytes. The lifespan of most T-lymphocytes can vary from a few months to 20 years [27]. The normal range of lymphocytes in adult females is 1500–4000 cells/μL. Upon activation by contact with an antigen, T-lymphocytes proliferate into effector cells. These T-helper effector cells secrete various cytokines that activate immune response cells, whereas T-cytotoxic effector cells acquire the ability to recognize and eliminate antigen-altered self-cells. Absolute counts of T-helper lymphocytes (*TH*) and T-cytotoxic lymphocytes (*TC*) in the peripheral blood serve as markers of cell mediated immunity.

**Fig. 11:**
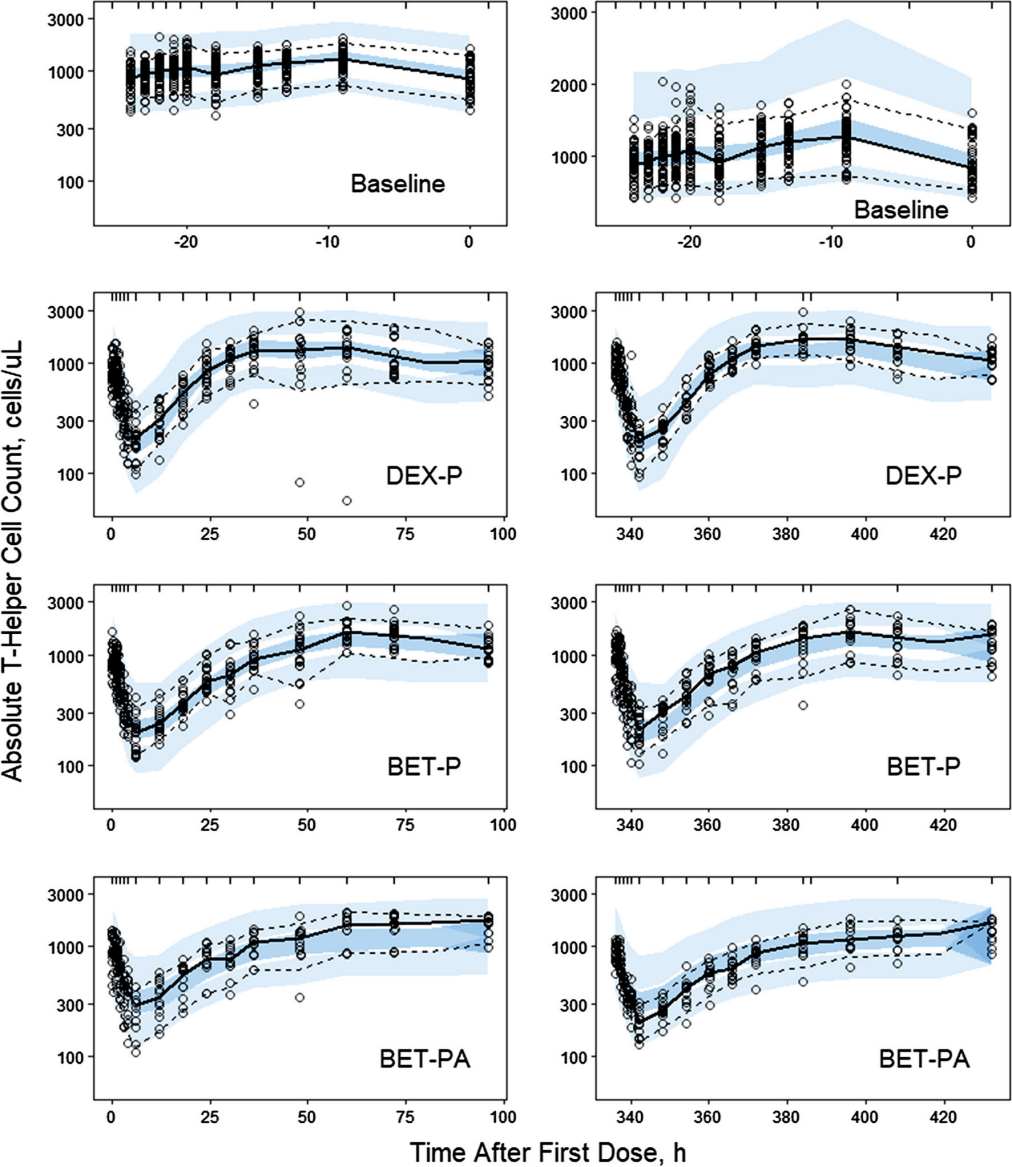
Visual predictive check plots for T-helper cells following administration of single IM and PO doses of DEX-P, BET-P, and BET-PA. Graphical features are the same as in Fig. 2

**Fig. 12:**
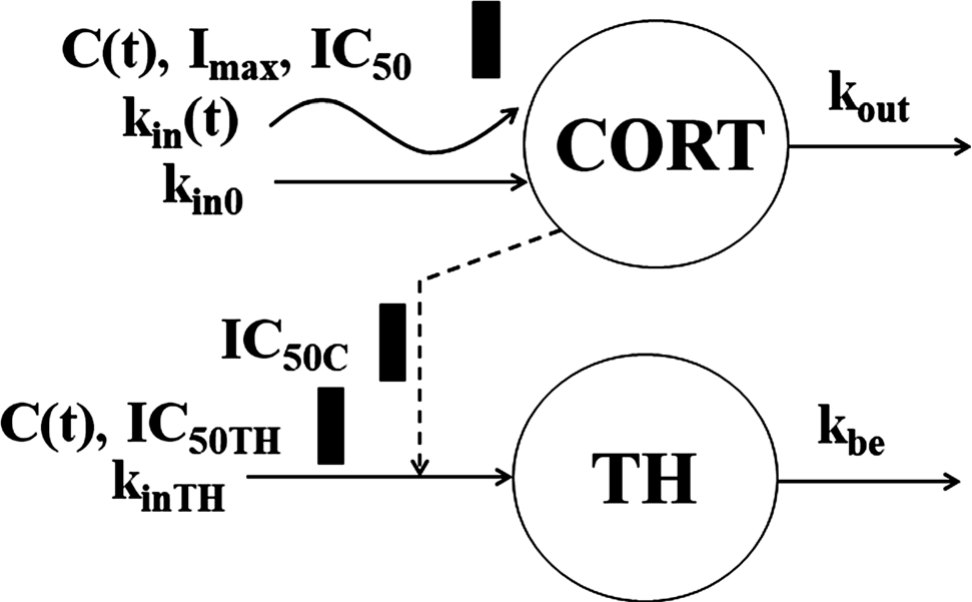
T-helper cell model where cells (*TH*) are released from extravascular tissues to the blood at the zero-order rate (*k*_*inTH*_) that is subjected to the circadian rhythm due to the cortisol (*CORT(t)*) inhibition (*IC*_*50C*_). The *TH* production is inhibited by corticosteroids (*C(t), IC*_*50TH*_). *TH* are removed from blood at the first-order rate *k*_*be*_. Symbols are further defined in Table 4

The GC have been shown to inhibit movement of lymphocytes from the extravascular pool to the blood pool [13]. Since endogenous cortisol exhibits circadian variations, these changes affect the T-lymphocyte trafficking producing a circadian rhythm in the *TH* baseline. The baseline *TH* data shown in Fig. 11 exhibit an asymmetric circadian rhythm with median peak time -13 h (6 PM), *TH*_*max*_ = 1342 ± 327 cells/μL, and median nadir time of -23 h (8 AM) with *TH*_*min*_ = 798 ± 205 cells/μL. Administration of DEX and BET resulted in a nadir in *TH* response in the range of 187–263 cells/μL followed by a return to the baseline. The inhibitory effect of joint GC on *TH* transit rates from the extravascular sites to the blood was described by the lymphocyte trafficking model introduced by Mager et al. [9]. The model diagram is shown in Fig. 12 and the operative equation is:

19 $$ \frac{dTH}{{dt}} = k_{inTH} \left( {1 - \frac{{C\left( t \right)\left( {IC_{50C} /IC_{50TH} } \right) + Cort\left( t \right)}}{{IC_{50C} + C\left( t \right)\left( {IC_{50C} /IC_{50TH} } \right) + Cort\left( t \right)}}} \right) - k_{be} TH $$

where *Cort(t)* is the cortisol plasma concentration described by Eq. (1), *C(t)* denotes GC plasma concentration, *k*_*inTH*_ is the zero-order rate constant of *TH* transfer from the extravascular pool to the blood, and *k*_*be*_ is the first-order transfer rate constant of *TH* from the blood to the extravascular pool. The *IC*_*50C*_ and *IC*_*50TH*_ reflect the cortisol and drug concentrations that each produce 50% inhibition of maximal lymphocyte influx. In the absence of exogenous GC, the *TH* baseline exhibits a *T* = 24 h rhythm described by:

20 $$ \frac{{dTH_{baseline} }}{dt} = k_{inTH} \left( {1 - \frac{{Cort_{baseline} \left( t \right)}}{{IC_{50C} + Cort_{baseline} \left( t \right)}}} \right) - k_{be} TH_{baseline} $$

with a unique initial condition determined by T-periodicity:

21 $$ TH_{baseline} \left( { - 24} \right) = TH_{baseline} \left( { - T} \right) = TH_{baseline} \left( 0 \right) $$

The initial condition for Eq. (19) is *TH(-24)* = *TH*_*baseline*_*(-24)*. Appendix 2 shows how to express *TH*_*baseline*_*(t)* as a sum of solutions to two differential equations with the zero initial conditions at -2* T* = -48 h. All parameters were assumed to be log-normally distributed among subjects according to Eq. (6). The observed *TH*_*ij*_ were log-transformed and the constant residual error model was applied Eq. (7).

The Pre-dose, DEX-P, BET-P, and BET-PA altered *TH* profiles were fitted simultaneously. Parameter estimates are listed in Table 4. We did not detect a significant BOV for *TH* baseline parameters. The RSE of fixed effect parameters did not exceed 11%. The RSE for random effect parameters were in the range 9–54% with the exception of IIV for *IC*_*50C*_ (112%). The 95% confidence region for median predictions captured almost all observations as shown in Fig. 11. This also applies to the 5^th^ percentile of observations. However, the 95th percentile was overpredicted by the model for the entire baseline and BET-PA during the first 48 h after the dose in the second period. The observed vs. predicted diagnostic plots did not show signs of systematic bias (Fig. S13). The overall good agreement between model predictions and observations was confirmed by individual *TH* vs. time plots shown in supplementary Figs. S14–S16.

**Table 4:** Estimates and relative standard errors (%RSE) of parameters for the population PD model of T-helper lymphocytes. Equations (19)-(21)

Parameter, units	Definition	Typical value (%RSE)	Variance of IIV (%RSE)
_*kinTH*_, cells/uL/h	Rate constant of $TH$ transfer from extravascular to blood pool	797 (5.6)	0.0736 (9.5) (25.6)*
*k*_*be*_, 1/h	Transfer rate constant from blood to extravascular pool	0.448 (2.6)	0.0198 (29.7) (14.1)*
*IC*_*50THDEX*_, ng/mL	*IC*_*50TH*_ for DEX inhibitory effect	4.23 (10.5)	0.106 (54.0) (32.6)*
*IC*_*50THBET*_, ng/mL	*IC*_*50TH*_ for BET inhibitory effect	5.24 (6.3)	0.0504 (34.9) (22.4)*
*IC*_*50THC*_, ng/mL	*IC*_*50TH*_ for cortisol inhibitory effect	78.4 (6.6)	0.0562 (112) (23.7)*
*σ*^*2*^	Variance of residual error	NA	0.0418 (18.0)

*Variance of log-normally distributed parameter expressed as %CV

**Fig. 13:**
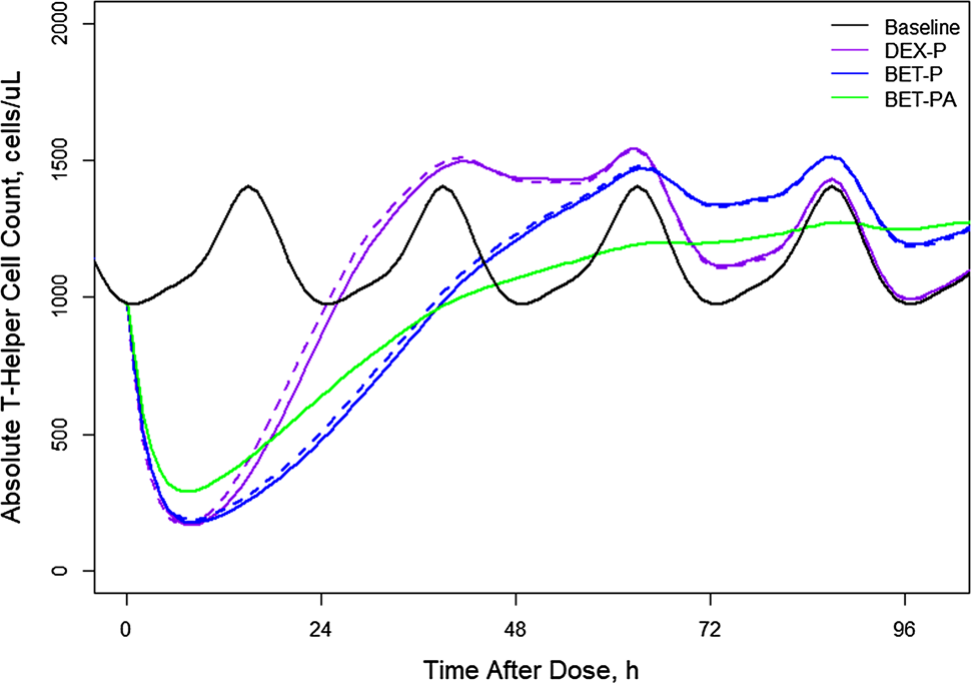
Simulated expected T-helper cell absolute counts following administration of 6 mg of indicated corticosteroids. Continuous lines depict IM and dashed lines PO routes

Figure 13 shows simulations of expected T-helper cell counts following IM and PO doses of 6 mg for DEX-P, BET-P, and BET-PA. The mean of the *TH* circadian baseline is 1140 cells/μL with the peak 1404 cells/μL and nadir 975 cells/μL occurring at -9 h (6 PM) and -23 h (8 AM). All *TH* responses following DEX-P, BET-P, and BET-PA IM dosing reach the nadir at 7–8 h of 167, 177, and 289 cells. Subsequently, *TH* returns to the baseline at approximately 27 h (DEX-P), 45 h (BET-P), and 46 h (BET-PA). The response continues rising to create a rebound that ends at about 96 h for DEX-P, and continues beyond 96 h for BET-P and BET-PA. During the suppression phase, *TH* responses do not show circadian oscillations. The differences in *TH* responses between IM and PO dosing is less than 18% for DEX-P and 8% for BET-P.

### T-cytotoxic cell pharmacodynamics

**Fig. 14:**
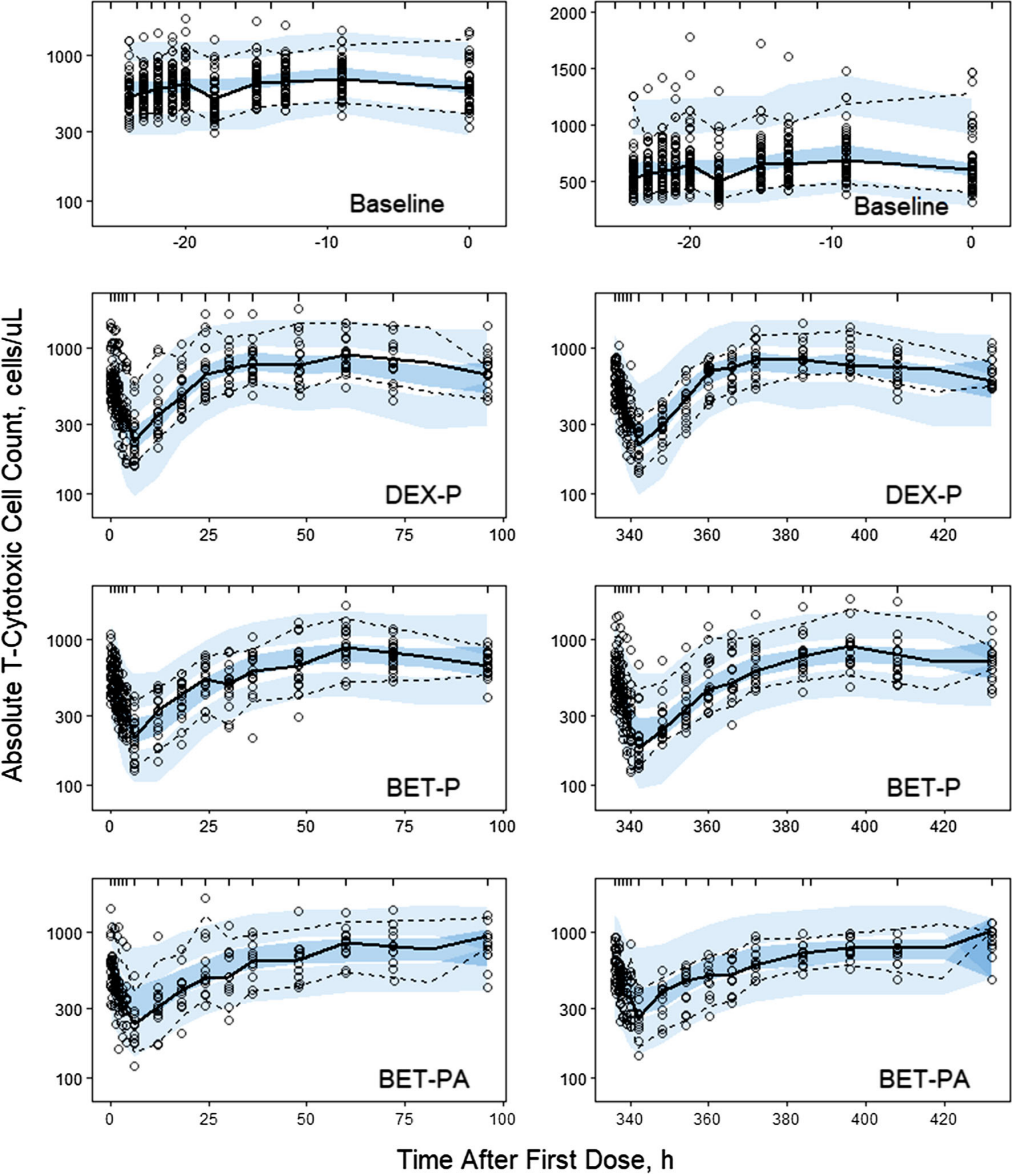
Visual predictive check plots for T-cytotoxic cells following administration of a single IM and PO dose of DEX-P, BET-P, and BET-PA. Graphical features are the same as in Fig. 2

The baseline *TC* profiles shown in Fig. 14 exhibit an asymmetric circadian rhythm with median nadir time -18 h (1 PM), *TC*_*min*_ = 500 ± 175 cells/μL, two peak times around -20 h (11 AM) and -9 h (10 PM), and *TC*_*max*_ = 835 ± 274 cells/μL. Similar to *TH*, the dosed GC suppress *TC* with a nadir range of 219–270 cells/μL followed by a return to the baseline. Because of the same mechanism of action, we applied the model described for *TH* using Eqs. (19)-(21) with *TC*-specific parameters:

22 $$ \frac{dTC}{{dt}} = k_{inTC} \left( {1 - \frac{{C\left( t \right)\left( {IC_{50CTC} /IC_{50TC} } \right) + Cort\left( t \right)}}{{IC_{50CTC} + C\left( t \right)\left( {IC_{50CTC} /IC_{50TC} } \right) + Cort\left( t \right)}}} \right) - k_{beTC} TC $$

with the baseline:

23 $$ \frac{{dTC_{baseline} }}{dt} = k_{inTC} \left( {1 - \frac{{Cort_{baseline} \left( t \right)}}{{IC_{50C} + Cort_{baseline} \left( t \right)}}} \right) - k_{beTC} TC_{baseline} $$

and the unique initial condition that guarantees T-periodicity of *TC*_*baseline*_:

24 $$ TC_{baseline} \left( { - 24} \right) = TC_{baseline} \left( { - T} \right) = TC_{baseline} \left( 0 \right) $$

**Fig. 15:**
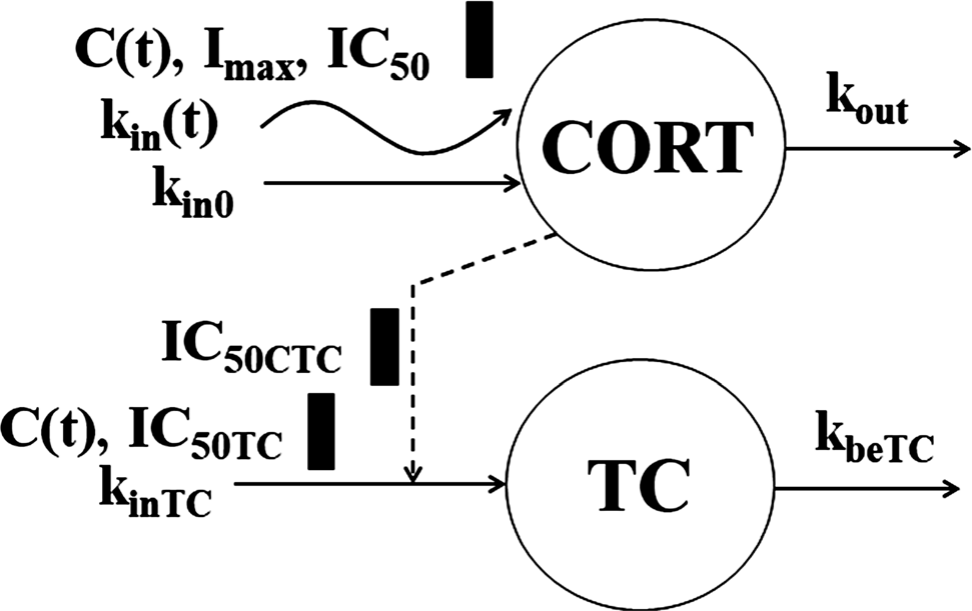
T-cytotoxic cell model where cells (*TC*) are released from the extravascular tissues to the blood at the zero-order rate (*k*_*inTC*_) that is subjected to the circadian rhythm due to the cortisol (*CORT(t)*) inhibition (*IC*_*50CTC*_). The *TC* production is inhibited by corticosteroids (*C(t), IC*_*50TC*_) and removed from blood at the first-order rate *k*_*beTC*_. Symbols are further defined in Table 5

The model diagram is shown in Fig. 15. As for *TH*, all parameters were assumed to be log-normally distributed among subjects according to Eq. (6). The observed *TC*_*ij*_ were log-transformed and the constant residual error model was applied Eq. (7).

The Pre-dose, DEX-P, BET-P, and BET-PA altered *TC* profiles were fitted simultaneously. Parameter estimates are listed in Table 5. We did not detect a significant BOV for *TC* baseline parameters. The relative standard errors of fixed effect parameters did not exceed 12%. The RSE for random effect parameters ranged 6–53%. The 95% confidence region for median predictions captured almost all median observations as shown in Fig. 14. This also applies to the 95th percentile of observations. However, the 5^th^ percentile was underpredicted by the model for DEX-P at 60 h after dosing for both periods. The observed vs. predicted diagnostic plots did not show signs of systematic bias Fig. S17. The overall good agreement between model predictions and observations was confirmed by individual *TC* vs. time plots shown in supplementary Figs. S18–S20.

**Table 5:** Estimates and relative standard errors (%RSE) of parameters for the population PD model of T-cytotoxic lymphocytes. Equations (22)-(24)

Parameter, units	Definition	Typical value (%RSE)	Variance of IIV (%RSE)
*k*_*inTC*_, cells/uL/h	Rate constant of $TC$ transfer from extravascular to blood pool	326 (4.0)	0.0410 (29.0) (20.2)*
*k*_*beTC*_, 1/h	Transfer rate constant from blood to extravascular pool	0.356 (3.7)	0.0254 (43.7) (15.9)*
*IC*_*50TCDEX*_, ng/mL	*IC*_*50*_ for DEX inhibitory effect	15.2 (11.4)	0.184 (45.8) (42.9)*
*IC*_*50TCBET*_, ng/mL	*IC*_*50*_ for BET inhibitory effect	15.1 (7.4)	0.133 (31.4) (36.5)*
*IC*_*50CTC*_, ng/mL	*IC*_*50*_ for cortisol inhibitory effect	131 (12.0)	0.203 (52.7) (45.1)*
σ^2^	Variance of residual error	NA	0.0325 (6.2)

*Variance of log-normally distributed parameter expressed as %CV

**Fig. 16:**
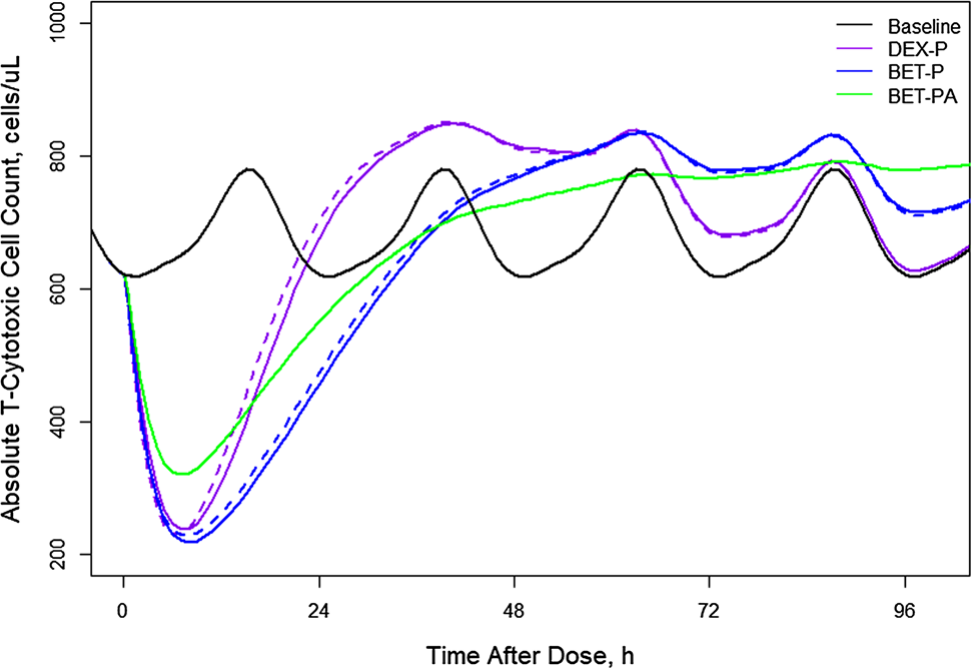
Simulated expected T-cytotoxic cell absolute counts following administration of 6 mg of indicated corticosteroids. Continuous lines indicate IM and dashed lines PO routes

Figure 16 shows simulations of expected *TC* responses following the 6 mg IM and PO doses of DEX-P, BET-P, and BET-PA. The mean of the *TC* circadian baseline is 685 cells/μL with the peak of 778 cells/μL and nadir of 618 cells/μL occurring at −9 h (6 PM) and −23 h (8 AM). The peak and nadir times coincide with those for the mean baseline *TH* profiles. The *TC* responses reach nadirs at 7–8 h of 237 for DEX-P, 218 for BET-P, and 320 cells/μL for BET-PA IM. Subsequently, *TC* returns to the baseline at approximately 23 h (DEX-P), 42 h (BET-P), and 43 h (BET-PA). The response continues rising to create a rebound that ends at about 96 h for DEX-P, and continues beyond 96 h for BET-P and BET-PA. During the suppression phase, *TC* responses do not show circadian oscillations. The differences in *TC* responses between IM and PO dosing is less than 9% for DEX-P and 5% for BET-P.

### Glucose pharmacodynamics

Glucose homeostasis is maintained by food intake and hormonal regulation of hepatic glucose output and glucose uptake primarily by brain, muscle and adipose tissue. The major sources of hepatic glucose output are de novo glucose production (gluconeogenesis) and glycogen breakdown (glycogenolysis). Insulin and glucagon are essential glucose-dependent counter-regulatory hormones that control rates of utilization and production of glucose by tissues. When nutrients are available as occurs after a meal, insulin is secreted from pancreatic beta cells and promotes hepatic glycogen synthesis, lipogenesis, and tissue glucose uptake. When nutrients are scarce, glucagon is secreted from pancreatic alpha cells to promote hepatic glucose production. Normal morning blood glucose concentrations in healthy individuals are about 90 mg/dL. The GC have major influences on gluconeogenesis by affecting the availability of gluconeogenic precursors and the activity of several key gluconeogenic enzymes. Many adverse effects are associated with the chronic use of GC including muscle wasting, hyperglycemia, insulin resistance and diabetes mellitus.

**Fig. 17:**
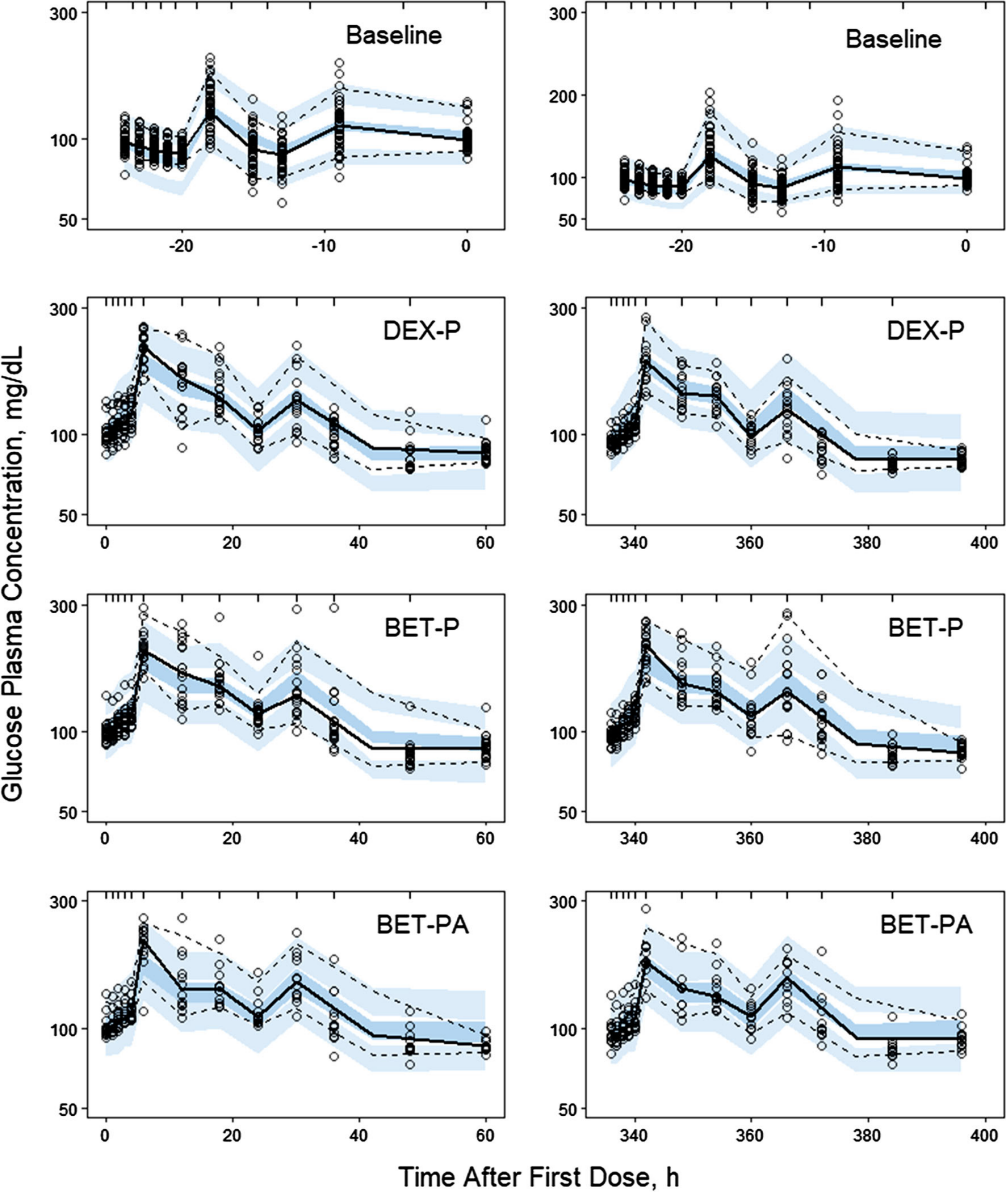
Visual predictive check plots for glucose plasma concentrations following administration of a single IM and PO dose of DEX-P, BET-P, and BET-PA. Graphical features are the same as in Fig. 2

Figure 17 shows baseline and glucose responses after the single doses of GC in the women. The average baseline glucose plasma concentration (*GLUC*) was 98.9 ± 19.1 mg/dL with two distinct peaks at -18 h (1 PM) and -9 h (10 PM) of 130.8 ± 25.5 mg/dL and 114.1 ± 24.1 mg/dL. Given that there were no blood samples taken in the preceding two hours, the peak times correspond to lunch and dinner times. The food effects masked potential circadian variations of the *GLUC* baseline. Administration of GC increased *GLUC* and changed the baseline time course to a profile with two peaks followed by a decline to the baseline values over the 60 h after dosing. The observed peak times were 6 h (1 PM) and 30 h (1 PM) for Period I and 342 h (1 PM) and 366 h (1 PM) for Period II. The peak times were same for all GC responses. The *GLUC*_*max*_ values for Periods I and II were: DEX-P 214.7 ± 27.7 and 192.3 ± 38.0 mg/dL, BET-P 207.7 ± 33.7 and 207.6 ± 33.1 mg/dL, and BET-PA 207.8 ± 37.9 and 185.3 ± 37.5 mg/dL. We did not observe differences in *GLUC* responses between IM and PO doses of DEX-P or BET-P.

**Fig. 18:**
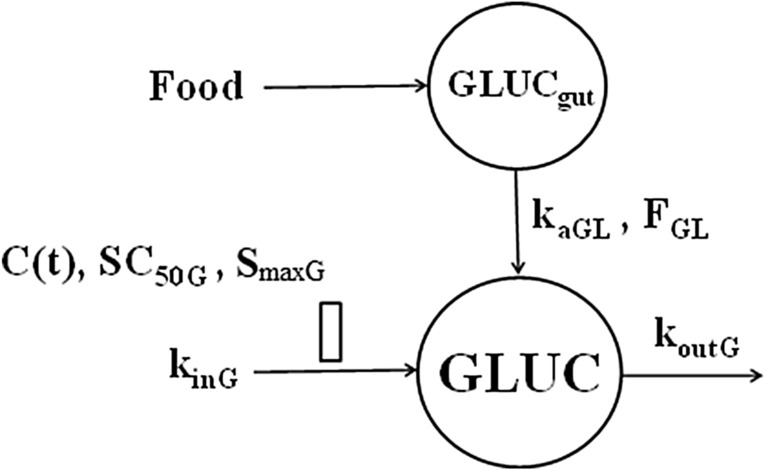
Glucose model where glucose in plasma (*GLUC*) is produced at the zero-order rate (*k*_*inG*_) and removed at the first-order rate (*k*_*outG*_). Corticosteroids stimulate *k*_*inG*_ according to the E_max_ (*S*_*maxG*_, *SC*_*50G*_). Food intake is modeled as a bolus dose of 75 g of glucose into the gut that is absorbed at the first-order rate *k*_*aGL*_ with bioavailability *F*_*GL*_

Given the known stimulatory effect of GC on hepatic glucose output, we selected an indirect response model with the zero-order *GLUC* production rate by the liver *k*_*inG*_ and the first-order rate constant *k*_*outG*_ for glucose utilization by tissues. The GC stimulate *k*_*inG*_ according to the capacity-limited model:

25 $$ \frac{dGLUC}{{dt}} = \mathop \sum \limits_{{i \in \left\{ {B,L,S,D} \right\}}} k_{aGLi} GLUC_{guti} + k_{inG} \left( {1 + \frac{{S_{\max G} C\left( t \right)}}{{SC_{50G} + C\left( t \right)}}} \right) - k_{outG} GLUC $$

where *C(t)* denotes GC plasma concentration, *S*_*maxG*_ is the maximum GC stimulatory effect on the glucose production, and *SC*_*50G*_ is the *C(t)* eliciting 50% of the maximum effect. The model diagram is shown in Fig. 18. The model for food effects on *GLUC* was adapted from [28]. Accordingly, the glucose influx into the plasma due to food intake after breakfast (*B*), lunch (*L*), snack (*S*), and dinner (*D*) was modeled as a bolus dose of *Food* = 75 g of glucose into the gut that was absorbed with first-order rate constant *k*_*aGLi*_ and bioavailability *F*_*GLi*_, *i* $\in${*B,L,S,D*}:

26 $$ \frac{{dA_{guti} }}{dt} = \mathop \sum \limits_{{T_{meal} \in Meali}} F_{GLi} \cdot Food \cdot \delta \left( {t - T_{meal} } \right) - k_{aGLi} A_{guti} $$

where *A*_*guti*_ is the amount of glucose in the gut after meal *i* and *F*_*GLi*_ · *Food · δ(t-T*_*ij*_*)* represents a bolus dose of *F*_*GLi*_* · Food* into the gut at meal times *T*_*meal*_ from a set of all times that the *Meal*_*i*_ was served. The plasma glucose concentrations from the meal *i* were calculated as the ratio *A*_*guti*_/*V*_*GLi*_ where *V*_*GLi*_ denotes the glucose volume after meal *i*. The baseline glucose *GLUC*_*baseline*_ was described by Eq. (25) without the GC effect but with the food effect:

27 $$ \frac{{dGLUC_{baseline} }}{dt} = \mathop \sum \limits_{{i \in \left\{ {B,L,S,D} \right\}}} k_{aGLi} GLUC_{guti} + k_{inG} - k_{outG} GLUC_{baseline} $$

The initial condition for Eq. (27) was

28 $$ GLUC_{baseline} \left( { - 24} \right) = GLUC_{bas} + GLUC_{0} $$

where *GLUC*_*baseline*_ is the *GLUC* value without GC and food effect and *GLUC*_*0*_ is the residual glucose at the beginning of the period. The glucose production rate constant was calculated as

29 $$ k_{inG} = k_{outG} GLUC_{baseline} $$

All parameters were assumed to be log-normally distributed among subjects according to Eq. (6). The observed *GLUC*_*ij*_ were log-transformed and the constant residual error model was applied Eq. (7).

The Pre-dose, DEX-P, BET-P, and BET-PA altered *GLUC* profiles were fitted simultaneously. Parameter estimates are listed in Table 6. We were not able to estimate with reasonable precision the parameters for food effect after breakfast and snack so these were not included. Also, the bioavailabilities *F*_*GLL*_ and *F*_*GLD*_ were not identifiable and were estimated as parts of the apparent volumes *V*_*GLL*_/*F*_*GLL*_ and *V*_*GLD*_/*F*_*GLD*_. We did not detect a significant BOV for *GLUC* baseline parameters. There was no significant improvement in model performance (measured by a drop in the objective function value) when two separate parameters *S*_*maxDEX*_ and *S*_*maxBET*_ were considered, so one parameter *S*_*maxG*_ was assigned to both drugs. We were unable to estimate with satisfactory precision the IIV for *GLUC*_*0*_, *SC*_*50GDEX*_, *SC*_*50GBET*_, *k*_*aGLL*_, and *k*_*aGLD*_, and the variances for these parameters were fixed at 0. The RSE of fixed effect parameters did not exceed 15%, except for *k*_*aGLD*_ (%RSE 41.5%). The RSE for random effect parameters ranged 30–50%. The 95% confidence region for median predictions captured almost all median observations as shown in Fig. 17 except for *GLUC* peaks at 6 h for DEX-P and BET-PA and at 342 h for BET-P. The 5th percentile of observed *GLUC* was underpredicted for the baseline between -24 h and -18 h and for DEX-P responses during 42–60 h. The 95th percentile of observed *GLUC* was slightly underpredicted during the first peak at 6 h for DEX-P, BET-P, and BET-PA, as well as at the last observation times for both periods. The observed vs. predicted diagnostic plots did not show signs of systematic bias for most data except for the highest and lowest values corresponding to the peaks of *GLUC* and ends of the periods (Fig. S21). The overall good agreement between model predictions and observations was confirmed by individual *GLUC* vs. time plots shown in supplementary Figs. S22–S24.

**Table 6:** Estimates and relative standard errors (%RSE) of parameters for the population PD model of glucose Eqs. (25)-(29)

Parameter, units	Definition	Typical value (%RSE)	Variance of IIV (%RSE)
*GLUC*_*bas*_, mg/dL	Glucose baseline in absence of food intake	67.6 (1.7)	0.0078 (32.7) (8.8)**
*GLUC*_*0*_, mg/dL	Glucose at the beginning of period	27.8 (4.9)	0*
*k*_*outG*_, 1/h	First-order rate constant of glucose disposition	0.198 (3.4)	0.0119 (42.5) (10.9)**
*SC*_*50GDEX*_, ng/mL	*SC*_*50*_ for DEX stimulatory effect	17.9 (14.7)	0*
*SC*_*50GBET*_, ng/mL	*SC*_*50*_ for BET stimulatory effect	22.8 (11.1)	0*
*S*_*maxG*_	*S*_*max*_ for DEX and BET effect	1.58 (6.3)	0.0348 (33.3) (18.7)**
*k*_*aGLL*_, 1/h	Glucose absorption rate constant from the gut after lunch	5.44 (5.8)	0*
*k*_*aGLD*_, 1/h	Glucose absorption rate constant from the gut after dinner	2.41 (41.5)	0*
*V*_*GLL*_*/F*_*GLL*_, dL	Apparent volume after lunch	1020 (6.6)	0.104 (30.8) (32.2)**
*V*_*GLD*_*/F*_*GLD*_, dL	Apparent volume after dinner	1340 (9.5)	0.106 (49.7) (32.6)**
*σ*^*2*^	Variance of residual error	NA	0.013 (8.8)

*Parameter was fixed

**Variance of log-normally distributed parameter expressed as %CV

**Fig. 19:**
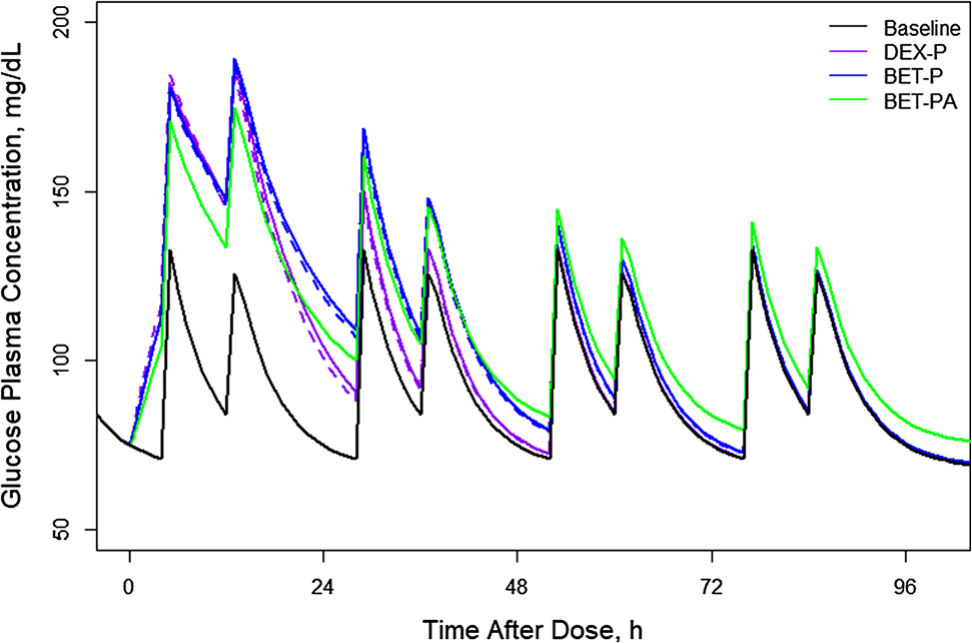
Simulated expected glucose plasma concentrations following administration of 6 mg of indicated corticosteroids. Continuous lines indicate IM and dashed lines PO routes

Figure 19 shows simulations of expected *GLUC* responses following IM and PO doses of 6 mg for DEX-P, BET-P, and BET-PA. The *GLUC* baseline shows only two peaks following lunch and dinner. The mean baseline calculated as AUC/24 is 92.0 mg/d. The lunch peak is 130.0 mg/dL and the dinner peak is 125.0 mg/dL. The *GLUC* IM and PO GC responses are nearly identical. The dosed GC increase *GLUC* with *GLUC*_*max*_ equal to 187.2 for DEX = P, 189.2 for BET-P, and 174.4 mg/dL for BET-PA occurring at 13 h. The *GLUC* peaks in the following days decrease to match the baseline by 3 days after dosing (except for the peak for BET-PA response). The duration of *GLUC* response is shortest for DEX-P that reaches the baseline at about 57 h after dosing and for BET-P at 88 h. The *GLUT* response to the slowly available BET-PA remains slightly elevated beyond 96 h.

### Comparison of sensitivity parameters

**Fig. 20:**
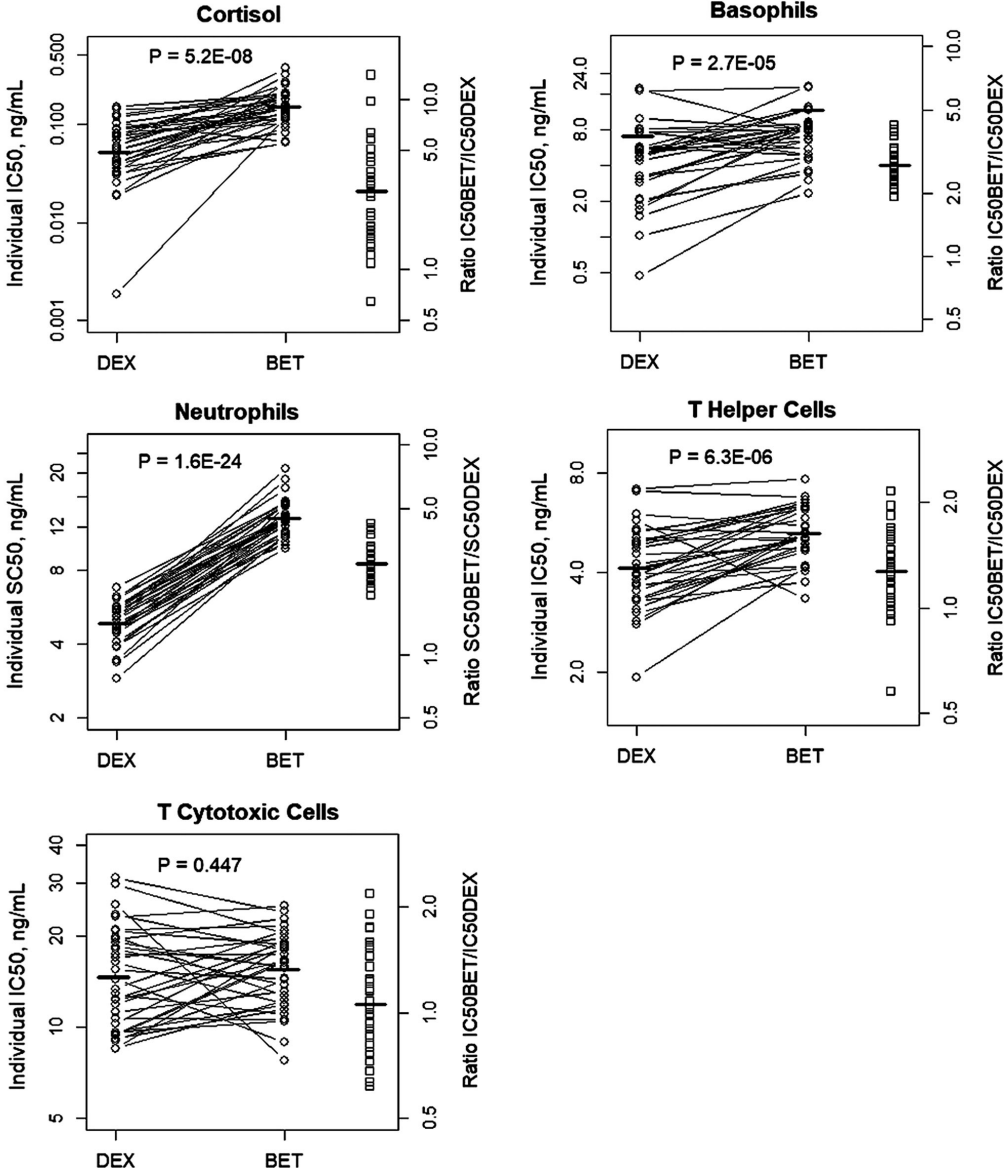
Comparison of individual estimates of sensitivity parameters *IC*_*50*_*/SC*_*50*_ for indicated responses for 33 subjects who received both DEX and BET. The bold lines indicate the geometric means. Specific mean and SD values are listed in Table 7

Subjects assigned to sequences AB, BA, ED, DE, DC, CD received both DEX and BET (N = 33). This allowed comparison of individual estimates of the sensitivity parameters using comparison plots and paired t-tests for their log-transformed values. Figure 20 shows the graphical comparison and the geometrical means of *SC*_*50*_*/IC*_*50*_ values for individual available subjects and the P-values are listed in Table 7. As the IIV for glucose *SC*_*50*_ were not determined, the individual glucose *SC*_50_ values were not available for the graphical comparison in Fig. 20. Also, the typical values were reported in Table 7. The geometrical means agreed with the estimates of the typical values. The P values indicated highly significant (P < 0.0001) differences between DEX and BET for all responses except for *TC* (P = 0.447). The *SC*_*50*_*/IC*_*50*_ values for DEX were consistently smaller than for BET. The mean ratios of *IC*_*50BET*_*/IC*_*50DEX*_ or *SC*_*50BET*_*/SC*_*50DEX*_ were: 2.86 (*CORT*), 1.72 (*BASO*), 2.69 (*ANC*), 1.27 (*TH*), 1.06 (*TC*), and 1.27 (*GLUC*).

**Table 7:** Comparison of DEX and BET sensitivity parameters for PD biomarkers. The geometric means were calculated for 33 subjects in the study who received both DEX and BET. The p-values were calculated using the paired two-tail t-test of the log-transformed individual estimates

PD Biomarker	Parameter	Geometric Mean (SD) ng/mL BET	Geometric mean (SD) ng/mL DEX	Geometric mean of Individual Ratios BET/DEX	P-value
Cortisol	*IC*_*50*_	0.149 (1.5)	0.0522 (2.25)	2.86	5.2E-08
Basophils	*IC*_*50*_	7.20 (1.6)	4.19 (2.1)	1.72	2.7E-05
Neutrophils	*SC*_*50*_	13.02 (1.3)	4.84 (1.4)	2.69	1.6E-024
T-Helper Cells	*IC*_*50*_	5.26 (1.2)	4.13 (1.3)	1.27	6.3E-06
T-Cytotoxic Cells	*IC*_*50*_	15.5 (1.3)	14.7 (1.5)	1.06	0.45
Glucose	*SC*_*50*_	22.8*	17.9*	1.27*	NA

*Typical value of the parameter

Simulations of DEX and BET plasma concentrations for the three dosing regimens for antenatal corticosteroid treatment were performed: DEX-P as four doses of 6 mg given at 12-h intervals (6 mg IM BIDx4), BET-P as two doses of 12 mg given at a 24-h interval (12 mg IM QDx2), and BET-PA as two doses of 12 mg given at a 24-h interval (12 mg IM QDx2). Figure S25 shows the simulated PK profiles for these three regimens overlaid with the typical values of sensitivity parameters for all studied responses for the relevant GS. The median peaks and troughs were reported in [5]. The *C*_*troughDEX*_ = 33.1 ng/mL for DEX-P 6 mg IM BIDx4 as well as *C*_*troughBET*_ = 26.4 ng/mL for BET-P 12 mg IM QDx2 were higher than all *IC*_*50*_*/SC*_*50*_ values. Only *C*_*troughBET*_ = 17.1 ng/mL for BET-PA 12 mg IM QDx2 was lower than its sensitivity for glucose *SC*_*50GBET*_ = 22.8 ng/mL, but still higher than BET sensitivities for other responses. Both DEX and BET plasma concentrations for DEX-P and BET-P dosing regimens fell below sensitivity levels for all responses except for *CORT* by 72 h after the first dose. The plasma concentrations for BET-PA fell below the sensitivities for* GLUC* and *TC* responses by that time. Only DEX stopped suppressing cortisol after 96 h. The BET plasma concentrations remained elevated above *IC*_*50CBET*_ for the duration of the simulated regimen over 168 h.

## Discussion

An indirect response model with a circadian input *k*_*in*_*(t)* is the most commonly model used to describe the GC effects on cortisol production. What varies are the mathematical functions representing *k*_*in*_*(t)* given the asymmetric irregular baseline. We selected a two-harmonic function as a sum of two cosines of periods 24 and 12 h as it has been shown to adequately describe the cortisol baseline in healthy volunteers [20]. Since circadian oscillations were almost totally suppressed during the nadir period and its value was same for all GC and greater than the LOQ of 1 ng/ml, we explained this by the presence of a constant small cortisol baseline and production that is not subject to circadian regulation. The low constant cortisol baseline may have not been detected in some previous studies owing to values falling below the LOQ of applied cortisol assays. However, this phenomenon has been noted in at least one previous study [29].

Our estimates of the mean cortisol plasma concentration *a*_*0*_ = 42.0 ng/mL and the elimination rate constant *k*_*out*_ = 0.378 h^−1^ are consistent with previous reports [6, 20]. The estimated mean baseline value 3.93 ng/mL is slightly above the range of observed nadirs. We set the maximum inhibition parameter *I*_*max*_ at 1.0 to enforce the reported GC ability of 100% suppression of cortisol secretion by the adrenal cortex. Our estimate of the typical DEX sensitivity *IC*_*50DEX*_ of about 0.05 ng/mL is lower than reported in white male subjects of 0.1 ng/mL [6, 30], although the models differ slightly. We are not aware of any application of an indirect response model to estimate *IC*_*50BET*_ in humans. Our simulations show a longer duration of suppression of cortisol by BET than by DEX despite *IC*_*50BET*_ > *IC*_*50DEX*_. This is a consequence of the longer BET half-life (17 h) than DEX (7.5 h) resulting in longer times above *IC*_*50*_ for BET, and subsequently overall stronger inhibition.

The effect of GC on basophil counts in healthy volunteers was described previously by a two-compartment indirect response model representing blood and extravascular basophil pools [31, 32]. While the inhibition of basophil trafficking from the extravascular tissues is capable of generating a rebound, such late data were absent in these reports due to the 32 h duration of the study. When we applied this simpler model to our data, it performed significantly poorer in terms of goodness-of-fit and magnitude of standard errors.

Our estimate of the mean baseline basophil count is in the normal range of 0–300 cells/μL and includes the mean of observed values of 26.8–35.8 cells/μL reported for this subject population [4]. The estimate of *k*_*outBASO*_ = 0.137 h^−1^ is higher than one value of 0.277 h^−1^ reported [32]. This discrepancy can be explained by the difference between the model structures and data sets used for parameter estimation. The value of 1/*k*_*outBASO*_ = 7.3 h can be interpreted as the mean residence time of basophils in blood and it can be compared to the reported basophil lifespan of 1–2 days [23]. This short residence time would imply that the majority of basophils do not circulate but rather reside in the margination pool or extravascular tissues. By the same token 1/*k*_*ptBASO*_ = 21.6 h can be interpreted as the mean residence time for the basophil precursors in the bone marrow. The estimated sensitivity of DEX was slightly lower than for BET but both GC administered as phosphate formulations yielded nearly the same basophil nadirs. The DEX response returned to the baseline faster than BET response owing to the shorter half-life. To our knowledge the sensitivity parameters for BET and DEX inhibitory effect on human basophils *IC*_*50BASOBET*_ and *IC*_*50BASODEX*_ have not been reported before.

The normal *ANC* range for women is 2000–7000 cells/μL that contains our estimated mean value *a*_*0ANC*_ = 4900 cells/μL. Our model predicts the peak time of typical *ANC* profile at 1 PM, agreeing with the observed data, and the nadir time at 8 PM that differs from the median observed of 9 AM. However, due to the two-harmonic nature of the modeled *ANC* baseline, our model predicts a secondary nadir at 7 AM of 4775 cells/μL that differs from *ANC*_*min*_ by 389 cells/μL. We detected a significant BOV for only two baseline inc parameters *a*_*0ANC*_ and *b*_*1ANC*_, which resulted in a decrease of the maximum likelihood objective function value by 202.2. Our estimate of the typical value *k*_*outANC*_ implies the neutrophil residence time in the circulation of 1/*k*_*outANC*_ = 10.8 h that agrees with the reported neutrophil lifespan. The indirect response model with constant input was applied by Mager et al. [6] to describe DEX effect on *ANC*. They reported *k*_*outANC*_ = 0.071 h^−1^ that is close to our estimate. The estimated sensitivity of DEX was 2.7-fold lower than one of BET, but the predicted ANC peak was slightly higher for BET-P than DEX-P with a much faster return to the baseline of the *ANC* stimulated by the latter. This can be explained by a shorter DEX (7.5 h) than BET half-life (17 h). The *SC*_*50ANC*_ = 6.07 ng/mL reported in white males [6] for DEX is close to our estimate of *SC*_*50ANCDEX*_ = 4.92 ng/mL. The estimates of typical values *S*_*maxANC*_ for DEX and BET were similar.

The PD model for lymphocyte trafficking does not contain an extravascular lymphocyte pool. As the size of this pool is many-fold larger than the blood pool, its dynamics are not significantly perturbed by *k*_*in*_ and *k*_*be*_ processes, which justifies using *k*_*in*_ as a zero-order rate constant rather than a first-order one. The model predicted nadir and peak times for typical baseline T-helper cells agree with the observed times. The *TC* nadir time predicted by our model occurs a few hours earlier whereas the *TC* agrees with the observed one. The peak and nadir for typical *TH* and *TC* responses are close to the observed mean values*.* Our estimates of the typical value of *k*_*beTH*_ and *k*_*beTC*_ are very similar to the values reported by Mager et al. [6] as are the *IC*_50_ values. These rate constants allow us to calculate the residence times in the blood for T-helper lymphocytes 1/*k*_*beTH*_ = 2.2 h and T-cytotoxic lymphocytes 1/*k*_*beTC*_ = 2.8 h. Interestingly, the cortisol sensitivity (*IC*_*50*_) for the suppression of *TH* is more than tenfold lower than analogous sensitivities for DEX and BET. The same applies to the cortisol sensitivity for suppression of *TC*. This difference is consistent with sensitivities of other GC reported elsewhere [6, 7, 13]. Administration of DEX-P and BET-P resulted in the same degree of *TH* and *TC* suppression, however the suppressive effect of DEX was of lesser duration owing to its shorter half-life. The rebound observed for all studied GC has been reported previously [6, 7]. This can be attributed to the prolonged suppression of cortisol by these drugs that results in a lesser cortisol suppressive effect on lymphocyte trafficking than during baseline conditions when cortisol is higher.

The PK/PD models of the glucose-insulin system are well established for variety of perturbations such intravenous glucose tolerance test, glucose clamp, insulin dosing, anti-diabetic drugs, meals and other factors in animals, healthy subjects and diabetic patients (see for example [28, 33, 34]). Few models of GC effects on glucose have been developed. Derendorf et al. [35] applied an *Emax* model to describe the effect of IV bolus administration of DEX-P on glucose plasma concentrations in healthy subjects assuming that peripheral compartment concentrations were the biophase. In the absence of insulin measurements, we selected a parsimonious turnover model for glucose production and utilization where all factors of the insulin-glucose system regulation were represented by the glucose production and disposition rate constants. The exception was the food effects on the glucose baseline that was apparent in our data. The food effect model was adapted from [28] where glucose input from the gut after meals was described as a first-order absorption process adjusted by a bioavailability specific for each meal. We were only able to identify the effects of lunch and dinner. The estimates of corresponding absorption rates 5.44 h^−1^ and 2.41 h^−1^ are tenfold higher than analogous values reported in [28]. There the glucose input to the gut was modeled as an infusion over the duration of the meal that might contribute to these differences. Our estimate of a typical glucose concentration *GLUC*_*bas*_ + *GLUC*_*0*_ = 95.4 mg/dL agrees with the normal glucose value in healthy individuals. The estimate of 0.198 h^−1^ for the glucose disposition rate constant from plasma is close to the value 0.33 h^−1^ from [28]. Similar to other GC responses, giving DEX-P and BET-P resulted in the same degree of *GLUC* stimulation that was weaker for BET-P. The duration of effects of DEX was shorter owing to its shorter half-life. Our estimates of *S*_*maxG*_ = 1.58 implies a moderate 1.5-fold maximal stimulation of the glucose production by GC. The sensitivities *SC*_*50GDEX*_ and *SC*_*50GBET*_ are similar with glucose being slightly more responsive to DEX than BET. The values of *E*_*max*_ = 82.3 mg/dL and *EC*_*50*_ = 8.6 ng/mL estimated in [35] for DEX using the *E*_*max*_ model differ from our *GLUC*_*bas*_*⋅ S*_*maxG*_ = 106.8 mg/dL and *SC*_*50GDEX*_ = 22.8 ng/mL owing to different model structures. However, another study reported an *EC*_50_ of 26.9 ng/mL for glucose induction by DEX [30].

The PD parameters that relate to efficacy and potency of GC are *I*_*max*_*/S*_*max*_ and *IC*_*50*_*/SC*_*50*_. While the inhibitory effects *I*_*max*_ was set to its maximal value of 1.0 either based on observed data or estimates that reached a boundary, the maximal stimulatory estimates of GC on *ANC* and *GLUC* were in the range 1 to 3, which implies a moderate 100–300% capacity to increase the input rate of neutrophils and glucose to the blood. The *S*_*max*_ did not differ between DEX and BET for glucose. However, it was significantly higher for BET than DEX stimulation of *ANC*, although with less than 20% difference. The sensitivity parameters *IC*_*50*_*/SC*_*50*_ were estimated for all responses. Their individual estimates for subjects who received both DEX and BET permitted within-subject comparisons. Consistently for all responses, DEX sensitivities were lower than ones for BET, implying stronger DEX activity. This activity was offset by a shorter half-life for DEX, so we did not observe marked differences between maximal responses to DEX-P and BET-P. The exception was BET-PA that produced weaker but prolonged responses due to its long half-life attributed to slow hydrolysis and absorption [5]. Our simulations for three clinically applied dosing regimens revealed that the troughs of GC plasma concentrations were much higher than the sensitivities of all responses, which resulted in duration of effects more than 3 days after the first dose. In the case of cortisol, these effects lasted more than 4 days for the DEX regimen and more than a week for the BET regimens.

### Pharmacometric considerations

The present study offers several strengths in context of PK/PD modeling capabilities. The PK and measurement of biomarkers was carried out for 96 h allowing excellent definition of the absorption and disposition phases of the various drug formulations, especially that of the slow release component of BET-PA. In fact, this study provided the first clear assessment of the PK of BET released from BET acetate in man [5]. Measurement of the biomarkers over 96 h provided full onset, maxima, and return phases for all profiles except for the tail from BET-PA. This helped reveal for the first time that basophils exhibit a rebound phase that necessitated use of the precursor model (Fig. 6). The relatively large 6 mg doses of DEX and BET produce early plasma concentrations that were much higher than *IC*_*50*_ or *SC*_*50*_ values producing maximum responses that were often evident in the data. It was demonstrated [36] that single large drug doses allow reliable estimations of the parameters of indirect response models when several dose levels cannot be studied. A previous study [35] where IV DEX-P doses of 20, 50, and 80 mg were given to healthy volunteers showed maximum increases in *GLUC* and decreases in lymphocytes similar to ours at 6 mg indicating reliable attainment of maximal changes. It was unrealistic to carry out a 5-way full cross-over study in 48 women, but the fact that 33 women received a form of both BET and DEX allowed direct comparison of their sensitivities (Fig. 20) and PK including clearances [5], which were the key purposes of this study.

All women in this study were of similar age, body weight, and the same ethnicity. Such a homogenous subject population is a limitation for extrapolating the present results to more diverse populations. However, a study of prednisolone effects on PD markers including cortisol, neutrophils, and lymphocytes indicate no significant difference between white and black healthy women [37]. Another study reported a modest difference between white and black healthy subjects in inhibition of ex-vivo T-lymphocyte proliferation by DEX [38]. Similar findings were reported regarding white/black differences in DEX effect on ex vivo proliferation of peripheral blood mononuclear cells [39]. As indicated earlier in the Discussion, the sensitivity values from our Indian women were generally similar to values reported in white male subjects [6]. Any actual differences are in the range seen with sex differences in PD [37]. Publications comparing differences in PD responses to between other ethnic groups (e.g. Asian) could not be found. Thus there is some uncertainty in applying these PD findings to other ethnic groups.

The PK/PD modeling demonstrated a population approach to the assessment of six biomarkers that are strongly affected by GC in man with four requiring inclusions of complex circadian rhythms. The metrics and diagnostic plots for all biomarkers show reasonable to excellent characterization of the data. While these complex indirect response models were applied previously or are extensions thereof that were employed for effects of GC, they are complicated by the need to ascertain and apply multiple harmonics for the circadian rhythms and the requirement to include the PK/PD of cortisol for the cell dynamic profiles in order to dissect the drug sensitivities from those of cortisol. Identifying the initial conditions of the circadian model equations required some innovations.

Our approach towards modeling the circadian rhythm in the observed data was to model the baseline *R*_*baseline*_*(t)* and subsequently calculate the input rate *k*_*in*_*(t)* from a differential equation describing *R*_*baseline*_*(t)*. Since our models consisted of indirect responses, such calculation was straightforward. This approach can be extended to more complex models (e.g. physiologically based models, quantitative systems pharmacology models) with circadian baselines, assuming *k*_*in*_*(t)* can be explicitly solved for implementing the model differential equations. An advantage of modeling the baseline is that baseline parameters such as mesor and amplitude can be identified from the observed data. Another advantage is having explicit equations for the initial conditions. A challenge for this approach is lack of control of the sign of *k*_*in*_*(t)* that we discuss in the following paragraph. An alternative approach is to model *k*_*in*_*(t)* and use a differential equation to describe *R*_*baseline*_*(t)*. This way of modeling circadian responses is more common as it does not require additional calculations involving *R*_*baseline*_*(t)* and model complexity is not a limitation (see for example [20]). A challenge becomes the initial condition for a differential equation describing *R*_*baseline*_*(t)* that usually cannot be explicitly calculated.

One challenge of modeling circadian baselines with biorhythmic functions (e.g. two harmonics) is that when they become baselines for indirect response models with circadian inputs, the latter are uniquely determined by the model differential equations. Consequently, the response production rate can become negative at some time points. If the baseline is a single harmonic, the production rate is described by a cosine function that is always positive as long as its amplitude is greater than its mesor. If the baseline is described by two harmonics, then the response production is described by two cosine functions. A sufficient condition for its positiveness is that the mesor be greater than the sum of the cosine amplitudes. However, this is not a necessary condition as it is for the single cosine case. We introduced a new parameterization of the mean baseline that guarantees positiveness of the circadian input (Appendix 1). Another challenge for indirect response models with circadian inputs is providing initial conditions for model differential equations. By definition, these are the baseline response values at the initial time *t* = 0. If the baseline equations do not have an explicit form (e.g. our models for T helper and T cytotoxic cell responses), a common technique to set up initial conditions is to start solving model equations at a large negative time with arbitrary initial conditions, such that by the time *t* the solution will reach the baseline as a consequence of its stability. However, this approach does not specify the initial negative time that is always assumed to be overly ample and subsequently increases running times for numerical solvers. We introduced a new approach (Appendix 2) where two additional differential equations are introduced with the initial time *t*_*0*_* -T* (negative period) and zero initial conditions, such that at time *t*_*0*_ they provide a solution that is equal to the baseline response at time *t*_*0*_ that is a starting point for the model differential equations. This extends the computer running times to calculate the solution only over an additional period at the expense of two extra differential equations.

### Therapeutic relevance

The increases in plasma glucose concentrations (Fig. 17) demonstrate why these drugs are called glucocorticoids. Such changes are often of concern in patients receiving GC therapy [40]. The biomarker demonstrating the greatest sensitivity to both DEX and BET is cortisol (Fig. 20). Thus, adrenal suppression appears unavoidable when giving GC, especially chronically and at higher doses and many regimens call for tapering to allow normalization of the HPA axis [41].

These studies were enacted to compare the PK and PD of the dosage forms of BET and DEX that are used or are possible for treatment of pregnant women at risk of premature delivery [3]. The WHO-recommended dosing regimens that were simulated (Fig. S25) have been considered equivalent. The single-dose biomarker PD profiles for *CORT* (Fig. 4), *BASO* (Fig. 7), *ANC* (Fig. 10), *TH* (Fig. 13), *TC* (Fig. 16), and *GLUC* (Fig. 19) show similar early maximum effects but moderate later differences that occur owing to the longer half-lives of BET and BET-PA. Simulations of the full multiple-dose profiles after IM doses of DEX-P, BET-P, and BET-PA show lower *C*_*max*_ and *C*_*min*_ GC concentrations after BET-PA, but extended washout concentrations. All of the biomarkers are expected to be strongly affected during the first 48 h after dosing is initiated, but the slowly-releasing BET-PA persists longest. However, these simulations are based on the PK/PD parameters of our nonpregnant women and changes in PK and possibly PD along with uncertainties of effects on the fetus or newborn infant need consideration.

Important new findings from this study are that the bioavailabilities of IM and PO DEX-P and of IM and PO BET-P are essentially equivalent with the small differences in absorption rates (PO faster than IM) not expected to affect their PD (Figs. 4, 7, 10, 13, 16, 19). This argues for use of PO DEX or PO BET in situations where the IM dosage forms are not available.

Lastly, the use of 6 mg doses of DEX-P have been found effective for treating the cytokine storm that often accompanies COVID-19 infections [42]. Our study shows the very strong effects of 6 mg DEX on immune biomarkers such as T-cells. Such effects may be even stronger when patients have altered PK owing to compromised hepatic, renal, and cardiac function.

## Summary

In summary, we performed population PD analysis of six biomarkers for GC effects in healthy Indian women. This followed our population analysis of the PK data for this study [5]. We adopted mechanistic but parsimonious PD models largely published before to describe these effects. The major underlying complexity were circadian rhythms that needed to be incorporated in PD models for cortisol, neutrophils, T helper cells, and T cytotoxic cells. The estimated inter-individual variability of most PD parameters was modest owing to the homogeneous ages, weights, and ethnicities of the women. Our estimates for the baseline parameters generally agreed with values reported previously. The GC exhibited both stimulatory and inhibitory effects on processes regulating production of the six biomarkers. The inhibitory effects were expressed at 100% capacity for the doses used whereas the stimulatory effects were in the range of 100 to 300%, but probably at maximum as well. The BET sensitivities were generally 1.06 to 2.86 times weaker than DEX sensitivities. The higher DEX activity (lower sensitivity values) was offset by its shorter half-life resulting in modest differences in the overall responses to DEX-P and BET-P formulations. The responses to the BET phosphate/acetate formulation were weaker and prolonged due to the extremely slow bioavailability from BET acetate.

### Supplementary Information

Below is the link to the electronic supplementary material.

Supplementary material 1 (TXT 4 kb)

Supplementary material 2 (PDF 1324 kb)

Supplementary material 3 (TXT 4 kb)

## Constraints on the baseline cortisol parameters to ensure ***k***_***in***_***(t)*** ≥ 0

Since *k*_*in*_*(t)* is defined by the baseline cortisol *Cort*_*baseline*_*(t)*, Eq. (4) one must ensure that *k*_*in*_*(t)* is not negative for all times. A necessary and sufficient condition for that is:

$$ \mathop {\min }\limits_{0 \le t \le T} k_{in} \left( t \right) = k_{in} \left( {t_{\min } } \right) \ge 0 $$

where *t*_*min*_ is a solution to:

$$ k_{out} a_{0} + \left( {k_{out} a_{1} + \frac{2\pi }{T}b_{1} } \right)\cos \left( {\frac{2\pi }{T}t_{\min } } \right) + \left( {k_{out} b_{1} - \frac{2\pi }{T}a_{1} } \right)\sin \left( {\frac{2\pi }{T}t_{\min } } \right) + \left( {k_{out} a_{2} + \frac{4\pi }{T}b_{2} } \right)\cos \left( {\frac{4\pi }{T}t_{\min } } \right) + \left( {k_{out} b_{2} - \frac{4\pi }{T}a_{2} } \right)\sin \left( {\frac{4\pi }{T}t_{\min } } \right) = 0 $$

Using the equivalent cosine representation:

$$ k_{out} a_{0} + R_{1} \cos \left( {\frac{2\pi }{T}(t_{\min } - t_{1} )} \right) + R_{2} \cos \left( {\frac{4\pi }{T}(t_{\min } - t_{2} )} \right) = 0 $$

where

$$ R_{i} = \sqrt {\left( {k_{out} a_{i} + \frac{2\pi }{T}b_{i} } \right)^{2} + \left( {k_{out} b_{i} - \frac{2\pi }{T}a_{i} } \right)^{2} } ,i = 1,2 $$

and *t*_*i*_ is the peak time (acrophase) for *i*^*th*^ harmonic, a sufficient condition for *k*_*in*_*(t*_*min*_*)* ≥ 0 is:

$$ a_{0} \ge \frac{{R_{1} + R_{2} }}{{k_{out} }} $$

It was implemented as:

$$ a_{0} = A_{0} + \frac{{R_{1} + R_{2} }}{{k_{out} }} $$

$$ {\text{and}} \, A_{0} \ge {0}. $$

## Representation of ***TH***_***baseline***_***(t)*** by solutions of two differential equations with zero initial conditions

Let us denote for shortness:

$$I\left(t\right)=1-\frac{{Cort}_{baseline}(t)}{{IC}_{50C}+{Cort}_{baseline}(t)}$$

for any−∞ < t < ∞. Since *Cort*_*baseline*_*(t)* is *T*-periodic (see Eq. (2)), *I(t)* is also *T*-periodic. Let us define for -2*T* < *t* two auxiliary functions *TH*_*0*_*(t)* and *TH*_*1*_*(t)* as solutions to the following:

A1 $$\frac{{dTH}_{0}}{dt}=\theta (-T-t)\left({k}_{inTH}I\left(t\right)-{k}_{be}{TH}_{0}\right)$$

A2 $$\frac{{dTH}_{1}}{dt}=\theta (t+T)\left({k}_{inTH}I\left(t\right)-{k}_{be}\left({TH}_{1}+\frac{{TH}_{0}}{1-\mathrm{exp}(-{k}_{be}T)}\right)\right)$$

with initial conditions:

$${TH}_{0}\left(-2T\right)=0$$ and $${TH}_{1}\left(-2T\right)=0$$ (A3).

Here *θ(x)* = 0 if *x* < 0, and *θ(x)* = 1 if *x* ≥ 0. Then the function:

A4 $${TH}_{baseline}\left(t\right)={TH}_{1}\left(t\right)+\frac{{TH}_{0}(t)}{1-\mathrm{exp}\left(-{k}_{be}T\right)}$$

satisfies Eqs. (20)-(21) for *–T* < *t*. To show that, one can notice that for *–T* < *t*, *θ(t* + *T)* = 1 and *θ(–t–T)* = 0. This implies that *TH*_*0*_*(t)* is constant and

A5 $${TH}_{0}\left(t\right)={TH}_{0}(-T)$$

A6 $$\frac{{dTH}_{1}}{dt}={k}_{inTH}I\left(t\right)-{k}_{be}{TH}_{baseline}$$

Since *TH*_*0*_*(t)* is constant (A4) implies that:

$$\frac{{dTH}_{1}}{dt}=\frac{{dTH}_{baseline}}{dt}$$

which proves that *TH*_*baseline*_*(t)* satisfies Eq. (20). Since *θ(t* + *T)* = 0, for -2*T* < *t* < *-t*, Eq. (A2) and (A3) imply that

A7 $${TH}_{1}\left(-T\right)=0$$

Hence:

A8 $${TH}_{baseline}\left(-T\right)=\frac{{TH}_{0}(-T)}{1-\mathrm{exp}\left(-{k}_{be}T\right)}$$

To show Eq. (21) holds true, it suffices to prove that *TH*_*1*_(0) = 0. Applying the integrating factor technique to solve Eq. (A2) with the initial condition (A7) yields a solution:

A9 $${TH}_{1}\left(t\right)={\int }_{-T}^{t}\left({k}_{inTH}I\left(z\right)-\frac{{k}_{be}{TH}_{0}(-T)}{1-\mathrm{exp}(-{k}_{be}T)}\right)\mathrm{exp}\left({k}_{be}(z-t)\right)dz$$

At *t* = 0 Eq. (A9) simplifies to:

A10 $$ TH_{1} \left( 0 \right) = \mathop \smallint \limits_{{ - T}}^{0} k_{{inTH}} I\left( z \right)\exp \left( {k_{{be}} z} \right)dz - TH_{0} \left( { - T} \right) $$

Applying the integrating factor technique to solve Eq. (A1) with the initial condition (A3) yields a solution:

A11 $${TH}_{0}\left(t\right)={\int }_{-2T}^{t}{k}_{inTH}I\left(z\right)\mathrm{exp}\left({k}_{be}(z-t)\right)dz$$

Hence:

A12 $$  TH_{0} \left( { - T} \right) = \mathop \smallint \limits_{{ - 2T}}^{{ - T}} k_{{inTH}} I\left( z \right)\exp \left( {k_{{be}} \left( {z + T} \right)} \right)dz = \mathop \smallint \limits_{{ - T}}^{0} k_{{inTH}} I\left( {s - T} \right)\exp \left( {k_{{be}} s} \right)ds $$

Since *I(t)* is *T*-periodic, Eqs. A10 and A12 imply that *TH*_*1*_(0) = 0, which completes the proof that *TH*_*baseline*_*(t)* satisfies Eq. (21).
