# Supplementary material for: Population pharmacodynamic modeling of intramuscular and oral dexamethasone and betamethasone effects on six biomarkers with circadian complexities in Indian women
Source: J Pharmacokinet Pharmacodyn. 2021 May 5;48(3):411–38. doi: 10.1007/s10928-021-09755-y (PMC8099395; doi:10.1007/s10928-021-09755-y)
Supplement: Supplementary file 2 — Supplementary material 2 (PDF 1324 kb) [file 10928_2021_9755_MOESM2_ESM.pdf]

Supplemental Materials

Population Pharmacodynamic Modeling of Intramuscular and Oral Dexamethasone and Betamethasone Effects on Six Biomarkers with Circadian Complexities in Indian Women

Wojciech Krzyzanski, Mark A Milad, Alan H Jobe, Thomas Peppard, Robert M Bies, William J Jusko

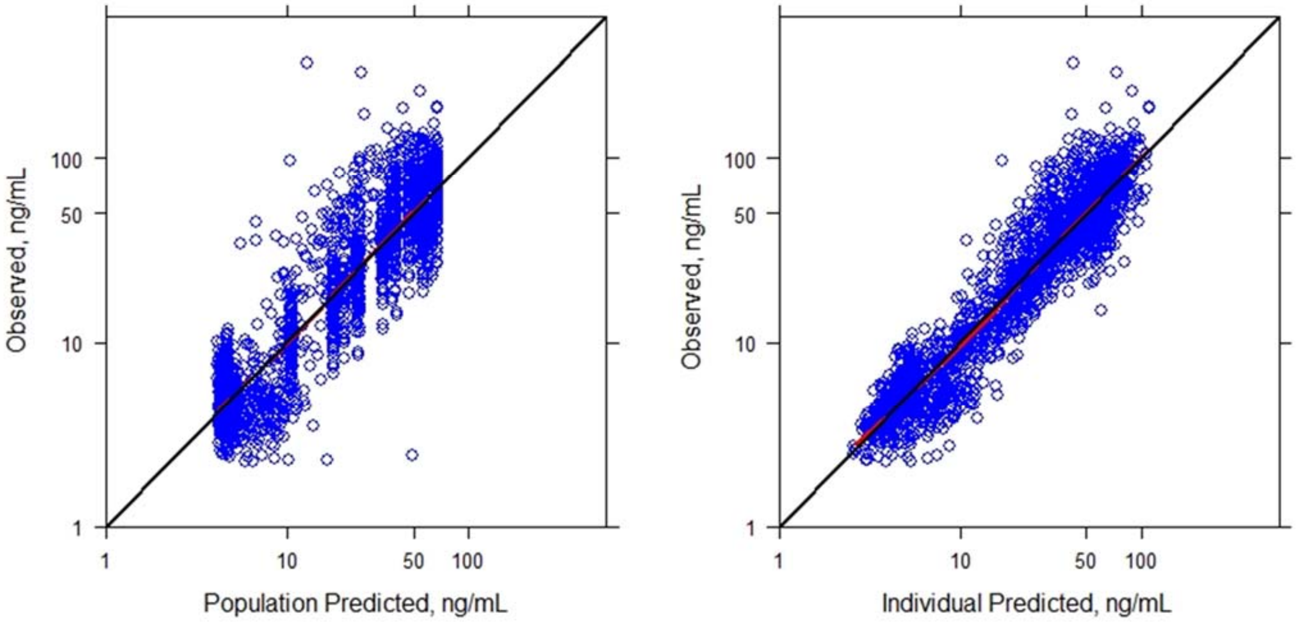

Figure 1S. Observed vs population predicted (left) and individual predicted (right) diagnostic plots for population PD model of cortisol, Eqs. (1)-(7). The black line is the identity, while the red line is the LOESS regression curve.

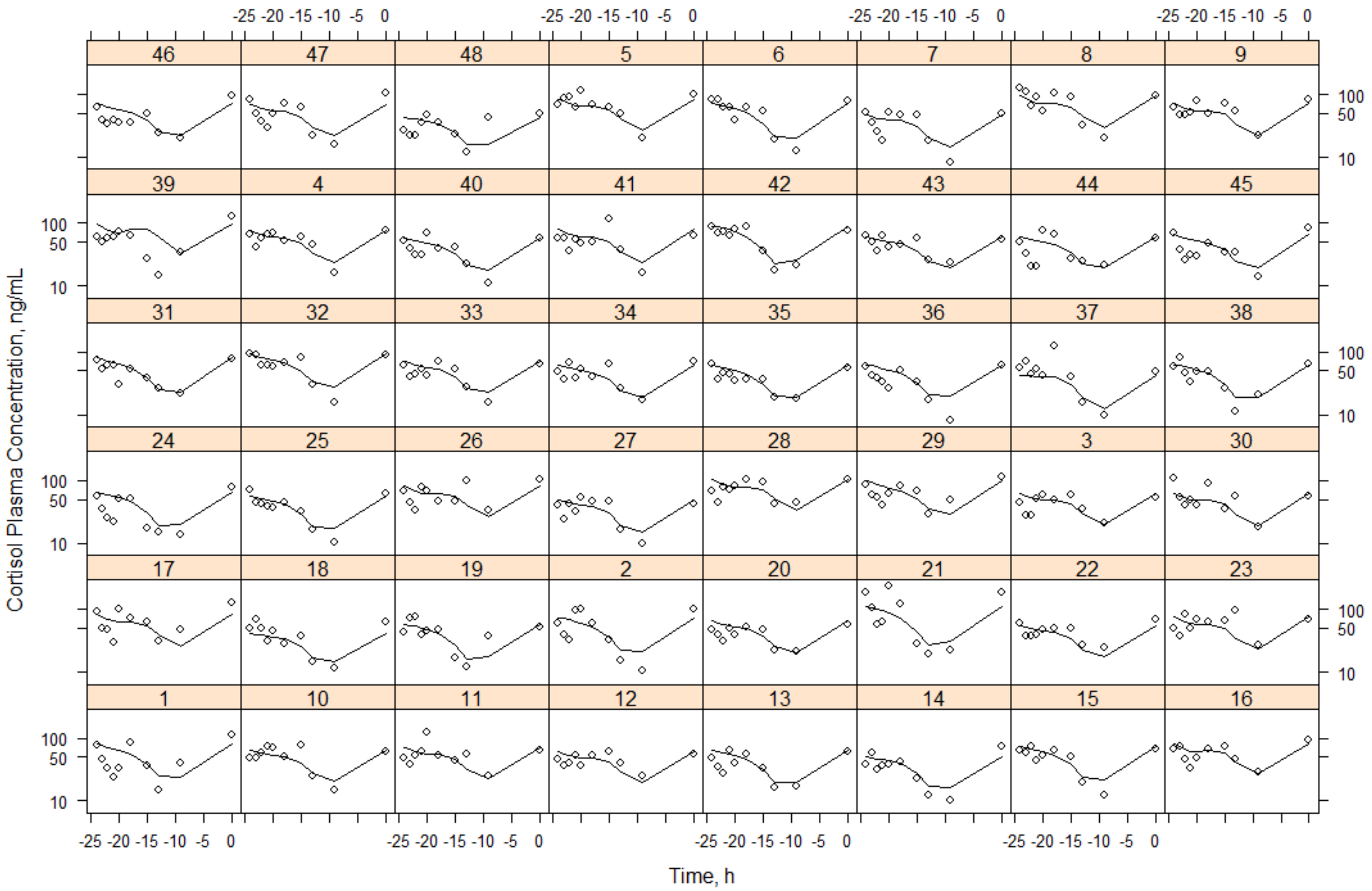

Figure 2S. Individual baseline cortisol plasma concentrations that were observed (symbols) and predicted (lines) by the population PD model, Eqs. (1)-(7).

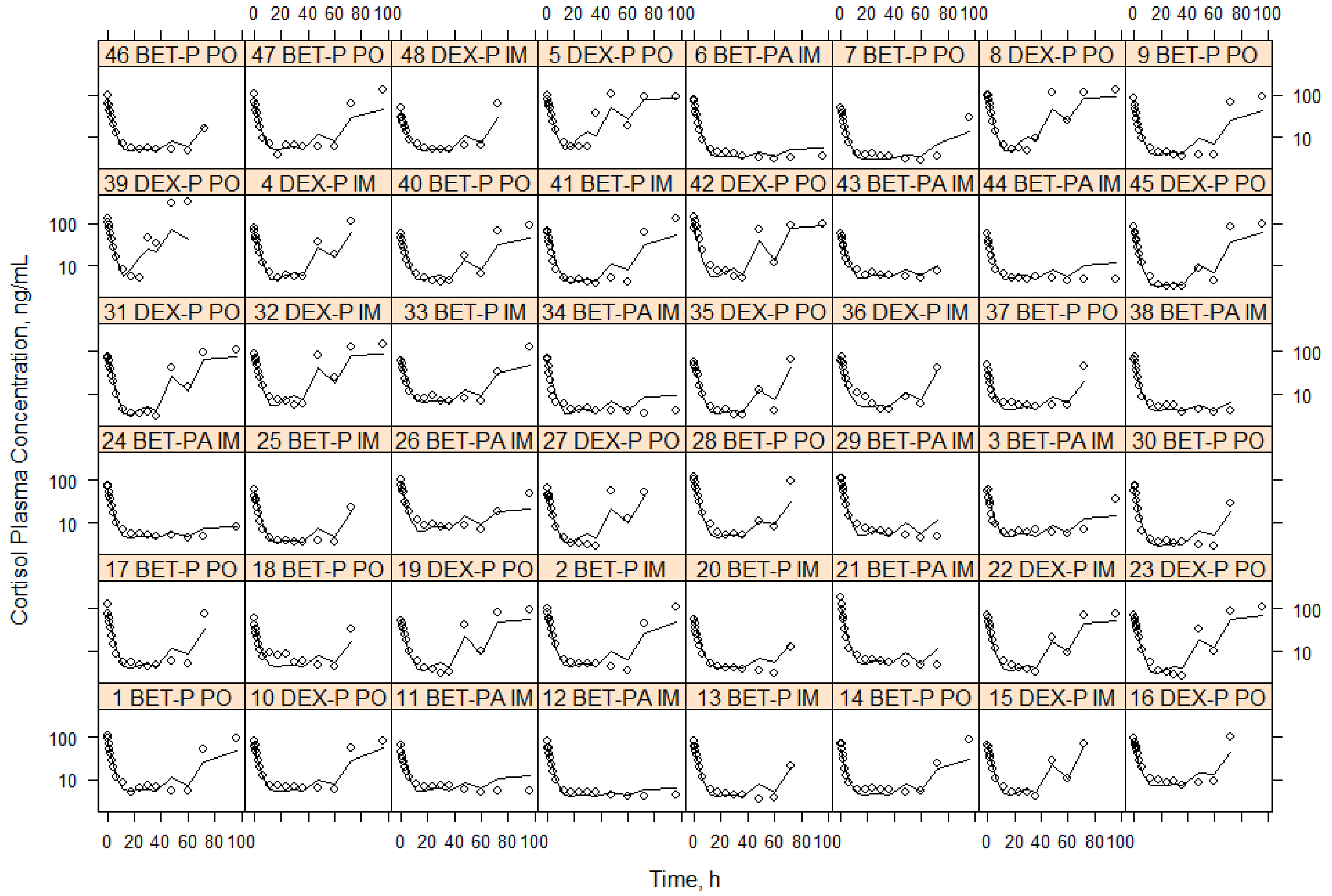

Figure 3S. Individual cortisol plasma concentrations that were observed (symbols) and predicted (lines) by the population PD model, Eqs. (1)-(7), during the first period. Subject numbers and administered drugs are indicated.

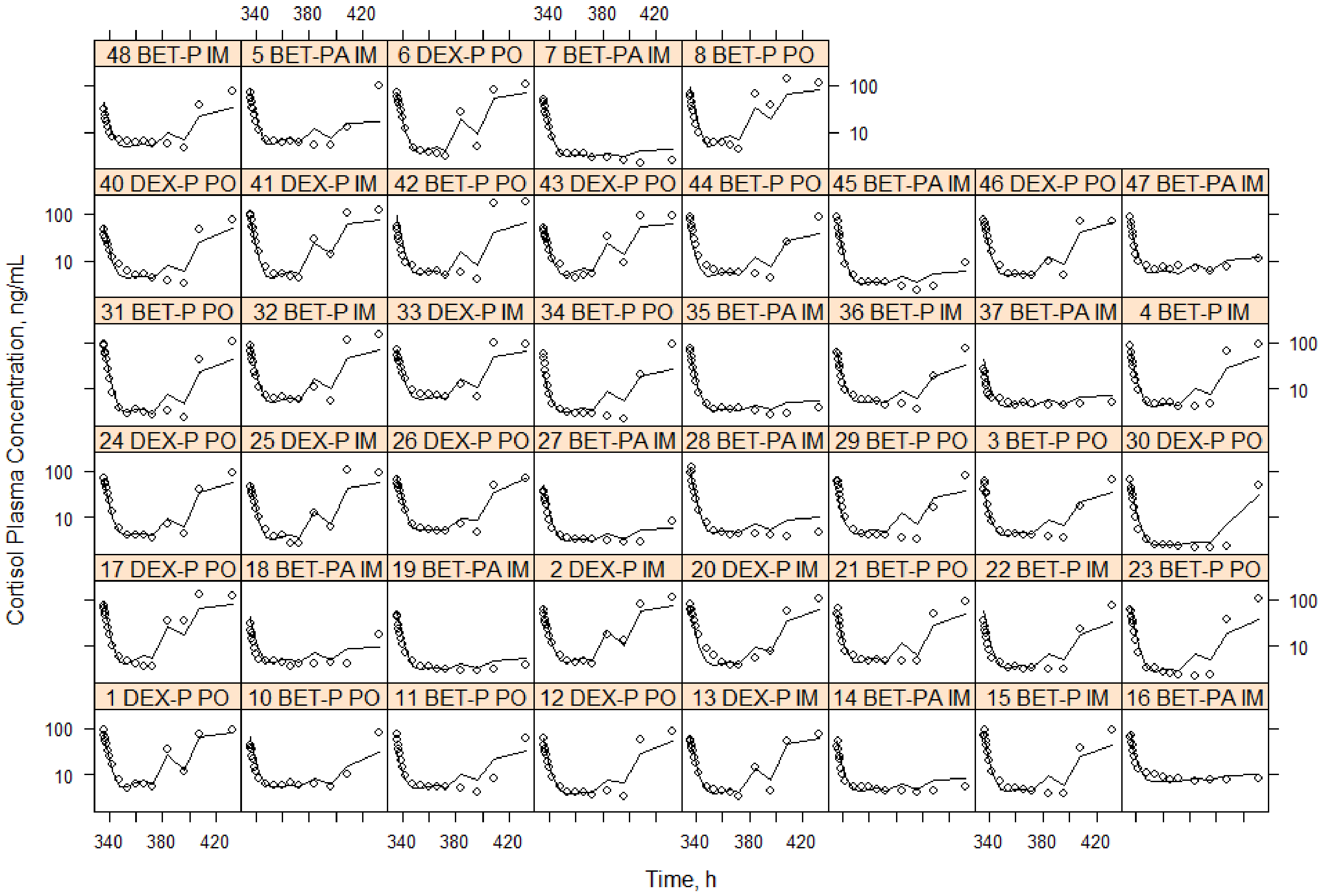

Figure 4S. Individual cortisol plasma concentrations that were observed (symbols) and predicted (lines) by the population PD model, Eqs. (1)-(7), during the second period.

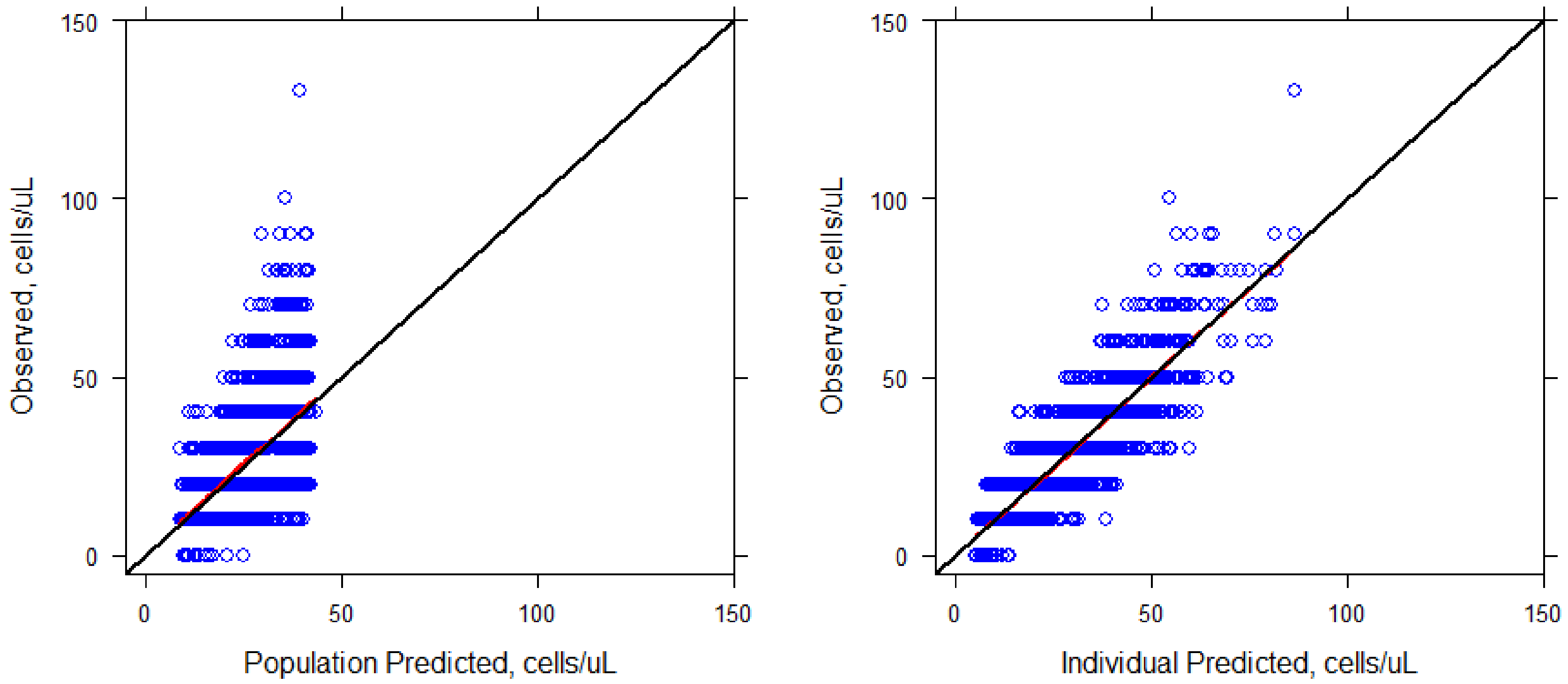

Figure 5S. Observed vs population predicted (left) and individual predicted (right) diagnostic plots for population PD model of basophils, Eqs. (8)-(12). Lines are as defined in Figure 1S

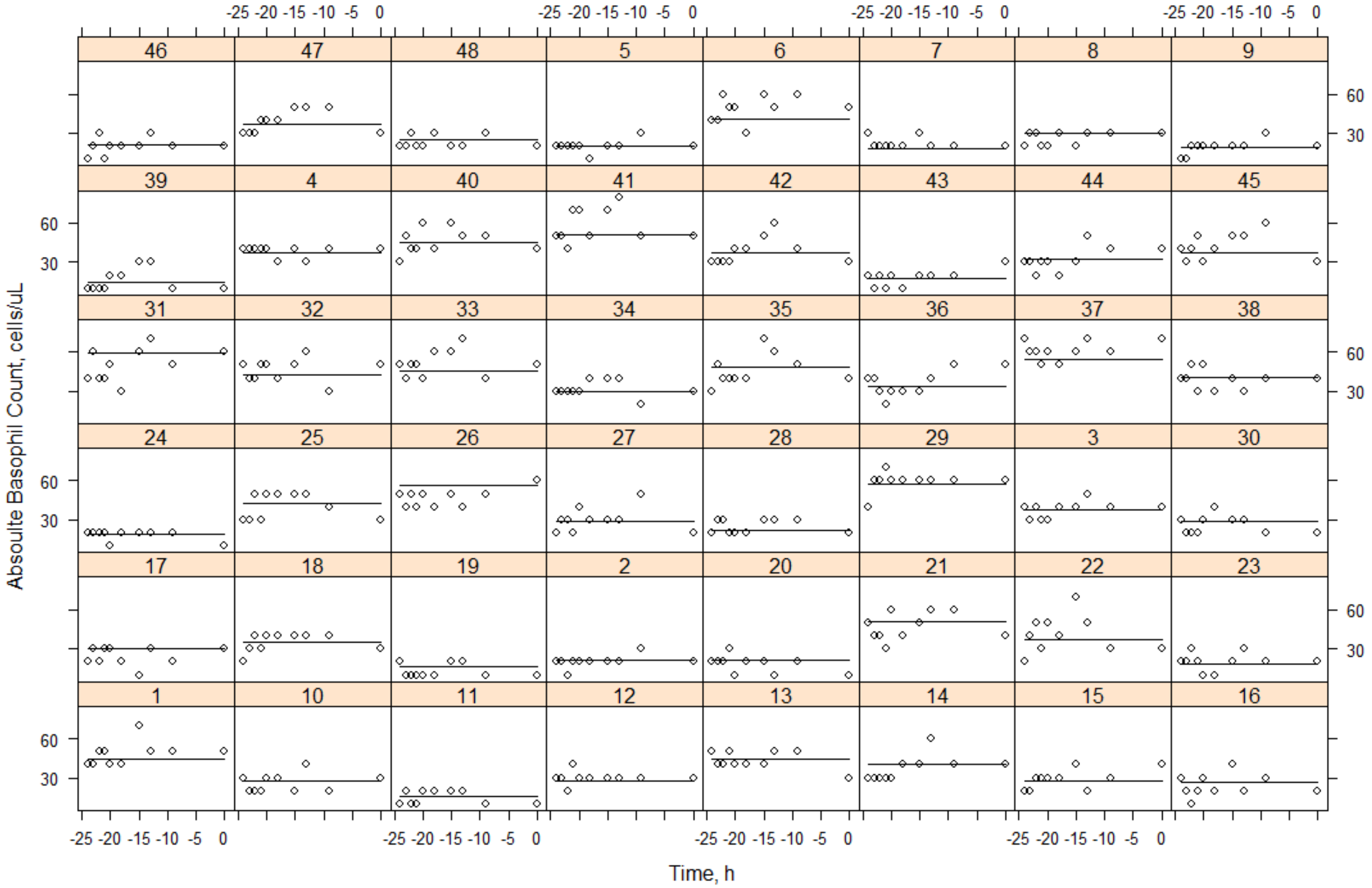

Figure 6S. Individual baseline absolute basophil counts that were observed (symbols) and predicted (lines) by the population PD model, Eqs. (8)-(12), for indicated subjects

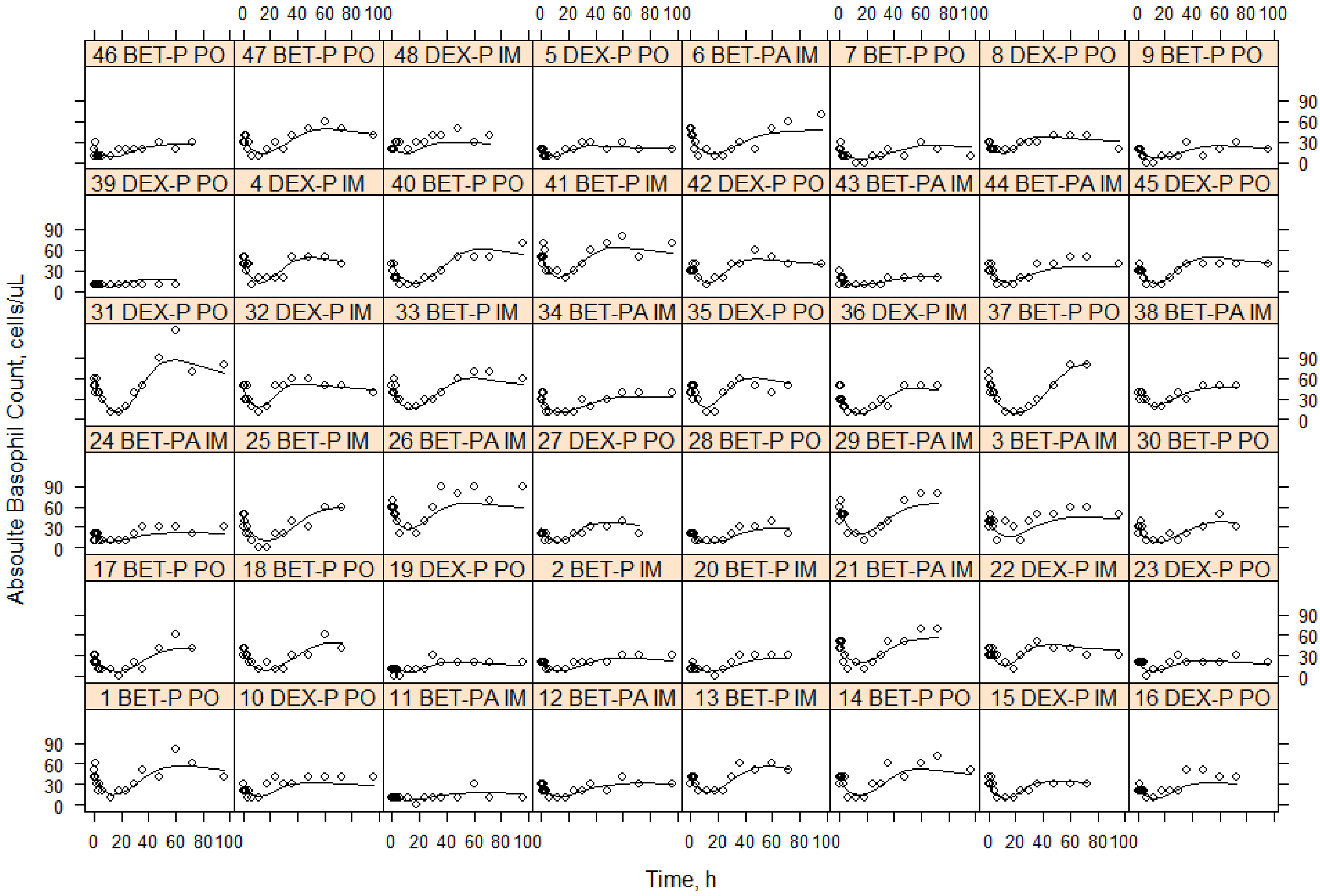

Figure 7S. Individual absolute basophil counts that were observed (symbols) and predicted (lines) by the population PD model Eqs. (8)-(12) during the first period for indicated subjects

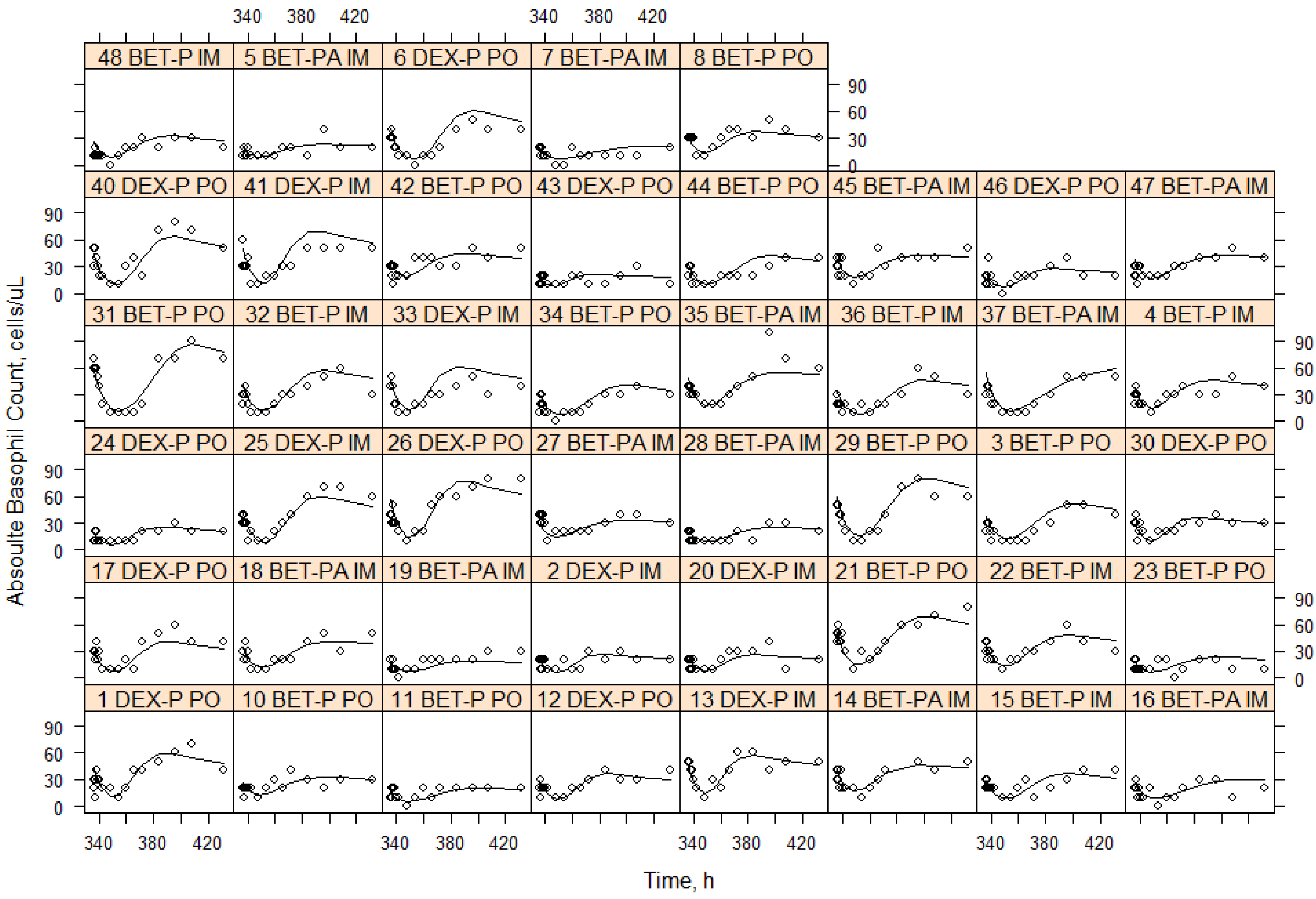

Figure 8S. Individual absolute basophil counts that were observed (symbols) and predicted (lines) by the population PD model, Eqs. (8)-(12), during the second period for indicated subjects.

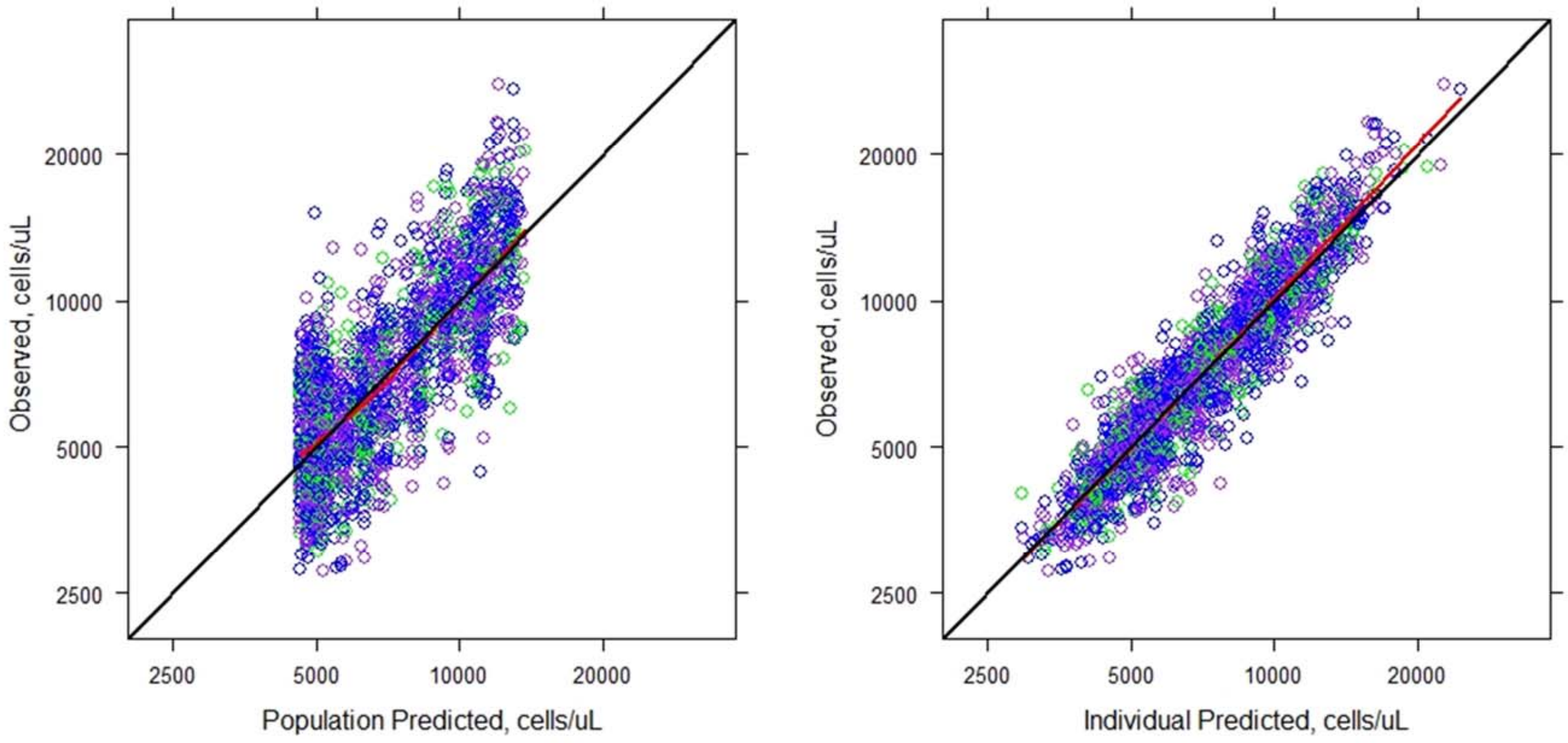

Figure 9S. Observed vs population predicted (left) and individual predicted (right) diagnostic plots for population PD model of neutrophils, Eqs. (13)-(18). Lines are as defined in Figure 2S. .

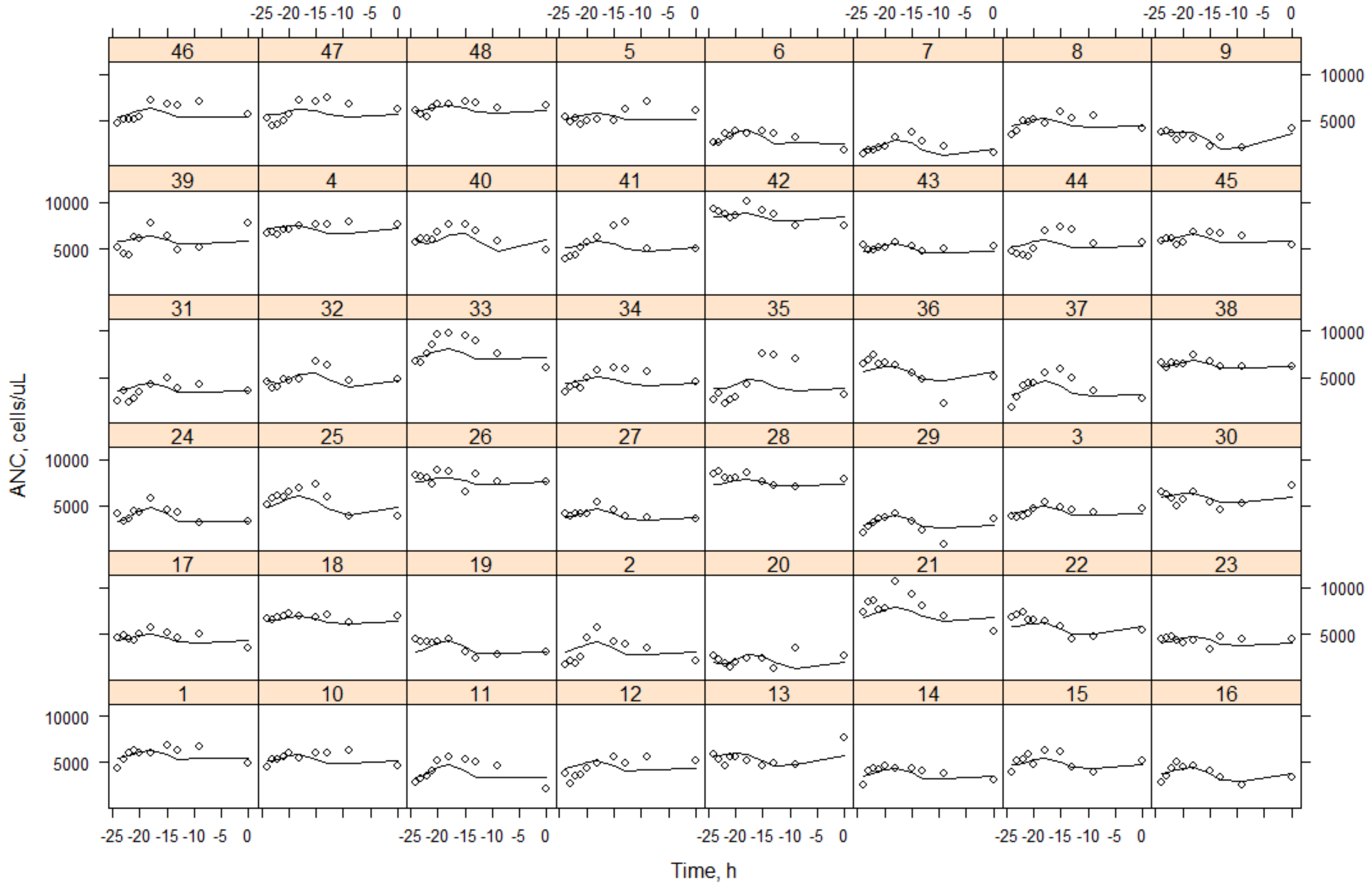

Figure 10S. Individual baseline absolute neutrophil counts that were observed (symbols) and predicted (lines) by the population PD model, Eqs. (13)-(18), in the indicated subjects.

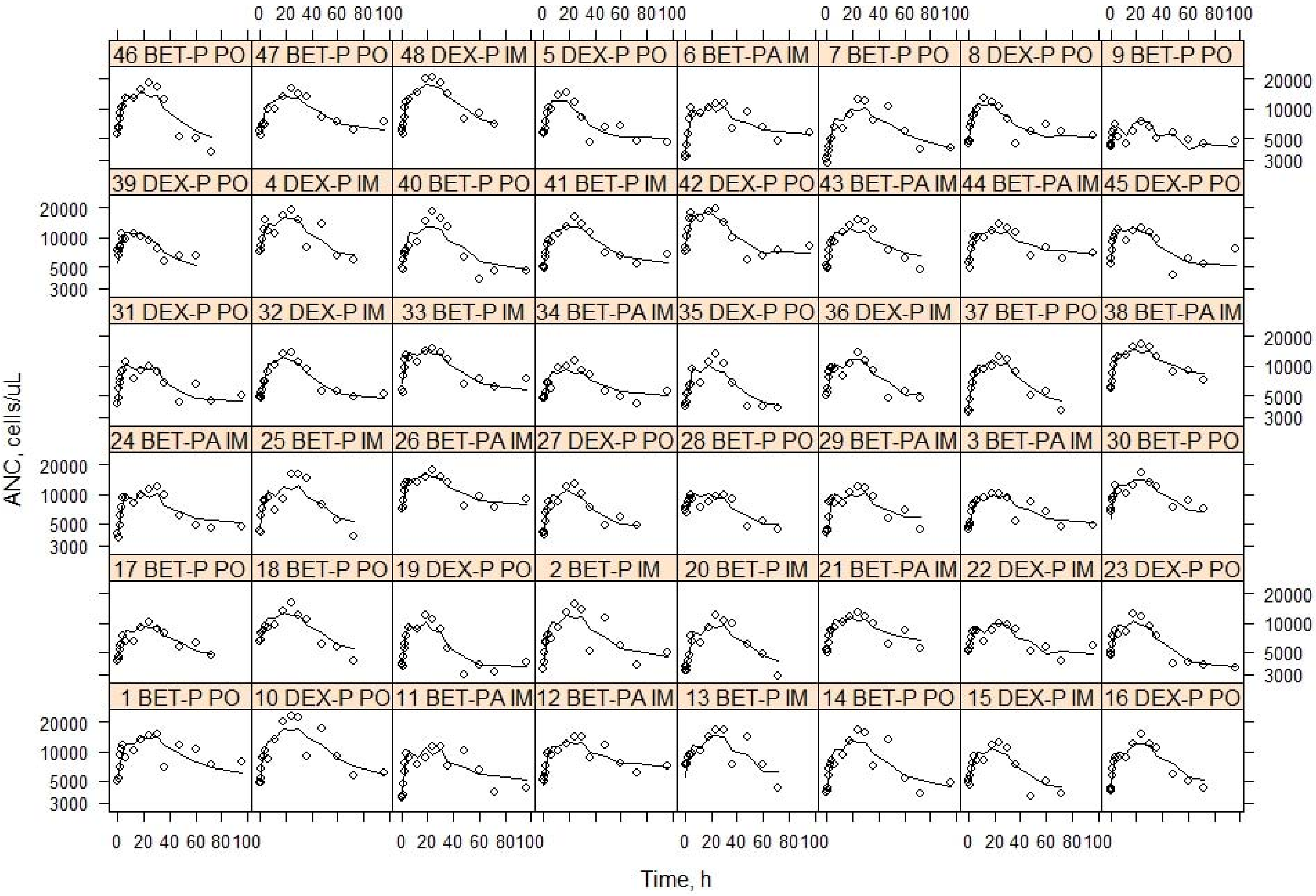

Figure 11S. Individual absolute neutrophil counts that were observed (symbols) and predicted (lines) by the population PD model, Eqs. (13)-(18), during the first period in the indicated subjects.

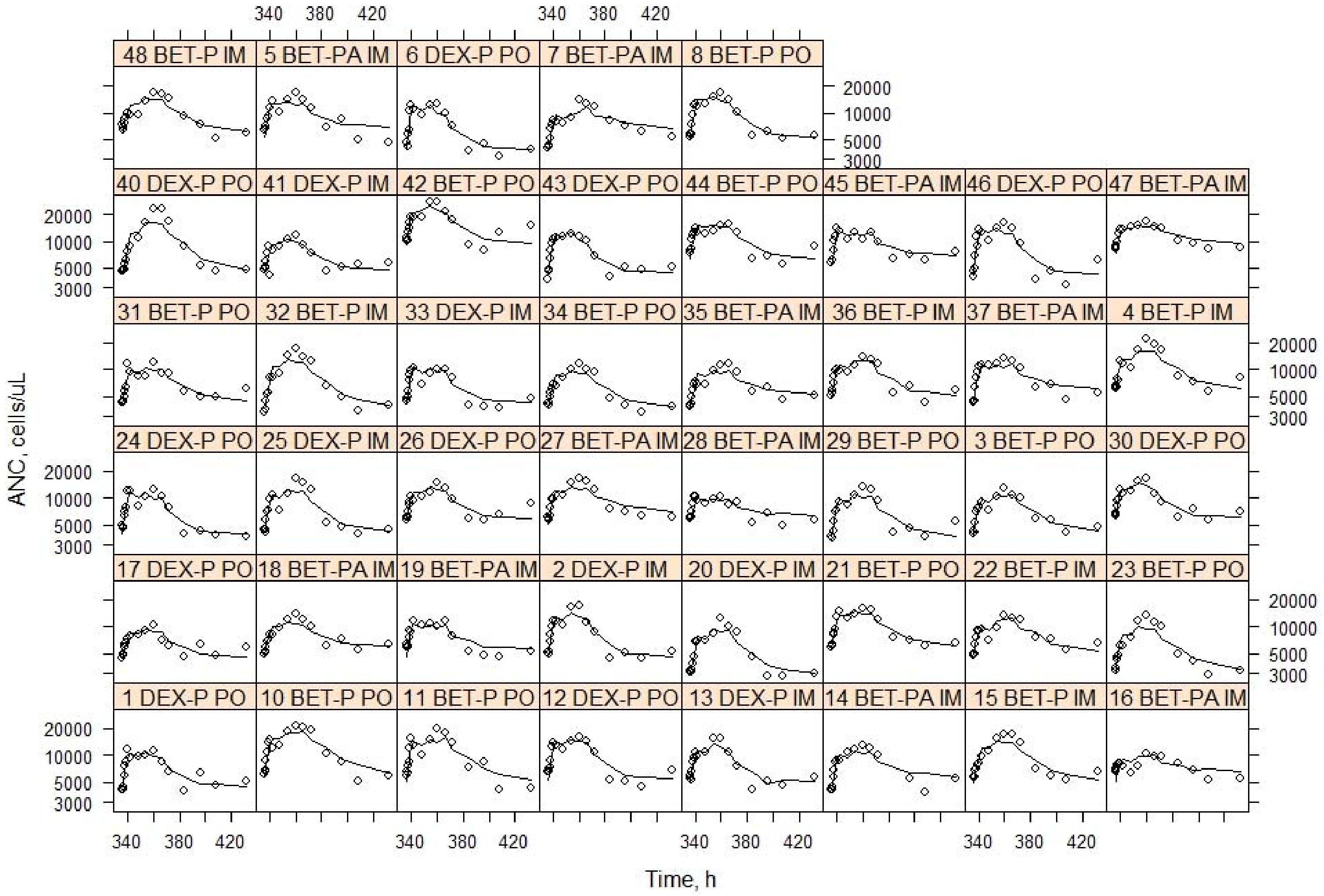

Figure 12S. Individual absolute neutrophil counts that were observed (symbols) and predicted (lines) by the population PD model, Eqs. (13)-(18), during the second period in the indicated subjects.

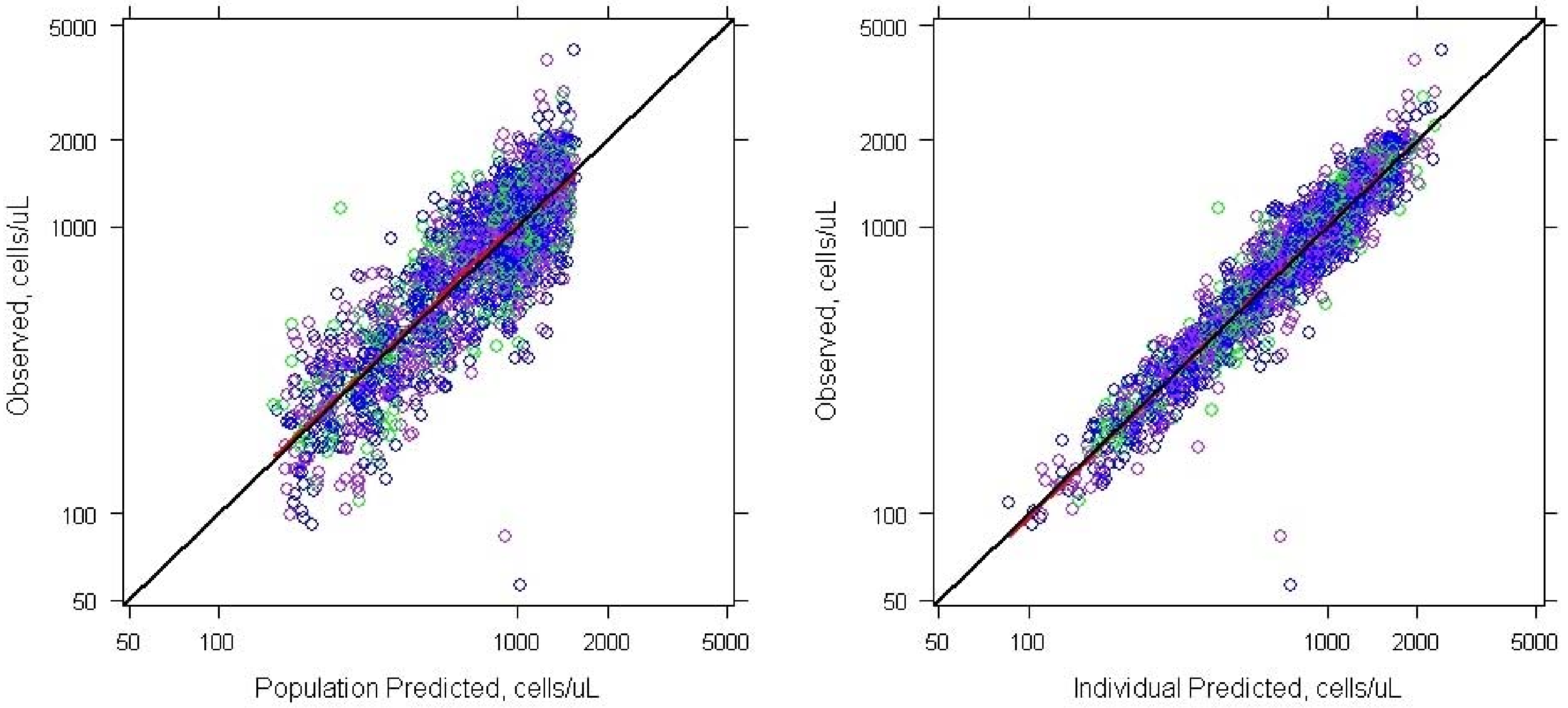

Figure 13S. Observed vs population predicted (left) and individual predicted (right) diagnostic plots for population PD model of T-helper cells Eqs. (19)-(21). Line are as defined in Figure 2S.

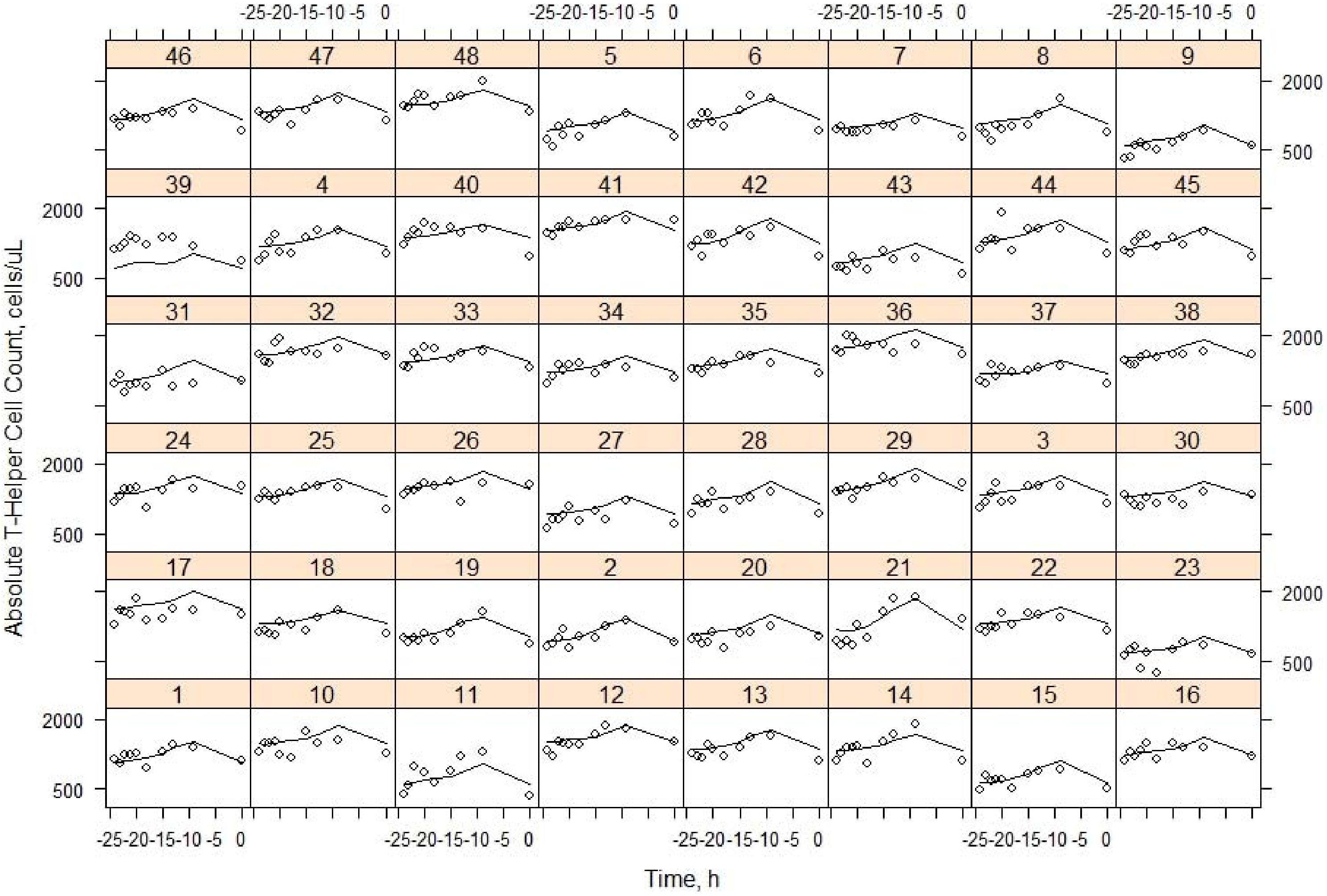

Figure 14S. Individual baseline absolute T-helper cell counts that were observed (symbols) and predicted (lines) by the population PD model, Eqs. (19)-(21), in the indicated subjects

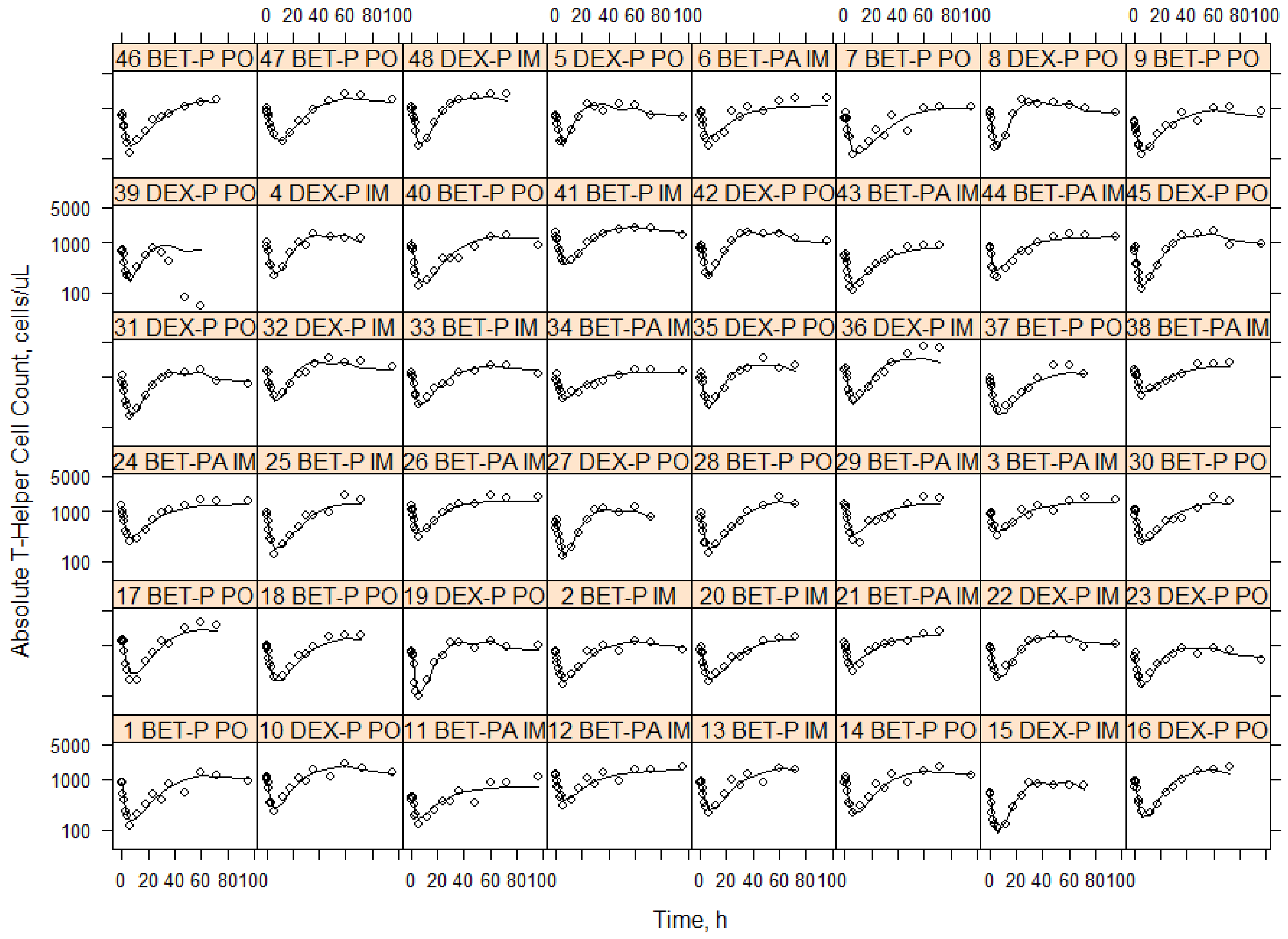

Figure 15S. Individual absolute T-helper cell counts that were observed (symbols) and predicted (lines) by the population PD model, Eqs. (19)-(21), during the first period in the indicated subjects.

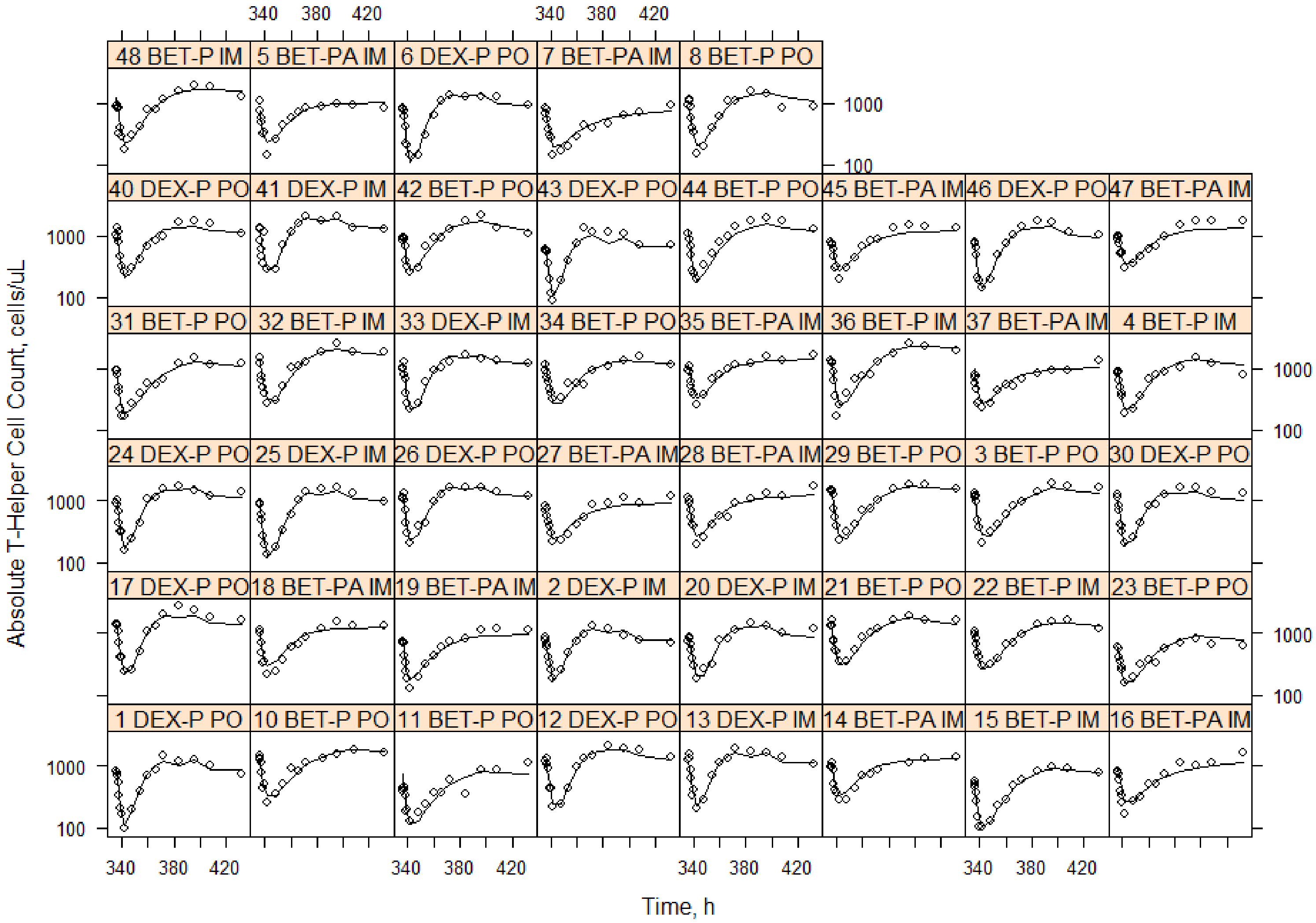

Figure 16S. Individual absolute T-helper cell counts that were observed (symbols) and predicted (lines) by the population PD model, Eqs. (19)-(21), during the second period in the indicated subjects.

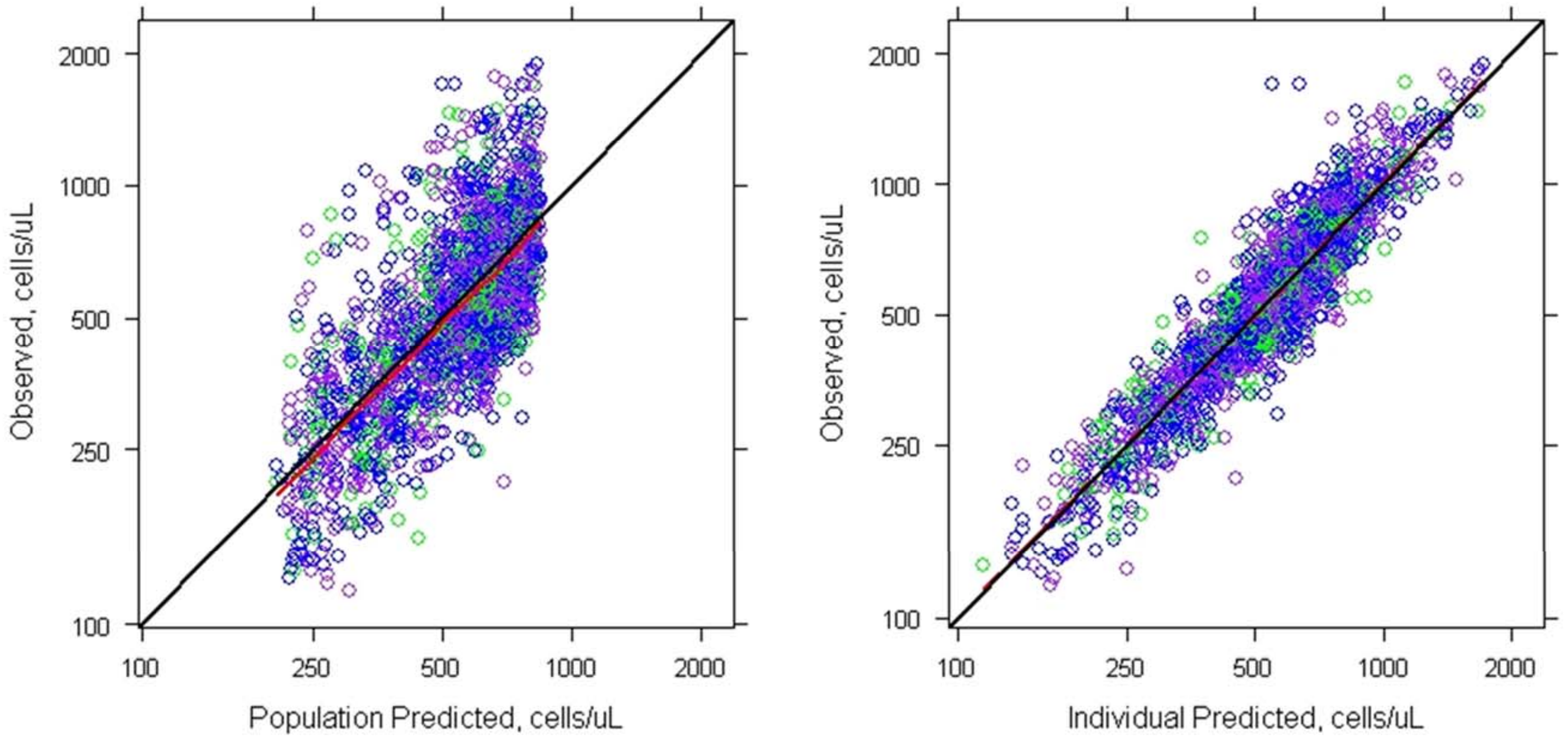

Figure 17S. Observed vs population predicted (left) and individual predicted (right) diagnostic plots for population PD model of T-cytotoxic cells, Eqs. (22)-(24). Lines are as defined in Figure 2S.

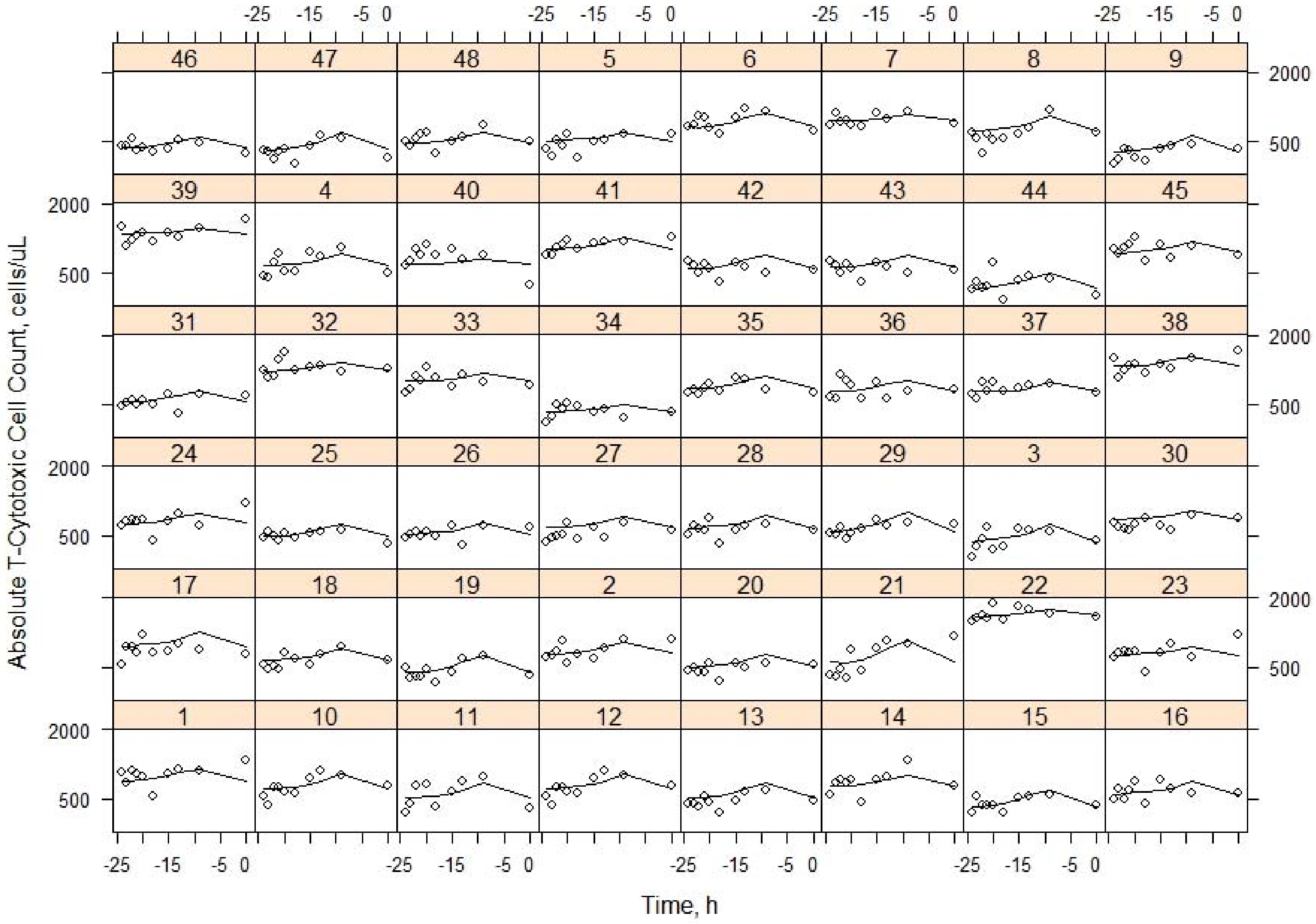

Figure 18S. Individual baseline absolute T-cytotoxic cell counts that were observed (symbols) and predicted (lines) by the population PD model, Eqs. (22)-(24), in the indicated subjects.

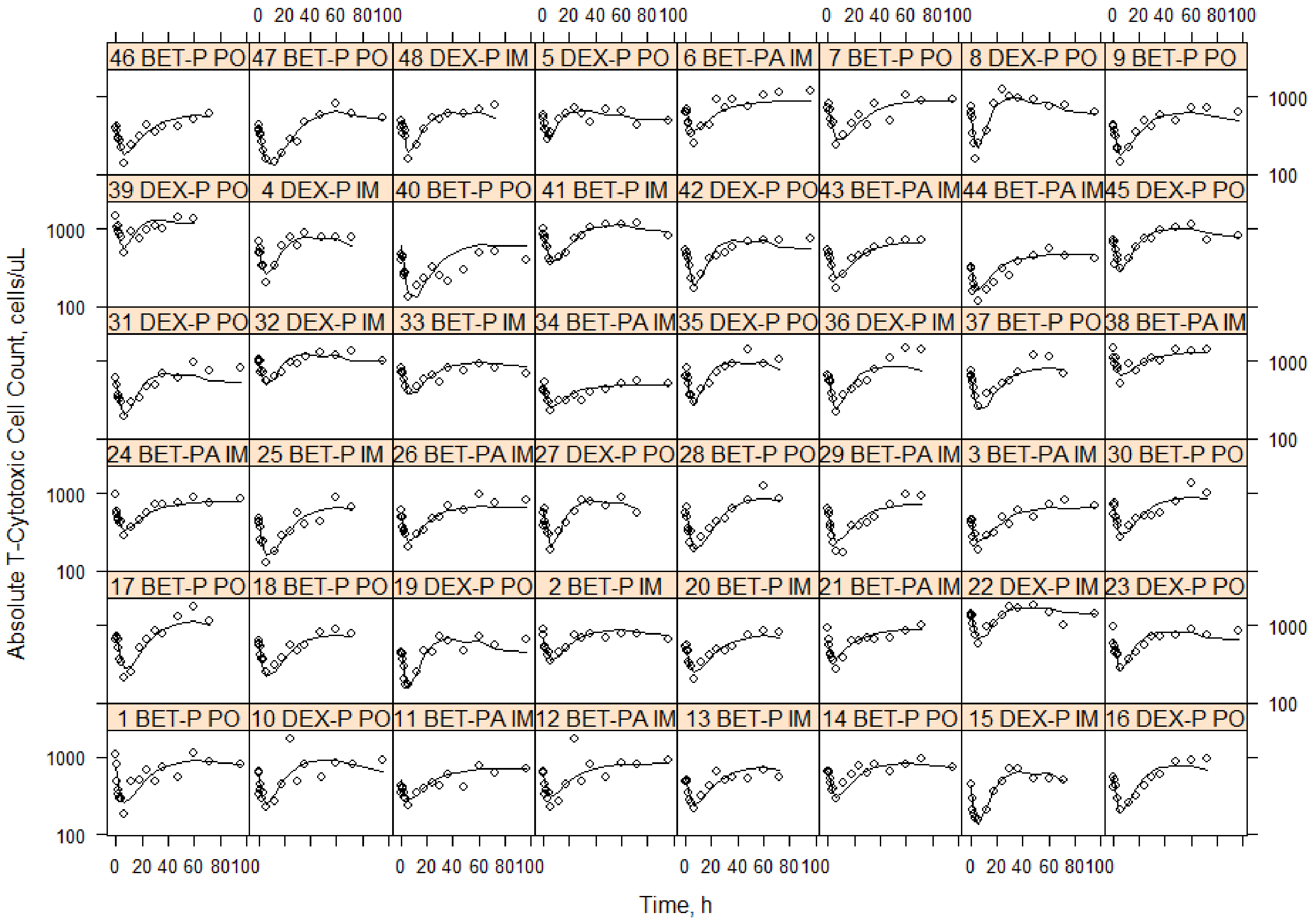

Figure 19S. Individual absolute T-cytotoxic cell counts that were observed (symbols) and predicted (lines) by the population PD model, Eqs. (22)-(24), during the first period in the indicated subjects.

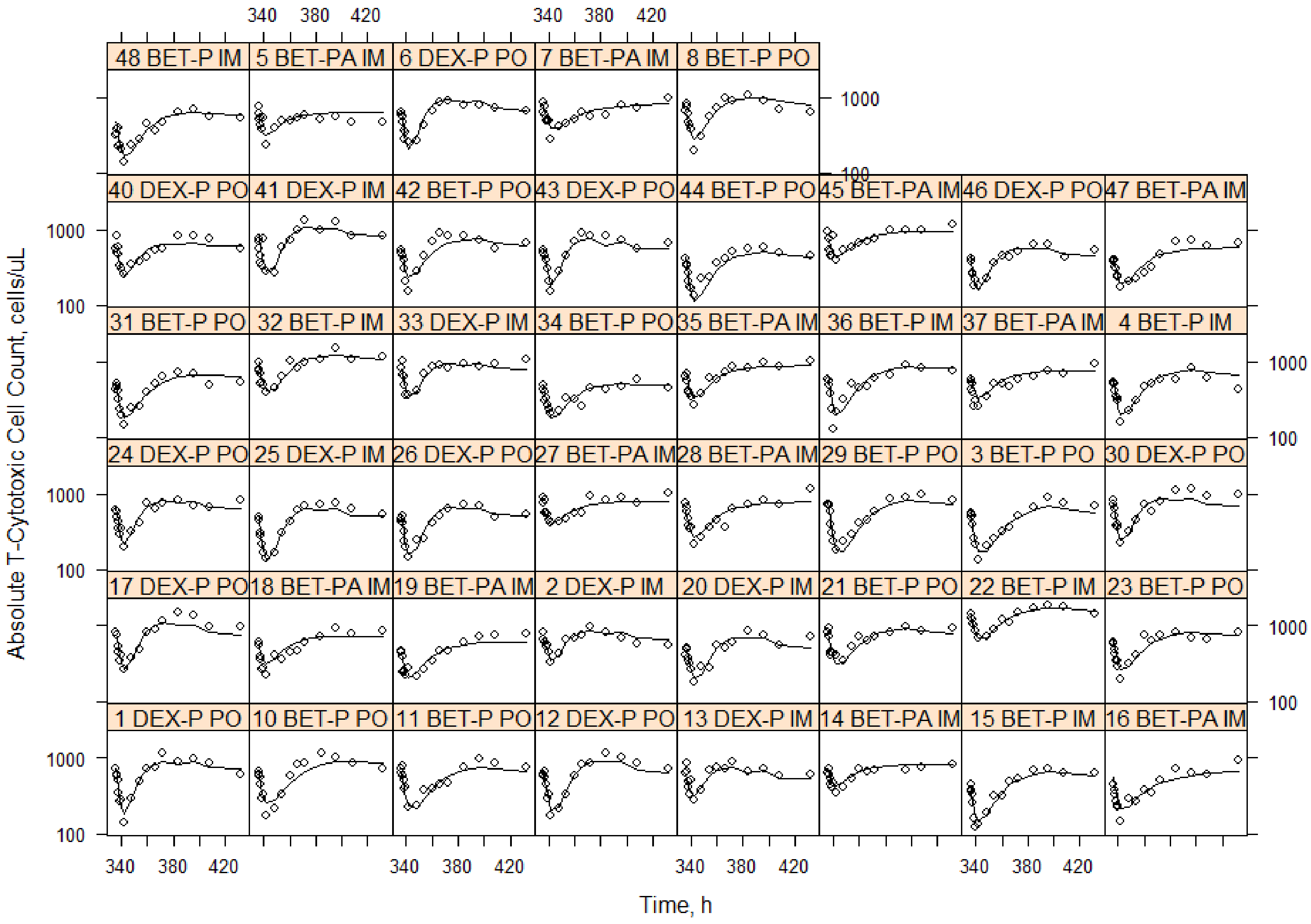

Figure 20S. Individual absolute T-cytotoxic cell counts that were observed (symbols) and predicted (lines) by the population PD model, Eqs. (22)-(24), during the second period in the indicated subjects.

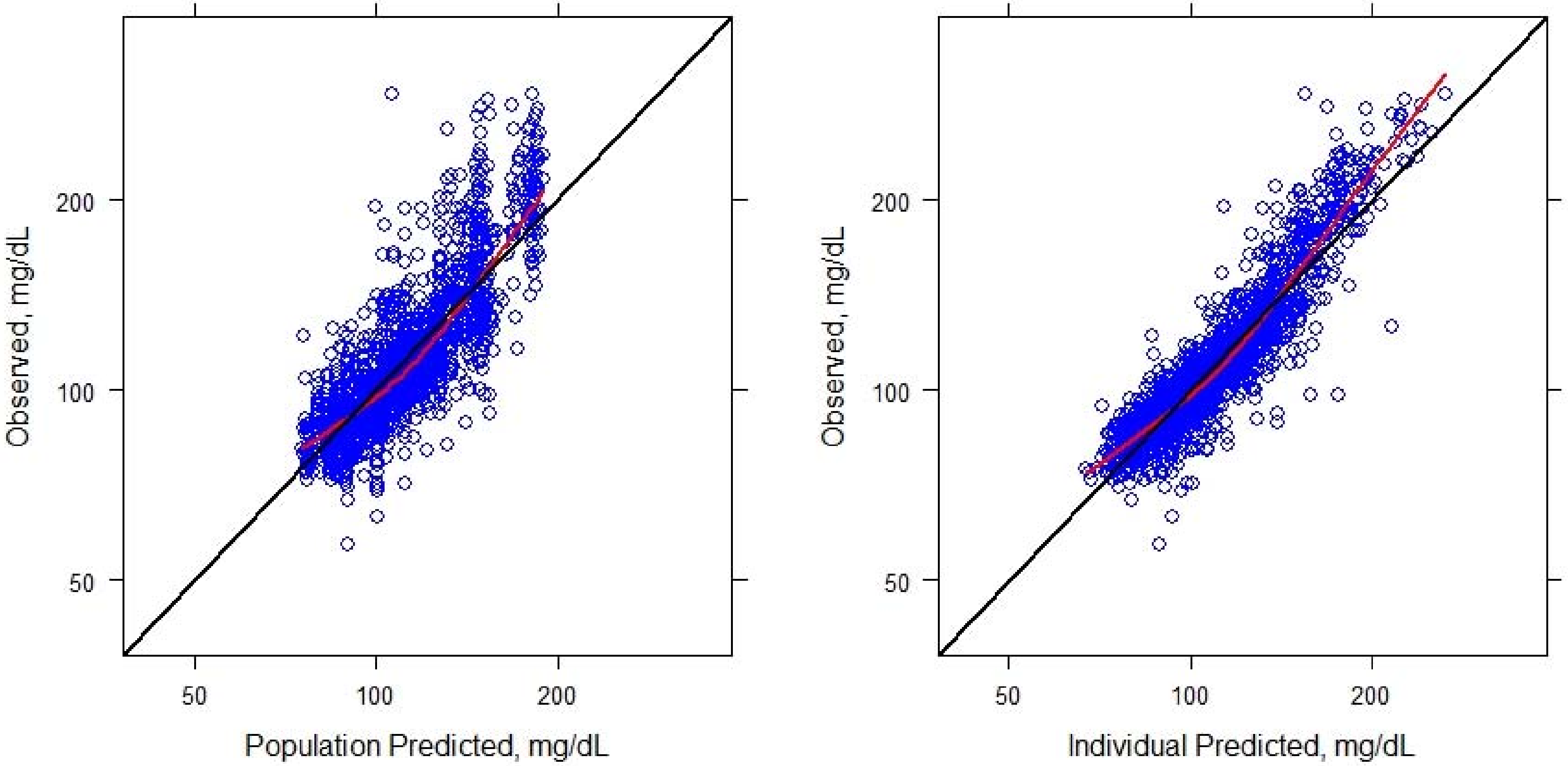

Figure 21S. Observed vs population predicted (left) and individual predicted (right) diagnostic plots for population PD model of glucose, Eqs. (25)-(29). The lines are as defined in Figure 2S.

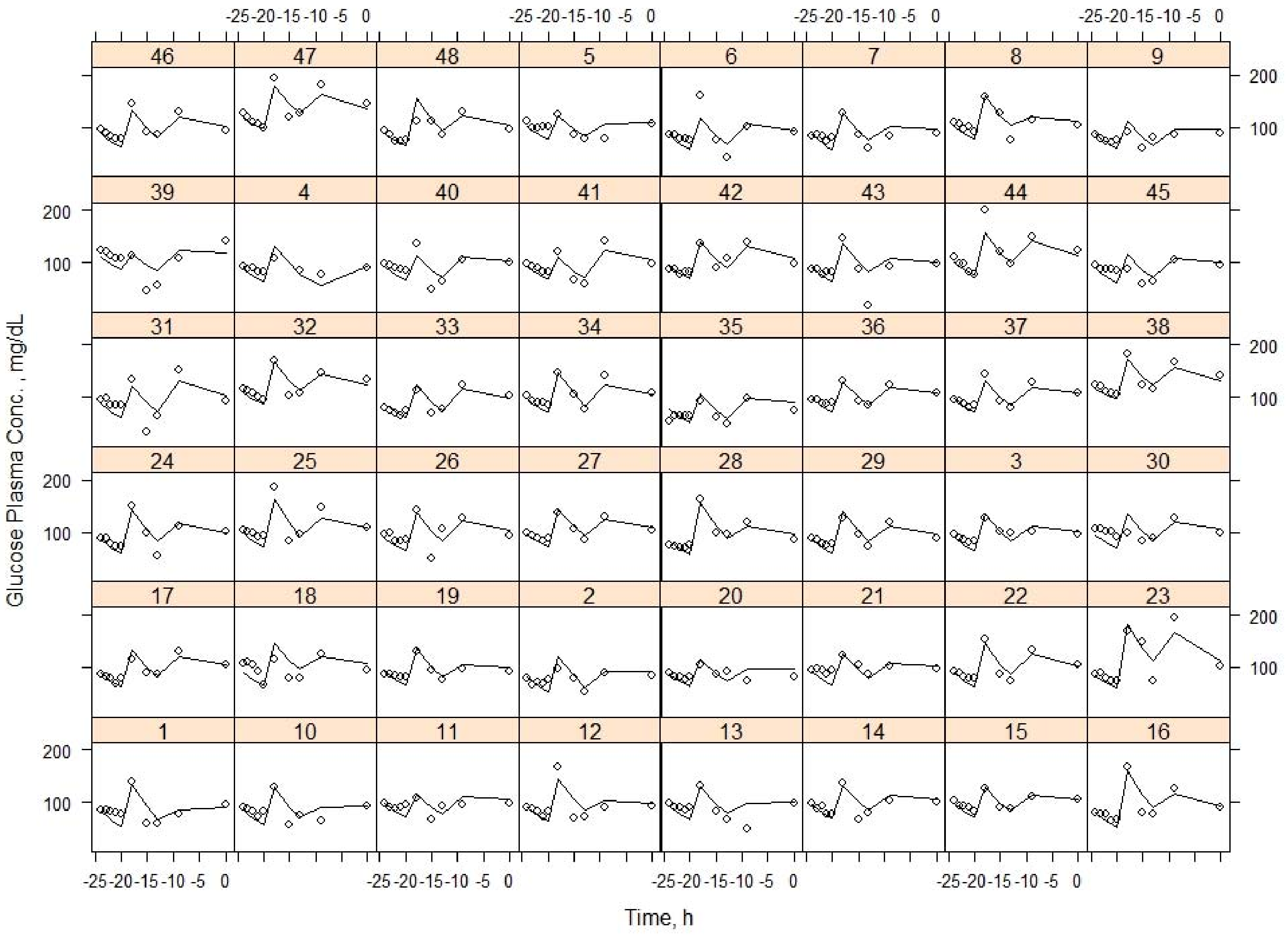

Figure 22S. Individual baseline glucose plasma concentrations that were observed (symbols) and predicted (lines) by the population PD model, Eqs. (25)-(29), in the indicated subjects

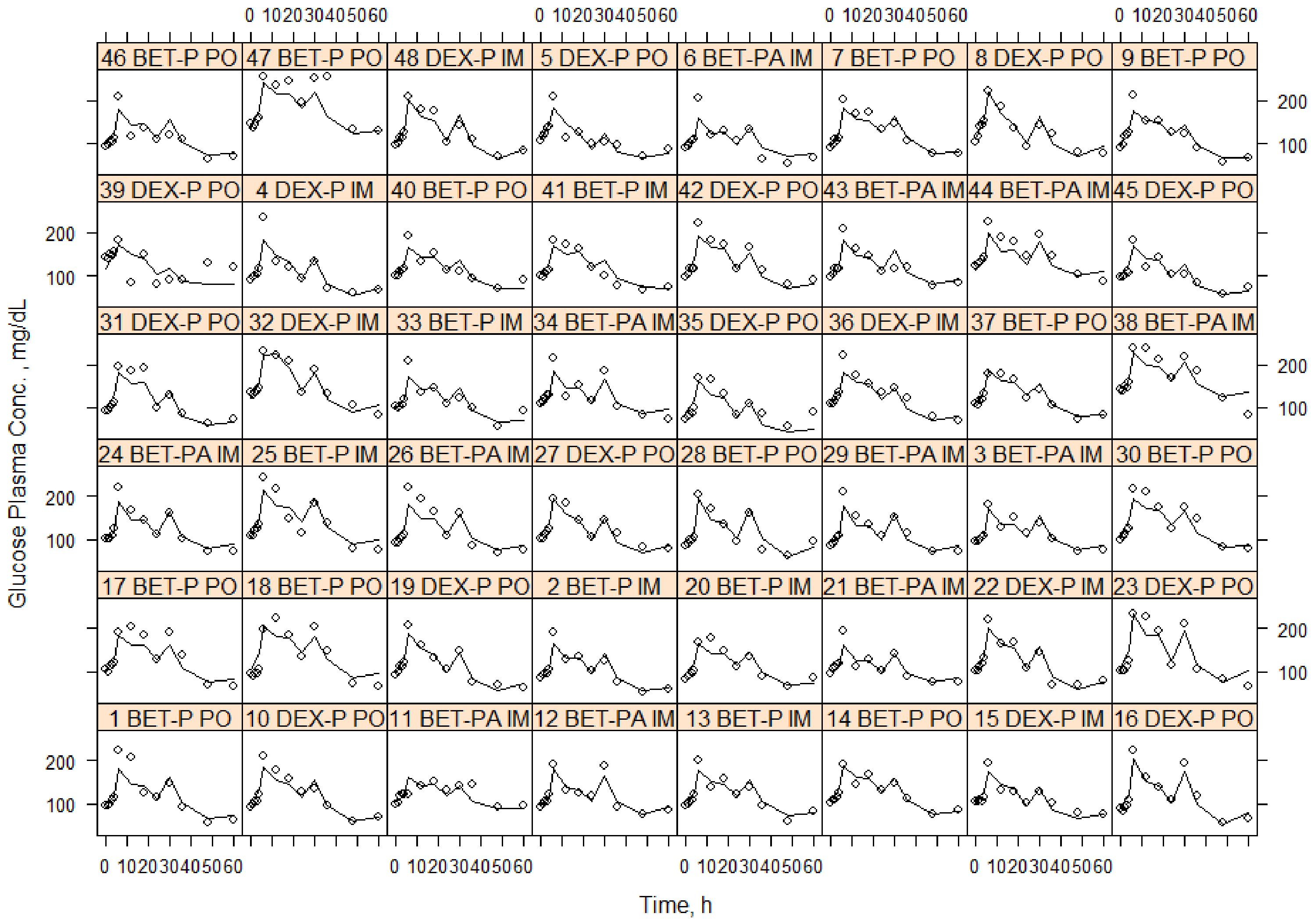

Figure 23S. Individual glucose plasma concentrations that were observed (symbols) and predicted (lines) by the population PD model, Eqs. (25)-(29), during the first period in the indicated subjects.

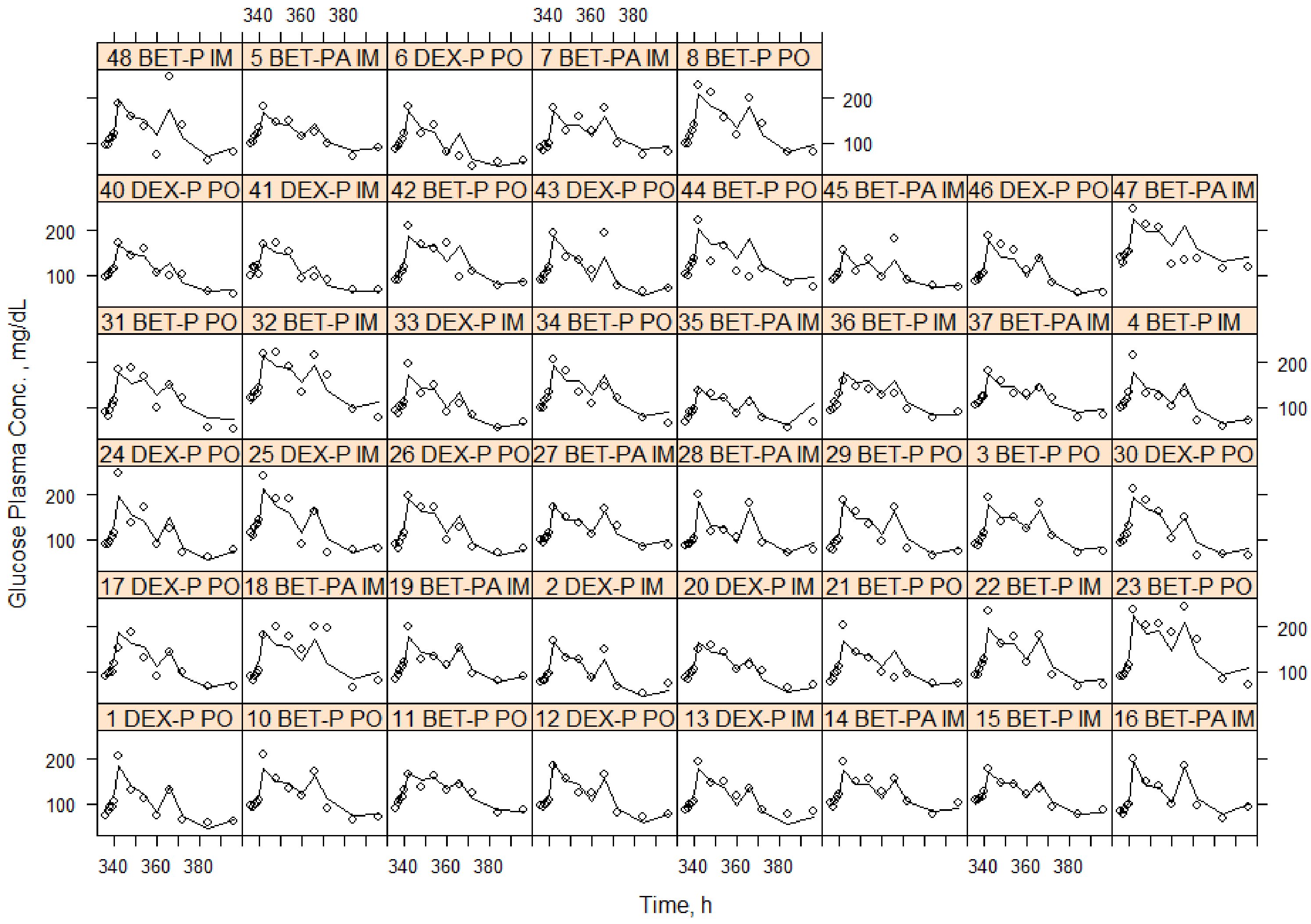

Figure 24S. Individual glucose plasma concentrations that were observed (symbols) and predicted (lines) by the population PD model, Eqs. (25)-(29), during the second period in the indicated subjects.

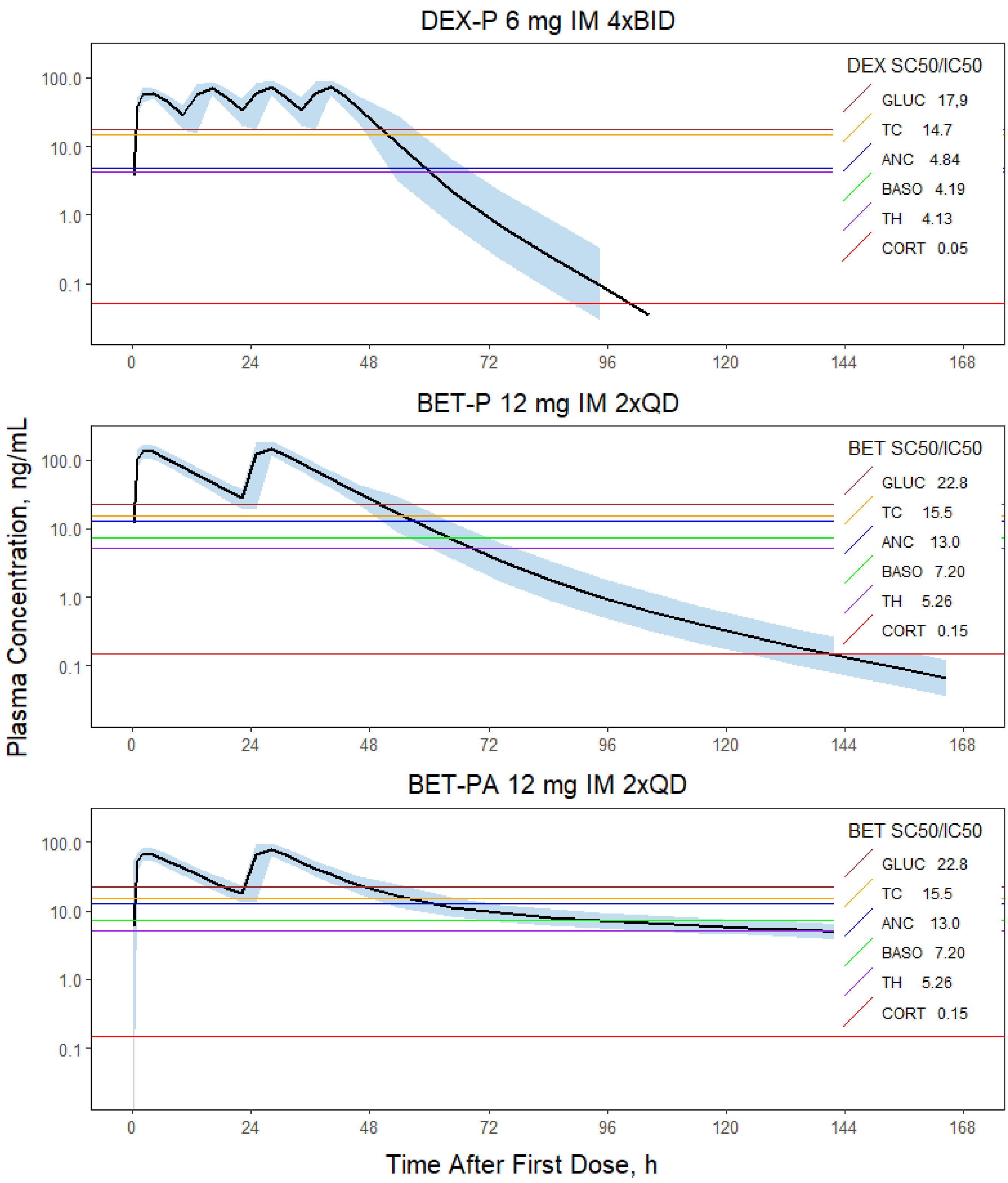

Figure 25S. Simulated GC plasma concentrations for indicated dosing regimens. The solid line is the median of N=200 subjects. The shaded regions represent 5th and 95th percentiles. The horizontal line depicts the typical values of sensitivity parameters for indicated responses.
